# Factors Associated with Dengue Shock Syndrome: A Systematic Review and Meta-Analysis

**DOI:** 10.1371/journal.pntd.0002412

**Published:** 2013-09-26

**Authors:** Nguyen Tien Huy, Tran Van Giang, Dinh Ha Duy Thuy, Mihoko Kikuchi, Tran Tinh Hien, Javier Zamora, Kenji Hirayama

**Affiliations:** 1 Department of Immunogenetics, Institute of Tropical Medicine (NEKKEN), Nagasaki University, Nagasaki, Japan; 2 BBB Laboratory, PharmaCo-Cell Co. Ltd., Nagasaki, Japan; 3 Center for International Collaborative Research, Nagasaki University, Nagasaki, Japan; 4 Wellcome Trust Major Overseas Programme, Oxford University Clinical Research Unit, Hospital for Tropical Diseases, Ho Chi Minh City, Vietnam; 5 Clinical Biostatistics Unit, Ramón y Cajal Hospital and CIBER Epidemiologia y Salud Publica (CIBERESP), Madrid, Spain; 6 Global COE program, Nagasaki University, Nagasaki, Japan; Pediatric Dengue Vaccine Initiative, United States of America

## Abstract

**Background:**

The pathogenesis of dengue shock syndrome (DSS, grade 3 and 4) is not yet completely understood. Several factors are reportedly associated with DSS, a more severe form of dengue infection that reportedly causes 50 times higher mortality compared to that of dengue patients without DSS. However, the results from these reports remain inconclusive. To better understand the epidemiology, clinical manifestation, and pathogenesis of DSS for development of new therapy, we systematically reviewed and performed a meta-analysis of relevant studies that reported factors in both DSS and dengue hemorrhagic fever (DHF, grade 1 and 2) patients.

**Methods and Findings:**

PubMed, EMBASE, Scopus, Google Scholar, Dengue Bulletin, Cochrane Library, Virtual Health Library, and a manual search of reference lists of articles published before September 2010 were used to retrieve relevant studies. A meta-analysis using fixed- or random-effects models was used to calculate pooled odds ratios (OR) or event rate with corresponding 95% confidence intervals. Assessment of heterogeneity and publication bias, meta-regression analysis, subgroup analysis, sensitivity analysis, and analysis of factor-specific relationships were further performed. There were 198 studies constituting 203 data sets that met our eligibility criteria. Our meta-regression analysis showed a sustained reduction of DSS/dengue hemorrhagic fever (DHF) ratio over a period of 40 years in Southeast Asia, especially in Thailand. The meta-analysis revealed that age, female sex, neurological signs, nausea/vomiting, abdominal pain, gastrointestinal bleeding, hemoconcentration, ascites, pleural effusion, hypoalbuminemia, hypoproteinemia, hepatomegaly, levels of alanine transaminase and aspartate transaminase, thrombocytopenia, prothrombin time, activated partial thromboplastin time, fibrinogen level, primary/secondary infection, and dengue virus serotype-2 were significantly associated with DSS when pooling all original relevant studies.

**Conclusions:**

The results improve our knowledge of the pathogenesis of DSS by identifying the association between the epidemiology, clinical signs, and biomarkers involved in DSS.

## Author Summary

Dengue is one of the most common viral diseases transmitted by infected mosquitoes. It may range from asymptomatic or self-limiting dengue fever (DF) to severe dengue characterized by plasma leakage (dengue hemorrhagic fever, DHF) and dengue shock syndrome (DSS). Death from dengue infection occurs mostly in DSS, and the mortality of DSS is reportedly 50 times higher compared to that of dengue patients without DSS. Several factors associated with DSS have been reported in individual studies; however, the associations for some factors are not observed consistently across studies. Therefore, we conducted a systematic review of the literature to clarify this issue. The study showed persons with younger age, female sex, neurological signs, nausea/vomiting, abdominal pain, gastrointestinal bleeding, increased hemoconcentration, ascites, pleural effusion, hypoalbuminemia, hypoproteinemia, hepatomegaly, increased level of ALT or AST, thrombocytopenia, coagulation dysregulation, secondary infection, and infection of dengue virus serotype 2 are more likely to have DSS. This result improves our knowledge of the clinical manifestation and pathogenesis of DSS.

## Introduction

Dengue infection is a major health problem in tropical and subtropical countries. Each year, more than 250,000 cases of DHF/DSS are reported from an estimated 50 million dengue infections [1]. Dengue disease ranges from asymptomatic or self-limiting dengue fever (DF) to severe dengue characterized by plasma leakage (dengue hemorrhagic fever [DHF], grades 1 and 2) that can lead to a life-threatening syndrome (dengue shock syndrome [DSS], grades 3 and 4) [2]. Recently, severe dengue was also defined by severe bleeding and/or severe organ impairment [3]. Fatal cases of dengue infection mostly occur in patients with DSS, and the mortality of DSS is reportedly 50 times higher than that of dengue patients without DSS [4]. There are no licensed vaccines or antiviral drugs against the disease, although some potential solutions are currently being studied [5]. Early appropriate treatment, vector control, and educational program are the only current methods to reduce mortality and global disease burden [3], [6], [7], [8]. Therefore, the World Health Organization (WHO) encourages research based around markers of severity to develop new tools and methods that can reduce the healthcare burden of dengue infection in endemic countries.

Several factors associated with DSS have been reported in individual studies [9], [10], [11]; however, the associations for some factors are not observed consistently across studies [12], [13], [14], [15]. Therefore, we conducted a systematic review and meta-analysis of relevant studies to assess all reported factors associated with DSS.

## Methods

### Search strategy and study selection

Our study was performed according to the recommendations of the PRISMA statement [16], which is available in supporting information (Checklist S1). We had developed a protocol of methods from June to August 2010, and our protocol can be assessed in our homepage at: http://www.tm.nagasaki-u.ac.jp/hiraken/member/file/n_tien_huy/protocol_of%20systemic_review_for_dengue3.pdf.

In September 2010, PubMed, Scopus, EMBASE, LILACS via Virtual Health Library, Google Scholar, WHO Dengue bulletin, Cochrane Library, and a manual search of reference lists of articles were searched for suitable studies. The search terms used for PubMed, EMBASE and Scopus were as follows: “dengue AND (shock OR DSS OR severity OR severe OR “grade IV” OR “grade III”)”. We used “dengue” to search in LILACS and Cochrane Library. For the “Advanced Scholar Search”, we used “dengue” to fill in the field “with all of the words”, “shock OR DSS OR severity OR severe OR “grade IV” OR “grade III”” to fill in the field “with at least one of the words”, and “where my words occur” in the field “title of article”.

**Figure 1:**
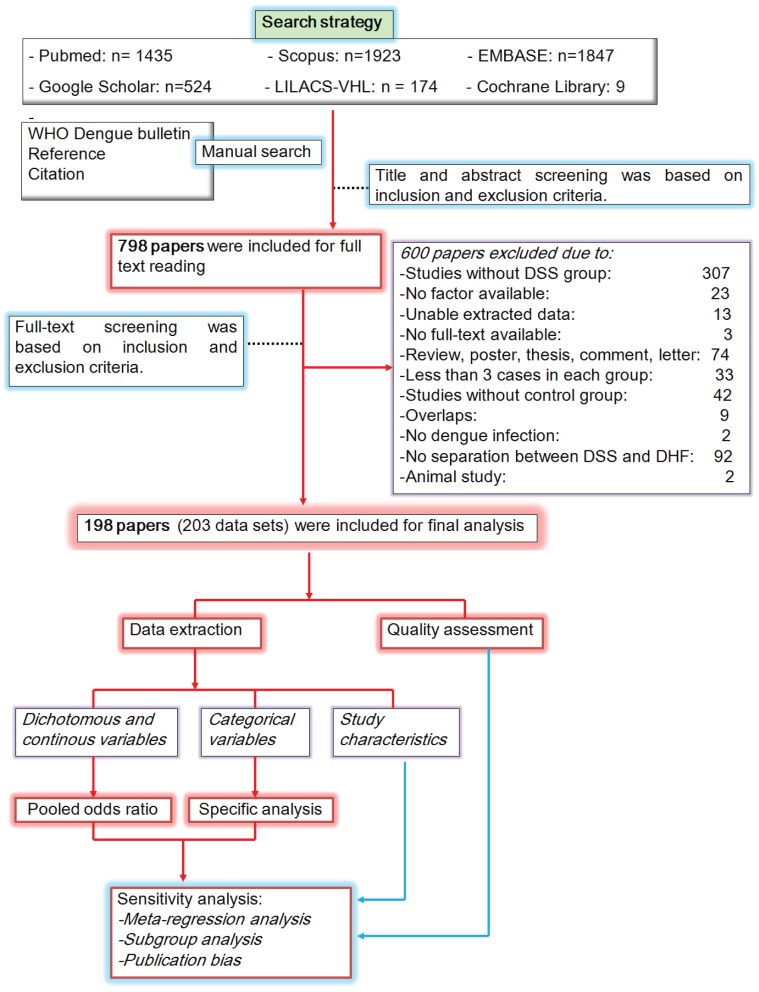
Flow diagram of the search and review process.

Two independent reviewers (NTH, TVG) initially scanned primary titles and abstracts (when available) to select potential full text articles for further scrutiny according to the inclusion and exclusion criteria. The inclusion criteria were as follows: articles with reported epidemiology, clinical signs, and laboratory parameters for dengue-infected patients with shock compared with DHF. Since genetic markers are rarely reported for DSS groups [17], [18], we did not include these markers in this study. We used broad criteria made by original studies' authors for definition of dengue infection, DSS, and DHF to increase the number of studies included in our analysis. A subgroup analysis was used to investigate the effect of this variation on the pooled results. No restrictions were applied with respect to language, gender, patient age (children or adult), or study design. Non-English reports were translated into English by authors with the help of native international students in Nagasaki University. The exclusion criteria are shown in Figure 1.

When the title and abstract were not rejected by either reviewer, the full text of the article was obtained via Nagasaki University Library and carefully reviewed for inclusion by the two reviewers (NTH, TVG). Inclusion or exclusion of each study was determined by discussion and consensus between the two reviewers. When disagreement occurred, a consensus decision was made following discussion with a third reviewer (DHDT).

We further supplemented these searches with a manual search of articles in the WHO Dengue Bulletin, reference lists, and citation lists using the Scopus databases. For each identified factor, we performed additional factor-specific searches by adding the factor terms beside “dengue”.

### Data extraction

Data were extracted by one of two investigators (NTH, TVG), and were checked by at least two of three reviewers (NTH, TVG, DHDT). Disagreement was resolved via discussion and a consensus reached between the three authors. A data extraction form in an Excel file was developed by two authors (NTH, TVG) based on a pilot review, extraction, and calibration of 20 randomly selected studies. The data extracted included the first author, year of publication, year of patient recruitment (the midpoint of the study's time period), study design (all case or case-control), data collection (prospective or retrospective), assignment of patients (consecutive or random), country and city of origin, hospital where the patients were recruited, characteristics of the patient population (infant, children, adult), criteria of dengue infection (confirmed or clinical diagnosis), criteria of DSS and DHF, number of included individuals (DSS and control DHF), including DF patients in the DHF group, description of blinded interpretation of factors, gender, and age at examination of included individuals. Several types of data input for factors were extracted if available and are fully described in the supplemental method (Method S1). Papers published by the same research group and studying the same factors were checked for potential duplicate data based on the year of patient recruitment and hospital where the patients were recruited. When duplications were noted, the largest data set was used for our meta-analysis.

When several types of data or several methods were presented for one particular factor, we extracted all data but used the one with the least significant association (the nearest odds ratio [OR] to one) if that factor was significantly associated with DSS after meta-analysis. Otherwise, data with lowest and highest ORs were pooled separately to get minimal and maximal odd ratios, respectively. When data were available on different days of the disease course, values at day 4, 3, 5, 2, and 6 were favored in that order for analysis, because shock frequently occurs at day 4 and we emphasize the importance of the transition from DHF to DSS.

### Quality assessment

Quality assessment was independently performed by two authors (NTH, TVG). The quality of selected studies was assessed using the combined criteria suggested by Pai et al. [19] and Wells et al. [20], because these criteria can affect the accuracy of the pooled effect size. The quality of each study included in the meta-analysis was determined across nine metrics: study design, full description of characteristic of patient population (infant, children, adult), data collection (prospective or retrospective), assignment of the patient (consecutive or random), inclusion criteria, exclusion criteria, method quality (description and same method for DSS and DHF groups), blinded interpretation of factors, and full description of dengue diagnosis. The score system is available in Table S1. Quality assessment was also performed by discussion and consensus after the independent review of each study by two authors (NTH, TVG).

### Meta-analyses

Meta-analyses for particular factors were performed using Comprehensive Meta-analysis software version 2·0 (Biostat, NJ, USA) where there was more than one study. Dichotomous and continuous variables were analyzed to compute pooled odds ratio (OR) and standardized mean difference, respectively when there were two groups of DSS and DHF. The standardized mean difference was then converted to OR according to the method of Borenstein *et al* [21]. Both dichotomous and continuous variables were combined to compute pooled odds ratio (OR) as previously suggested [22] to increase the number of studies included in our analysis. Therefore, the unit of continuous variables and the cut-off values of dichotomous and continuous variables were not required in the pooling result. Higher frequency of dichotomous variables and/or higher value of continuous variables in DSS compared to DHF groups resulted in a positive association of the particular variables with DSS. The event rate was pooled for the proportion of DSS among DHF/DSS cases. Only cross-sectional studies were included and DF patients in the DHF groups were excluded for this analysis of DSS prevalence. The corresponding 95% confidence intervals (95% CI) of pooled effect size were also calculated using a fixed-effects or random-effects model with weighting of the studies [23]. A fixed-effects model with weighting of the studies was used when there was a lack of significant heterogeneity (*p*>0.10), while a random-effects model with weighting of the studies was used when there was heterogeneity between studies (*p*≤0.10) [23].

Heterogeneity between studies was evaluated using the *Q* statistic and *I* ^2^-test. Heterogeneity was considered statistically significant if the *p*-value was <0·10 [24]. *I^2^* values>25%, 50%, or 75% were considered to represent low, moderate, or high heterogeneity, respectively [25].

To study the effect of covariates including total quality score, each parameter of quality score system, area of studies, and differences in definition of factors between studies (category/continuous variables, diagnosis, and unit of measurement) on the pooled effect size and the heterogeneity across studies, meta-regression analysis and subgroup analysis of a combination one or more groups were performed where there were eight or more studies assessing a particular factor [26]. The effect of covariates on the pooled effect size was considered significant when the *p*-value was <0·05 or its 95%CI did not overlap with the original one.

To evaluate the presence of publication bias, we performed Begg's funnel plot [27] and Egger's regression test [28], [29] when there were five or more studies assessing the association of a particular factors with DSS. Publication bias was considered significant when the p value was <0·1. If publication bias was found, the trim and fill method of Duvall and Tweedie was performed by adding studies that appeared to be missing [30], [31] to enhance the symmetry [26]. The adjusted pooled effect size and its 95% CI were computed after the addition of potential missing studies.

We further performed a sensitivity analysis by removing each study from the meta-analysis to investigate the effect of each study on the association. Cumulative meta-analysis was also carried out to test the effect of a few of the largest or smaller studies on the effect size by repeatedly performing a meta-analysis each time a new study was added according to its sample size (reversed variance of logOR or log of rate of event). The *p*-value for multiple comparisons was not adjusted because it may increase the likelihood of type II errors [32], [33]. Instead, to reduce the false discovery rate, a confidence interval, meta-regression, subgroup analysis, sensitivity analysis, and interpretation of across studies were conducted to give complement information to *p*-value. Thus, statistical significance was defined as *p*-value was <0.05 (two-tailed test) or its 95%CI did not overlap with the original one.

### Analysis of factor-specific relationships

Analysis of factor-specific relationships with DSS was also performed when there were three or more categories reported for a particular factor using the GraphPad Prism 5 (GraphPad Software, Inc., San Diego, CA, USA). This analysis was performed according to the dose-response relationship as previously reported [34], [35]. Briefly, the midpoint of each category for a particular factor was assigned to plot against the natural logarithm of OR (logOR) or rate of event. If no upper bound was available, we assumed it to be the same amplitude as the preceding category. When no lower bound was reported, we assigned it a value of zero. We used a mixed-models analysis [36] to test a potential nonlinear factor-specific relationship between factor and DSS by using polynomial, sine wave, and exponential regression models weighing on the sample study size. When the candidate models were nested, we used likelihood ratio tests (F-test) to examine whether the more complex model was a better fit. When comparing two non-nested models, we used the Akaike information criterion [37], which indicates twice the number of parameters of the model minus twice the maximized log-likelihood. The model with lowest Akaike information criterion value was chosen for fitting.

## Results

### Systematic review

The initial screening of the databases for title and abstract yielded 5612 papers, of which 798 papers were chosen for full text reading. A total of 600 articles were excluded for one of the reasons listed in Figure 1. Finally, 198 studies were selected for final analysis with agreement between the two reviewers at 93% (Cohen's kappa = 0·810). Three selected studies separately reported data into two data sets of infants, children, and/or adult groups [38], [39], [40], while one study divided data into three data sets of infants, children, and adult groups [41]; hence a total of 203 data sets were included in the final meta-analysis. Characteristics of the included studies are outlined in Table S2. Most studies were performed in Asia (n = 182); only 12, four, and five studies were from the Caribbean, South American, and French Polynesia, respectively. More studies were prospective (n = 150) than retrospective or not mentioned (n = 53). A total of 88 studies were case-control assessments and 115 were cross-sectional studies. Eight studies included infants, 83 studies enrolled children, 22 studies recruited adults, 54 studies enrolled both infants and children, 18 studies reported both children and adults, 14 studies included all types of subject, and four studies did not provide this information. Clinical diagnosis was used as for dengue infection definition in 14 studies, while serology, PCR, and virus isolation were used for confirmation of all dengue-infected patients in 170 studies. Fourteen studies did not report the criteria for dengue infection. The classification of DSS and DHF varied across studies, but most studies used the WHO 1997 criteria (n = 168), 28 studies used the Nimmannitya criteria [42], while the other seven studies simply classified the diseases as shock versus non-shock group (Table S2).

In terms of the quality of included studies, agreement between the two reviewers was 91% (Cohen's kappa = 0·808). Two studies scored the maximal points (9); the range of total points of included studies was two to nine (Table S2).

A total of 242 factors were reported in at least one study. More than half of them (n = 130) were available in only one study and then were not assessed by meta-analysis, but the association with DSS derived from the original study is shown in the Table S3. There were 112 factors that were reported in two or more studies, and the results of our meta-analysis including the references of included studies for each factor are shown in Table S4. Among 112 factors, 72 factors were investigated in less than eight studies and were not interpreted here because drawing a conclusion is limited when there is very few included studies [26]. There is no clear cut off point for the minimal number of studies included in a meta-analysis to draw a conclusion. Cox *et al* suggest a description of individual studies is better than a meta-analysis [43]. Several studies chose eight as a cut off number for studies included in a meta-analysis to perform a regression analysis [44] and assessment of publication bias [45].

Finally, 40 factors were fully analyzed and interpreted when there were eight or more studies assessing the particular factor. Of these interpreted factors, 23 factors were found to be significantly associated with DSS (Table 1).

**Table 1:** Meta-analysis of the association between significant factors and the risk of DSS.

Variable	Number of study	Total sample size (DSS/DHF)	Heterogeneity		Model	Association with DSS			Egger's 2-tailed bias *p*-value
			*p*-value	*I^2^*		*p*-value	Odds ratio (95% CI)	Largest *p*-value after removing any single study	
Gender (female)#	37	1957/4258	0·063	28	Random	<0·001	1·37(1·17 - 1·60)	<0·001	0·63
Age (year)†	37	2927/6400	<0·001	90	Random	<0·001	0·50(0·36 - 0·70)0·27(0·17-0·42)^a^	<0·001	0·009
Malnutrition#	9	1689/3449	0·37	8	Fixed	0·05	1·19(1·00-1·41)1·37(1·18-1·59)^a^	0·863	0·03
Normal nutrition#	9	1616/3398	0·26	21	Fixed	0·03	0·87(0·77-0·99)	0·20	0·43
Neurological signs#	15	859/1891	<0·001	82	Random	0·003	4·66(1·70 - 12·8)	<0·01	0·42
Vomiting/Nausea#	14	839/1391	0·42	3	Fixed	0·001	1·43(1·15 - 1·78)	<0·01	0·82
Abdominal pain#	17	2340/4986	0·014	48	Random	<0·001	2·26(1·76 - 2·89)	<0·001	0·17
Gastrointestinal bleeding#	18	786/1317	0·52	0	Fixed	<0·001	1·84(1·42 - 2·39)	<0·001	0·58
Hemoconcentration*	38	2847/5214	<0·001	71	Random	<0·001	2·61(2·02 - 3·37)	<0·001	0·54
Pleural effusion*	18	1757/3860	<0·001	77	Random	<0·001	10·4(5·47 - 19·6)15·8(7·95 - 31·6)^a^	<0·001	0·07
Ascites#	12	373/763	<0·001	76	Random	<0·001	5·92(5·42 - 14·5)	<0·001	0·99
Hypoalbuminemia*	13	1662/3461	<0·001	81	Random	<0·001	4·34(2·51 - 7·52)	<0·001	0·34
Hypoproteinemia*	8	178/276	0·021	58	Random	0·009	2·45(1·25- 4·81)	<0·05	0·35
Hepatomegaly*	28	4130/8906	<0·001	84	Random	<0·001	3·10(2·18 - 4·41)	<0·001	0·19
ALT*	26	2772/6281	<0·001	82	Random	<0·001	2·15(1·47 - 3·15)	<0·001	0·21
AST*	26	2772/6281	<0·001	89	Random	<0·001	2·08(1·39 - 3·12)	<0·005	0·13
Thrombocytopenia (Low platelet count)*	47	2801/7172	<0·001	79	Random	<0·001	2·64(1·95 - 3·59)	<0·001	0·15
Prothrombin time*	15	1661/3713	<0·001	68	Random	<0·001	2·83(1·84 - 4·37)	<0·001	0·96
activated partial thromboplastin time (APTT)*	13	1557/3678	<0·001	93	Random	<0·001	6·81(2·83 - 16·4)5·18(2·19 - 12·2)^a^	<0·001	0·017
Fibrinogen level*	9	185/456	<0.001	83	Random	<0.001	0.13(0.05- 0.35)	0.001	0.53
DENV-2#	20	1008/2240	<0·001	62	Random	0·019	1·66(1·09 - 2·55)	0·064	0·91
Primary infection#	37	1251/2696	0·67	0	Fixed	<0·001	0·47(0·37 - 0·60)	<0·001	0·76
Secondary infection#	40	1731/2989	<0·001	57	Random	0·001	1·75(1·26 - 2·42)	<0·005	0·84

Pooled ORs with corresponding 95% CIs of the published results were calculated where more than one study had investigated the marker.

* Factor was presented as both dichotomous (frequency of higher values) and continuous (higher value) variables.

# Factor was presented as a dichotomous (frequency of higher values) variable.

† Factor was presented as a continuous variable.

a adjusted odds ratio calculated after the addition of potential missing studies using the trim and fill method of Duvall and Tweedie.

Hemoconcentration was defined as an increase of hematocrit and presented as both dichotomous (frequency of higher values) and continuous (higher value) variables.

Neurological signs (any signs) were defined as patients had any signs of convulsion, decreased consciousness, drowsiness, and lethargy.

A particular dengue serotype infection was defined as a dichotomous variable versus infection with another DENV serotype (e.g., DENV-2 vs. non-DENV-2). Only studies investigated all four strains were included for the analysis.

### Prevalence of DSS among DHF/DSS

In 80 published studies of cross-sectional design, our pooled results showed that the proportion of DSS among DHF/DSS was 28·5% (95% CI: 24·7 - 32·6) with high heterogeneity (*p*-value for heterogeneity <0·001, *I^2^* = 95). Sub-analysis further demonstrated that DSS prevalence in adults (17·7%; 95% CI: 10·1 - 29·4; in 11 studies recruited only adults) was significantly lower than in children (37·4%; 95% CI: 29·6 - 45·9; in 26 studies recruited only children).

**Figure 2:**
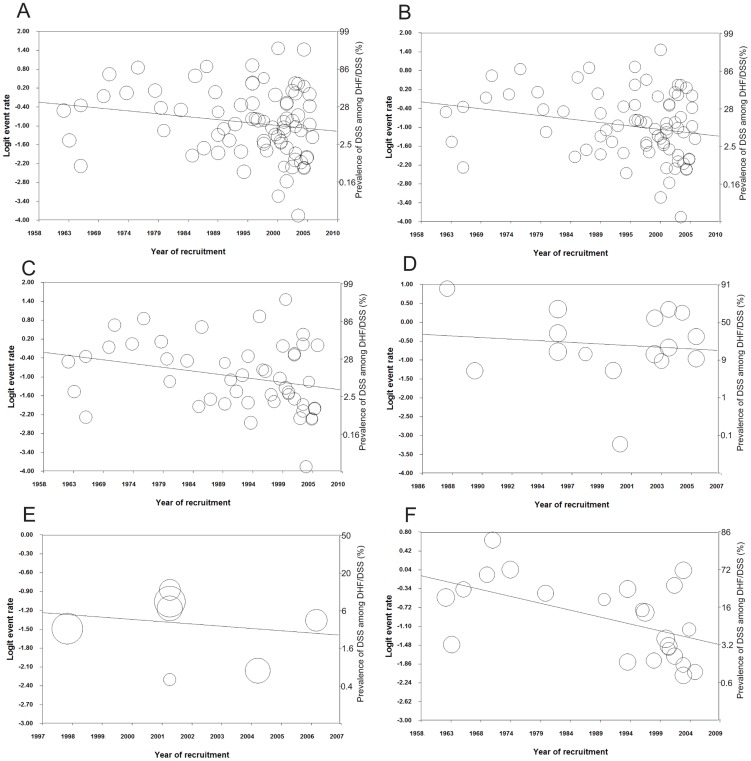
Meta-regression analysis between the proportion of DSS among DHF/DSS cases and year of recruitment. All studies (A). Sub-regression analysis of all studies except three studies in South America (B), 49 studies in Southeast Asia (C), 16 studies in South Asia (D), seven studies in Caribbean countries (E), and 23 studies in Thailand (F). The logit event rate was calculated as follow: logit event rate = ln[event rate/(1 − event rate)]. The Y-axis on the right shows the proportion of DSS patients amongst DHF/DSS patients. Each circle represents a data set in the meta-analysis, and the size of the circle is proportional to study weighting.

Meta-regression analysis showed a trend in the proportion of DSS among DHF/DSS cases that gradually decreased over a period of 40 years, but the trend was not statically significant (*p* = 0·089, Figure 2A). However, after excluding three studies in South America, the decreased trend became significant (*p* = 0·040, Figure 2B). The decreased trend was significant for 49 studies in Southeast Asia (*p* = 0·045, Figure 2C) and particularly steep for 23 studies in Thailand (*p* = 0·004, Figure 2F). The reduced trend was also observed for 16 studies in South Asia but was not statistically significant (*p* = 0·6, Figure 2D), probably due to the small number and shorter duration of studies. The proportion of DSS among DHF/DSS was low in Caribbean countries (pooled prevalence: 19·7%; 95% CI: 15·1 - 25·4; n = 7), explaining the non-significant reduction in prevalence over a period of 25 years (*p* = 0·5, Figure 2E). Other covariates including quality score, sample size, area, country, study design, data collection, different inclusion criteria for DHF, different inclusion criteria for DSS, assignment of the patient (consecutive or random), blinded interpretation of factors, and confirmation of dengue diagnosis had no effect on the pooled prevalence and heterogeneity across studies in Southeast Asia and Thailand, separately. No evidence of publication bias was found using Begg's funnel plot [27] and Egger's regression test [28], [29].

### Gender difference

Meta-analysis of 37 studies for gender difference showed a significant association with DSS (OR: 1·37, 95% CI: 1·17-1·60). Removing any study among selected studies had little effect on the pooled OR (Table 1). Cumulative meta-analysis by repeated meta-analyses each time a new study was added according to sample size demonstrated that this significant association was established without the 17 largest studies. This finding suggested a strong association between female gender and DSS. Significant heterogeneity was found among studies of females; however, removing three studies in the Caribbean area lowered the heterogeneity degree (p value for heterogeneity = 0·075, *I^2^* = 27). Further, removing one study from Colombia made the data homologous (p value for heterogeneity = 0·489, *I^2^* =  = 0), but did not significantly affect the summary effect size.

Meta-regression and sub-analysis for several co-variables including quality of study, year of publication, year of patient recruitment, area/country of the study, study design (all case or case-control), study that included DF patients in the DHF group, data collection (prospective or retrospective), assignment of the patient (consecutive or random), confirmed diagnosis of dengue, different criteria of DSS and DHF, and characteristic of patient population (infant, children, adult) were performed to evaluate the effect of these co-variables on the summarized effect size and heterogeneity (Table S5). The homogeneity was present when pooling 16 studies in children with a positive correlation between female and DSS (OR: 1·23; 95% CI: 1·03-1·51) and five studies in adults with a significant association between female and DSS (OR: 1·32, 95% CI: 0·94-1·87). Moreover, subgroup analysis of 24 prospective studies showed an identical pooled OR 1·36 (95% CI: 1·17-1·59) with a homogenous characteristic (p value for heterogeneity = 0·175, *I^2^* = 21). Other co-variables including quality of study, year of publication, year of patient recruitment, area/country of the study, study design (all case or case-control), study that included DF patients in the DHF group, assignment of the patient (consecutive or random), confirmed diagnosis of dengue, and different criteria of DSS and DHF did not affect the summarized effect size and the heterogeneity. No evidence of publication bias was found for female gender as a factor (Table 1).

### Age factor

Pooled odds ratio showed that age was negatively associated with DSS (OR: 0·50, 95% CI: 0·36 - 0·70). However, pooling all studies gave high heterogeneity and publication bias (*p*<0·001), probably due to large variation of population age in the studies (children/adults). Adding 13 missing studies on the left to enhance the symmetry using the trim and fill method of Duvall and Tweedie (random effect) gave a stronger association with DSS (adjusted OR: 0·27, 95% CI: 0·17-0·42). Because only five and two studies investigated adults and infants, respectively, we could not analyze the age factor in these sub-groups. Pooling 26 studies of children gave a negative association with DSS (OR: 0·67, 95% CI: 0·54 - 0·84).

**Figure 3:**
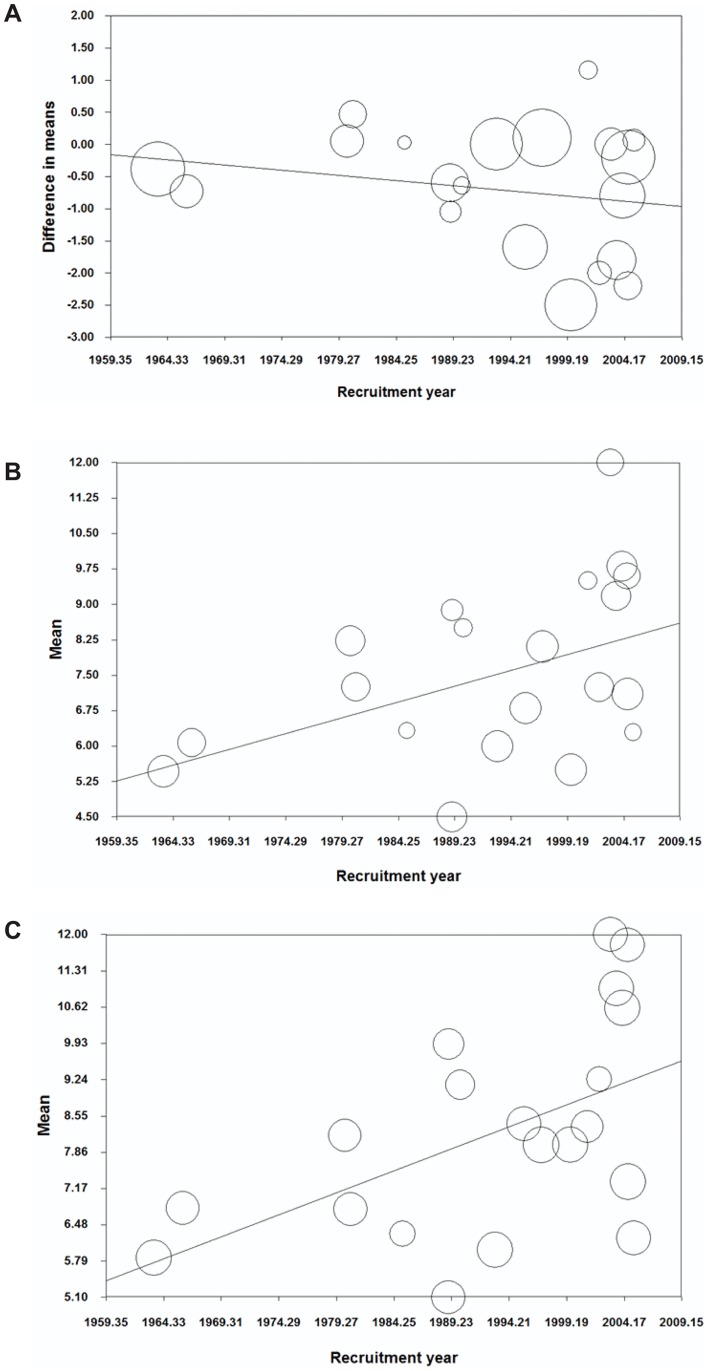
Meta-regression analysis between children's age and DSS association over year of recruitment in South East Asia. (A) Difference in mean age between DSS and DHF groups; (B) Average age of DSS children over year of recruitment in South East Asia; (C) Average age of DHF children over year of recruitment in South East Asia. Each circle represents a data set in the meta-analysis, and the size of the circle is proportional to study weighting.

We further investigated the average age of children in DSS and DHF groups in South East Asia. The difference in average age was slightly wider over a period of 40 years, but the trend was not statistically significant (*p* = 0·37, Figure 3A). There was strong evidence of increasing average age of children in both DSS and DHF groups in this area (*p*<0·05, Figure 3B–C), agreeing with a previous study [46]. The age increase of DHF children (slope = 0·084) was slightly faster than that of DSS (slope = 0·067).

### Nutritional status

**Figure 4:**
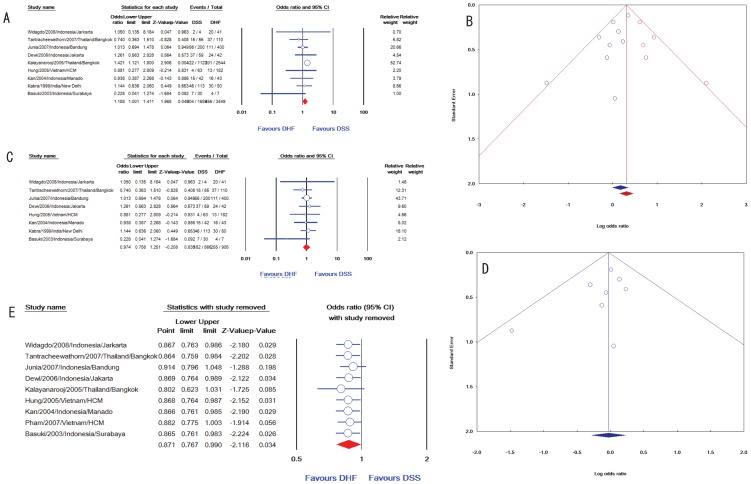
Association of nutritional factors and DSS. (A) Meta-analysis forest plot showing the pooled ORs of malnutrition for association of DSS with 95% CIs using fixed effect models. (B) Funnel plots of publication bias for malnutrition. Each blue circle represents each study in the meta-analysis, forming an asymmetric funnel plot with a pooled log OR (blue rhombus). Five missing studies (red symbols) were added in the right site to make the graph more symmetric and gave an adjusted log OR (red rhombus), which was higher than the original one. (C) Meta-analysis forest plot of malnutrition after removing the largest study. (D) The asymmetric funnel plot of malnutrition became symmetric after removing the largest study. (E) Sensitivity analysis by removing each study showing the pooled association of normal nutrition with DSS without any particular removed study to investigate the effect of each study on the association.

Upon pooling nine studies, malnutrition was positively associated with DSS (OR: 1·19, 95% CI: 1·00-1·41, Figure 4A). No evidence of heterogeneity was found for malnutrition (p = 0·37, *I^2^* = 8), but publication bias was observed by an asymmetric funnel plot (figure 4B) and Egger's test (p = 0·03, Table 1). The definition of malnutrition differed between studies: three studies did not provide definitions [47], [48], [49]; one study used weight-for-height [50], while other five [15], [51], [52], [53], [54] used weight-for-age to assess this factor. Removing any subgroups of no definition and weight-for-height did not affect the association. Furthermore, sub-analysis of five studies using the weight-for-age also gave a positive association with DSS (OR: 1·29, 95% CI: 1·05-1·58) without any evidence of publication bias. However, removal of the largest study [15] eliminated the association of malnutrition and DSS (OR: 0·97, 95% CI: 0·76-1·25, Figure 4C) and publication bias (p = 0·12, Figure 4D).

Normal nutrition was inversely linked with DSS in nine studies (OR: 0·87, 95% CI: 0·77-0·99) without evidence of heterogeneity (p = 0·26, *I^2^* = 21) or publication bias (p = 0·43). No report significantly demonstrated a positive association of this factor with DSS, while one study showed a negative association with DSS [50]. Two studies did not give definition of normal nutrition [47], [49]; two study used weight-for-height [50], [55], while other five [15], [51], [52], [53], [54] used weight-for-age to assess this factor. Removing the subgroup of no definition [47], [49] gave a significant association with DSS (OR: 0·86, 95% CI: 0·76-0·98) with very low heterogeneity (p = 0·42, *I^2^* = 0) and without publication bias (p = 0·83). However, thought sub-analysis of five studies using the weight-for-age still gave an OR <1 but the statistical significant association was lost (OR: 0·92, 95% CI: 0·80-1·05). Furthermore, a sensitivity analysis showed that removing any of three studies of Junia et al [50], Kalayanarooj et al [15], or Pham et al [55] resulted in a loss of statistical association but the ORs were less than one (0·05<p<0·2).

Obesity/overweight was not associated with DSS in eight studies (OR: 1·31, 95% CI: 0·91-1·88). No evidence of heterogeneity or publication bias was found for this factor. One study assessed the obesity/overweight using weight-for-height [50], two studies did not defined the criteria of obesity/overweight [47], [49], while other five studies assessed this factor using weight-for-age. Removing any or all studies by Junia et al [50], Widagdo et al [47], and Basuki et al [49] did not affect the pooled result. No effect of quality score and sample size of included studies on pooled results were observed for three nutritional factors.

### Neurological signs

Neurological signs were defined as any sign of restlessness, irritability, dizziness, drowsiness, stupor, coma, or convulsion. Meta-analysis of 15 studies gave a high pooled OR of 4·66 (95% CI: 1·70-12·8) with high degree of heterogeneity. Subgroup analysis of five studies in Thailand revealed a relative stronger correlation with DSS (OR: 12·7, 95% CI: 6·67-24·3) without evidence of heterogeneity (p = 0·42, *I^2^* = 0). Similarly, summary of seven case-control studies showed a slightly stronger association of encephalopathy with DSS (OR: 5·31, 95% CI: 2·68-10·5) without evidence of heterogeneity (p = 0·25, *I^2^* = 24). Sub-group analysis of nine studies with consecutive or random enrolment showed high heterogeneity (p<0·001, *I^2^* = 89) and non-association with DSS (OR: 3·13, 95% CI: 0·73-13·4). However, removing the study of Kamath et al [56] lowered the heterogeneity (p<0·004, *I^2^* = 67) and made the factor strongly associated with DSS (OR: 5·39, 95% CI: 2·13-13·7). Other co-variables (quality of study, year of publication, year of patient recruitment, study that included DF patients in the DHF group, confirmed diagnosis of dengue, and different criteria of DSS and DHF) did not affect the summarized effect size or heterogeneity. No evidence of publication bias was found for this factor.

### Digestive factors

Among digestive factors, vomiting/nausea and abdominal pain were identified as associated factors for DSS in 14 (OR: 1·43, 95% CI: 1·15-1·78) and 17 (OR: 2·26, 95% CI: 1·76-2·89) studies, respectively. No evidence of heterogeneity was found for vomiting/nausea, while clear evidence of heterogeneity (p = 0·014, *I^2^* = 48) was found for abdominal pain. Cumulative analysis of abdominal pain showed that the eight smallest studies were enough to get a significant association with DSS. Removal of one outlier study [57] eliminated the heterogeneity across studies (p = 0·15, *I^2^* = 27), but did not significantly affect the pooled result (OR: 1·92, 95% CI: 1·72-2·14). Sub-group analysis of abdominal pain studies that used WHO criteria for DSS and DHF also provided a homogenous result, probably due to loss of the outlier study [57]. Meta-regression analysis revealed an increased association with DSS according to publication year (p = 0·049) and quality score (p = 0·004), suggesting a possible under-estimated OR when pooling low-quality and early studies for abdominal pain.

### Bleeding signs

Gastrointestinal bleeding was identified as a positive associated factor for DSS (pooled OR: 1·84, 95% CI: 1·42-2·39), while positive tourniquet test, skin hemorrhages, petechiae, hematuria, and hemoptysis were not associated after pooling more than 10 studies (Table S4). No evidence of heterogeneity was found in 18 studies of gastrointestinal bleeding. No associations between DSS and ecchymoses/purpura and gum or nose bleeding were found in eight and nine studies, respectively, further suggesting that skin and mucosal bleeding were not associated with DSS.

### Plasma leakage signs

All plasma leakage signs (hemoconcentration, pleural effusion, ascites, hypoalbuminemia, and hypoproteinemia), with more than nine studies for each factor, were strongly associated with DSS after pooling relevant studies (Table 1). Significant heterogeneity among sub-group analysis for studies of pleural effusion, ascites, and hepatomegaly remained, and no effect of co-variables on the pooled effect size was found on those factors. Only subgroup analysis for hemoconcentration in South Asia revealed homogeneity across 10 studies without significant change of effect size (OR: 1·77, 95% CI: 1·27, 2·46). After removal of two outliers [58], [59] from 13 primary studies, the summary result of hypoalbuminemia became homogenous without significant changes in the OR. Meta-regression analysis showed that early or low-quality studies significantly overestimated the pooled OR for hypoalbuminemia; however, the summary effect size was not significantly changed after removing the four earliest studies or lowest quality studies. Evidence of publication bias was present for pleural effusion using Egger's test (p = 0·007, Table 1). Adding four missing studies to enhance the symmetry using the trim and fill method of Duvall and Tweedie (random effect) gave a stronger association with DSS (original pooled OR: 10·4, 95% CI: 5·47-19·6; adjusted OR: 15·8, 95% CI: 7·95-31·6).

**Figure 5:**
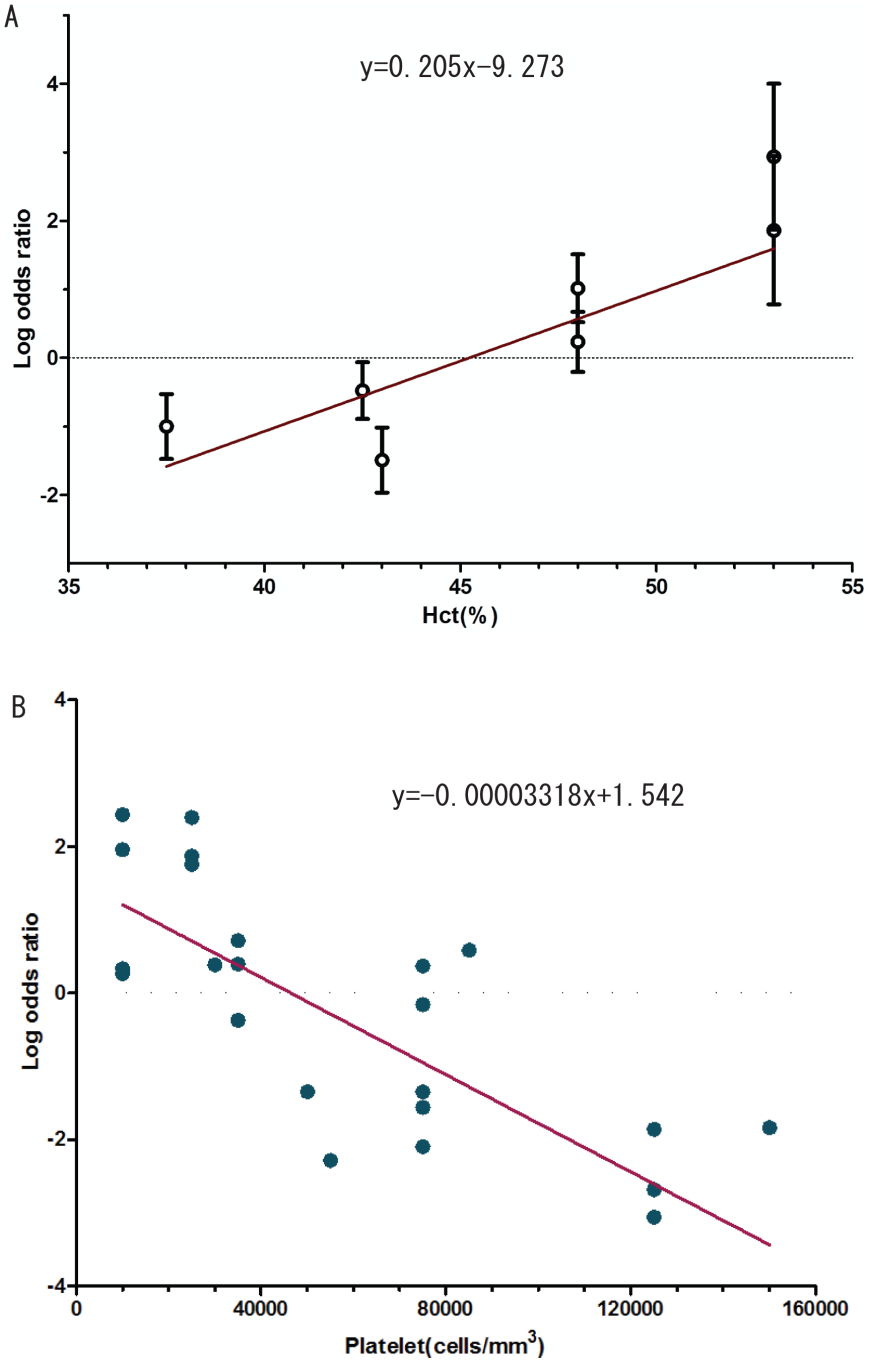
Association between DSS and Hct level (A) or platelet count (B). Each symbol represents each category of Hct or platelet in each included study. Straight lines are the fitted first order polynomial models.

We further plotted the logOR against hematocrit (Hct) categories. The result showed that a first order polynomial model (straight line) was the best fit compared to the second order polynomial, sine wave, and exponential regression models. The fitted trend revealed an increase of logOR at 20·5% (95% CI: 4·65-36·36) for every 1% increment of Hct (Figure 5A).

### Hepatic manifestations

Pooling 26 studies for both alanine transaminase (ALT) and aspartate transaminase (AST) resulted in summary ORs of 2·15 (95%CI: 1·47-3·15, p<0·001) and 2·08 (95%CI: 1·39-3·12, p<0·001), respectively. Cumulative analysis showed that removing at least 11 and 7 of the largest studies was needed to change ALT and AST, respectively, into factors not associated with DSS, indicating a strong association between those two parameters and DSS. There were no effects of co-variables on the pooled OR and heterogeneity for ALT. A subgroup analysis for AST across nine studies in South Asia showed homogeneity but an identical effect size (OR: 2·19, 95% CI: 1·33-3·61). There were a decreasing trend of OR for AST over the recruitment year and publication year of studies; however, after removal of two earliest studies [60], [61], the effect of recruitment year and publication year on OR was lost, suggesting the two early studies overestimated the association with DSS.

Hepatomegaly was strongly associated with DSS after pooling 28 relevant studies (Table 1). Significant heterogeneity among sub-groups remained, and no effect of co-variables on pooled effect size was found. No evidence of publication bias was found.

### Blood cells

A negative association of blood platelet count was demonstrated by pooling 47 studies. The relationship between DSS and blood platelet count is shown in the (Figure 5B). Based on the F-test and the Akaike information criterion values [37], the relationship between the logOR and blood platelet count was best interpreted by a linear equation with a raise of logOR at 33·2% (95% CI: 20·3-46·1) for every 10,000 platelet cells decrement. White blood cells and leukopenia were not associated with DSS in the meta-analysis of 15 and nine studies, respectively (Table S4).

### Coagulators

Meta-analysis of coagulators showed a positive association between DSS and prolonged prothrombin time (PT) and activated partial thromboplastin time (APTT). One study [62] only reported risk ratio and could not be combined with other the 15 studies for both factors. However, this study also suggested a positive association with DSS, agreeing with the pooling effect size of the other 15 studies. Heterogeneity disappeared after removing two outliers regarding PT factor [63], [64] or computing values only in the infant/children group, but the summary OR was unchanged. Subgroup analysis for APTT factor showed homogenous results in children's group. Evidence of publication bias was present using Egger's test (p = 0·017, Table 1). Adding one missing study to enhance the symmetry using the trim and fill method of Duvall and Tweedie gave a similar result (adjusted OR: 5·18, 95% CI: 2·19-12·2). Inverse associations between DSS and fibrinogen level were found in nine studies (Table 1).

### Viral factors

Primary infection was reported as an inverse associated factor in two of 37 studies (Table 1). However, most of the ORs were consistently less than one, and pooling of 37 studies gave a strong negative association with DSS (OR: 0·47, 95% CI: 0·37- 0·60, p<0·0001) without evidence of heterogeneity. Cumulative analysis by repeatedly pooling each time after adding a new study according to sample size showed that an inversely significant association was established without the 28 largest studies, further supporting a strong inverse association between primary infection and DSS. Meta-regression analysis revealed a significant decreased OR of DSS in larger studies (p-value for slope = 0·036), and no effect of other co-variables on the summary effect. Meta-analysis of 40 studies for secondary infection gave a positive association with DSS with high degree of heterogeneity (Table 1). In the sensitivity analysis, the summary OR was not significantly affected by any co-variable and when excluding any study or the six largest studies.

**Figure 6:**
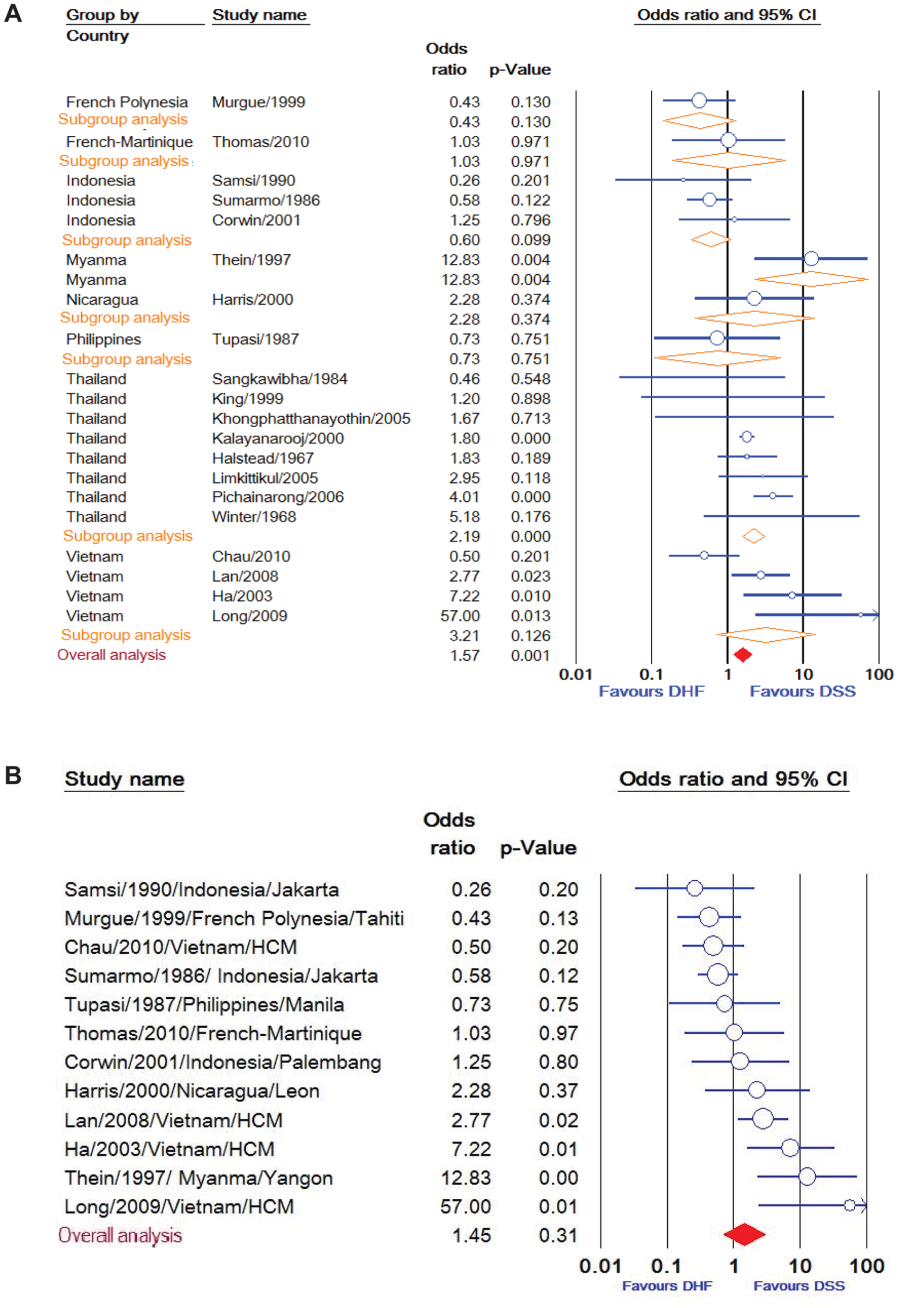
Meta-analysis of DENV-2 serotype including subgroup analysis in each country (A) and all countries beside Thailand (B). (A) Meta-analysis forest plot showing the pooled odd ratio of individual countries (orange symbol) and overall countries (red symbol) for association of DSS with 95% confidence intervals using mixed effect models. (B) The pooled odds ratio in subgroup analysis of all countries excluding Thailand indicated that DENV-2 was not significantly associated with DSS in these areas. The size of the symbol is proportional to study.

Dengue virus serotype 2 (DENV-2) was found to be an associated factor for DSS (OR: 1·66, 95% CI: 1·09 - 2·55), whereas DENV-1, DENV-3, and DENV-4 were not significantly associated with DSS after pooling the whole population. Significant heterogeneity among studies of these serotypes was found. However, subgroup analysis of eight DENV-2 studies in Thailand showed a homogenous result with a slightly higher association with DSS (OR: 1·99, 95% CI: 1·63 - 2·43), but no association between DSS and DENV-2 in other individual countries or in all countries after excluding Thailand (Figure 6).

## Discussion

Our meta-analysis indicated a sustained reduction of DSS/DHF ratio in Southeast Asia, suggesting an improvement in clinical recognition and management over the 40-year period (Figure 3). This improvement may have been due to educational programs and early treatment of rehydration [65]. The upward shift of average age in South East Asia (Figure 3) and previous study [46]), coupled with the lower risk of DSS in older age [4], [66] could be another reason for the reduction of DSS/DHF ratio over this period. It is well known that young children have an increased risk of severe dengue infection [4], [55], probably due to a combination of timing of secondary infection, development of protective immune responses after infection with four strains [67], and increased microvascular fragility in younger children [68]. Our pooled result of 37 studies further strengthened the negative association of age with DSS.

It is thought that normal nutrition is a risk factor of DSS, while malnutrition is a protective factor due to suppressed immune activation in malnourished children [12], [13], [14]. However, both factors had conflicting effects on DSS risk in previous studies [15], and our pooled results of all relevant studies suggested that malnutrition was a positive associated factor for DSS, while normal nutrition helped protect against DSS. However, these associations should be interpreted with caution, because removal of one particular study led to a loss of statistical association of DSS with both factors. Thus, more well-designed prospective studies using both weight-for-height and weight-for-age for nutritional status [69] are required to confirm these results.

The association between being female gender and risk of DSS is not fully understood, but may be explained by gender differences in seeking healthcare as well as physical characteristics [4]. Several manifestations of neurological disorders and liver damage were found to be strongly associated with DSS in our meta-analysis, probably due to the sequelae of shock, systemic inflammation, and direct viral invasion into the organs [70]. As expected, DSS was associated with signs of plasma leakage including hemoconcentration, pleural effusion, ascites, hypoalbuminemia, and hypoproteinemia to varying degrees. Based on the curve fitting for the included studies, the logarithm scale for the OR was raised approximately 20% for every 1% increment of Hct.

Notably, only gastrointestinal bleeding was found to be associated with DSS, while other cutaneous and mucosal bleedings were not. Thrombocytopenia is a well-known marker of dengue severity. The WHO recently suggested that a “rapid drop in platelet count” is a warning sign for dengue severity [3]. Using a value-specific analysis, we demonstrated that the logarithm scale of OR for DSS over DHF was increased 33% for every 10,000 platelet cells decrement. More studies on the association of kinetic platelet count with the risk of dengue severity are recommended to define the “rapid drop in platelet count” and predict the patients who will develop the severity.

Our results further confirm a positive association of DSS with secondary dengue infections, probably related to the role of antibody-dependent enhancement in DSS pathogenesis. We found that DENV-2 was associated with DSS in Thailand, but not in other countries. This could be due to differences in primary/secondary infections, a highly virulent Asian DENV-2 genotype circulating in Thailand [71], ethnic factors, or the small number studies in other countries. Future studies need to analyze the risk of DSS in separate primary or secondary infected patients and simultaneously detect four DENV strains.

One limitation of our study is that the effect of primary/secondary infection, data during epidemics, other underlying diseases, and early treatment could not be assessed because of limited information provided in the included studies. Secondly, the variability in study designs, diagnoses, population selection, and severity definitions [72] could limit the interpretation of our results. In addition, several factors, particularly cytokines and lipid profiles, were found to be associated with DSS, but the number of studies was small [11]. Thus, the results for these factors must be interpreted with caution and more studies are required to validate them. Thirdly, we transformed standardized mean difference to OR based on the assumption that the continuous data had a logistic distribution [21]. Though the method is reportedly reasonable [73], some continuous could have skewed distribution. However, our sensitivity analyses showed that separately pooling studies with category and continuous variables, respectively, did not affect the summarized effect size and the heterogeneity (Table S5). Fourthly, the results of Figure 5 should be interpreted with caution because of ‘ecological fallacy’, which happen when the relationship between risks of DSS with particular factors across studies could be different from that relationship within studies [26]. Another limitation of the study is that we could not investigate the factors in a time-course profile in which a factor may change quickly before and after defervescence, potentially leading to high heterogeneity across studies.

In conclusion, although some factors were found to have contradictory effects, this meta-analysis identified significant associations between 21 factors (including age, female sex, neurological signs, nausea/vomiting, abdominal pain, gastrointestinal bleeding, hemoconcentration, ascites, pleural effusion, hypoalbuminemia, hypoproteinemia, hepatomegaly, levels of alanine transaminase and aspartate transaminase, thrombocytopenia, prothrombin time, activated partial thromboplastin time, fibrinogen level, primary/secondary infection, and DENV-2) and DSS. These results may improve the understanding of the epidemiology, clinical manifestation, and pathogenesis of DSS.

## Supporting Information

Checklist S1 **PRISMA 2009 checklist.**
(DOC)

Method S1 **Format of data extraction.**
(DOC)

Table S1 **Scoring system for quality assessment of selected studies.**
(DOC)

Table S2 **Characteristic of studies included in this meta-analysis.**
(DOC)

Table S3 **Factors that was only investigated in one study or several studies that cannot be extracted.** The association of the factor with DSS was derived from the original study.
(DOC)

Table S4 **Meta-analysis of DSS with factors that were investigated in at least two studies.** Pooled odds ratios (OR) with corresponding 95% confidence intervals (95%CI) of the published results were calculated where more than one study had investigated the factor.
(DOC)

Table S5 **Sensitivity and sub-analysis of co-variables on the effect size and heterogeneity.**
(DOC)
