# Supplementary material for: Factors Associated with Dengue Shock Syndrome: A Systematic Review and Meta-Analysis
Source: PLoS Negl Trop Dis. 2013 Sep 26;7(9):e2412. doi: 10.1371/journal.pntd.0002412 (PMC3784477; doi:10.1371/journal.pntd.0002412)
Supplement: Method S1 — Format of data extraction. (DOC) [file pntd.0002412.s002.doc]

**Method S1. Format of data extraction**

| **Format of data input for factors** |
| --- |
| ***Dichotomous data (number of events)*** |
| - Events and sample size in each group |
| - Non-events and sample size in each group |
| - Events and non-events in each group |
| - Event rate and sample size in each group |
| - Chi-squared and total sample size |
| ***Continuous data*** |
| - Mean, standard deviation (SD), and sample size in each group |
| - Difference in means, common SD, and sample size |
| - Cohen’ s d (standardized by pooled within-groups SD) and sample size |
| - Mean, sample size, and t-value |
| - Difference in means, sample size, and t-value |
| - Sample size and t-value |
| - Mean, sample size, and p-value |
| - Difference in means, sample size, and p-value |
| - Sample size and p-value |

When several types of data or several methods were presented for one particular factor, we extracted all data but used the one with the least significant association (the nearest odds ratio [OR] to one) if that factor was significantly associated with DSS after meta-analysis. Otherwise, data with lowest and highest ORs were pooled separately to get minimal and maximal odd ratios, respectively. When data were available on different days of the disease course, values at day 4, 3, 5, 2, and 6 were favored in that order for analysis, because shock frequently occurs at day 4 and we emphasize the importance of early prediction of DSS.

When the included study reported the DSS or DHF group in two separated groups (such as DHF grade III and IV for DSS, and DHF grade I and II for DHF), a combined mean and standard deviation (SD) were calculated according to respective equations shown below[1]:

**Equation for calculation of mean and standard deviation (SD) from two groups**

***Mean***


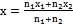
 whereas n_1_, n_2_ are patient number of two group; x_1_, x_2_ are mean of two groups

***SD***


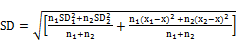
 whereas SD_1_, SD_2_ are SD of two groups

When the included study only reported the mean (or median), range, and the sample size, the mean and SD were estimated according to Hozo et al. [2] as follows:

**Estimation of mean and SD from reported mean/median and range**

***Mean***

-
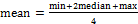
 when sample size ≤25
-
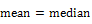
 when sample size >25

***SD***

-
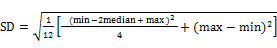
 when sample size ≤25
-
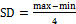
 when 25 <sample size ≤70
-
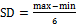
 when 25 <sample size >70

When the published study only reported the mean, the estimated SD was derived from linear regression of log(published SDs) against log(published means) according to van Rijkom *et al* [3]. The published SDs and means were collected from other included studies. Sub-analysis was further performed to investigate the effect of the calculation and estimation on the pooled result.

**References**

1. Goon AM, Gupta MK, Gupta DB (1987) Fundamentals of Statistics. Calcutta, India: The World Press.

2. Hozo SP, Djulbegovic B, Hozo I (2005) Estimating the mean and variance from the median, range, and the size of a sample. BMC Med Res Methodol 5: 13.

3. van Rijkom HM, Truin GJ, van 't Hof MA (1998) A meta-analysis of clinical studies on the caries-inhibiting effect of fluoride gel treatment. Caries Res 32: 83-92.
