# Supplementary material for: Factors Associated with Dengue Shock Syndrome: A Systematic Review and Meta-Analysis
Source: PLoS Negl Trop Dis. 2013 Sep 26;7(9):e2412. doi: 10.1371/journal.pntd.0002412 (PMC3784477; doi:10.1371/journal.pntd.0002412)
Supplement: Table S1 — Scoring system for quality assessment of selected studies. (DOC) [file pntd.0002412.s003.doc]

**Table S1.** **Scoring system for quality assessment of selected studies**

| **Criteria** | **0 point** | **1 point** |
| --- | --- | --- |
| study design | case or no description | all case |
| characteristic of patient population (infant, children, adult) | or no description | full description |
| data collection | retro or no description | prospective |
| assignment of the patient | not consecutive, random, nor description | consecutive or random |
| inclusion criteria | no description | full description |
| exclusion criteria, | no description | full description |
| method quality |  | description and same method for DSS and DHF groups |
| interpretation of factors | not blinded or no description | description of blinded method |
| dengue diagnosis |  | full description |
