# Supplementary material for: Factors Associated with Dengue Shock Syndrome: A Systematic Review and Meta-Analysis
Source: PLoS Negl Trop Dis. 2013 Sep 26;7(9):e2412. doi: 10.1371/journal.pntd.0002412 (PMC3784477; doi:10.1371/journal.pntd.0002412)
Supplement: Table S2 — Characteristic of studies included in this meta-analysis. (DOC) [file pntd.0002412.s004.doc]

**Table S2. Characteristic of studies included in this meta-analysis.**

| **Author/Publication year/Country/City** | **Data year** | **Study design** | **DSS** | **DHF** | **Subject of study** | **Data collection** | **Recruit**  **-ment** | **Diagnosis of DSS** | **Criteria of DSS**  **(WHO 1997)**^h^ | **Quality score** |
| --- | --- | --- | --- | --- | --- | --- | --- | --- | --- | --- |
|  |  |  | (n) | (n) | I: Infant |  |  |  |  |  |
|  |  |  |  |  | C: Children |  |  |  |  |  |
|  |  |  |  |  | A; Adult |  |  |  |  |  |
|  |  |  |  |  | Range, mean± |  |  |  |  |  |
| **Agarwal/1998/India/New Delhi&Lucknow [1]** | 1996 | case^g^ | 31 | 113 | ND | prosp^h^ | ND | Confirmed ELISA, virus | Yes | 4 |
| **Agarwal/1999/India/Lucknow&New Delhi [2]** | 1996 | ND | 26 | 35 | I,C,A (8m-55y) | prosp | ND | Confirmed ELISA, Virus | Yes | 5 |
| **Aggarwal/1998/India/New Delhi [3]** | 1996 | cross^g^ | 42 | 92 | I,C (0-12y) | retro | conse^k^ | ND | Yes | 5 |
| **Ahmed/2001/Bangladesh/Chittagong [4]** | 2000 | cross | 10 | 36 | C (2-12y) | prosp | conse | Confirmed ELISA | Yes | 8 |
| **Arguelles/1987/Cuba/La Habana [5]** | 1981 | ND | 40 | 75 | C (2-16y) | prosp | conse | Confirmed HI | Yes | 6 |
| **Avirutnan/2006/Thailand/Khon Khan [6]** | 2001-2003 | cross | 16 | 88 | C (1-15y) | prosp | conse | Confirmed Virus ELISA +PCR | Yes | 8 |
| **Azad/2006/Bangladesh/Dhaka [7]** | 2000-2001 | cross | 5 | 127 | C,A (10·67 ± 2·33; 27·59 ± 1·18) | prosp | conse | Confirmed ELISA | Yes | 7 |
| **Balmaseda/2005a/Nicaragua/Managua&Leon[8]** | 1999-2002 | cross | 2 | 20 | A (29·5±12·8; 28·9±12·7) | prosp | conse | Confirmed ELISA,PCR, Virus | Yes | 7 |
| **Balmaseda/2005c/Nicaragua/Managua&Leon[8]** | 1999-2002 | cross | 64 | 185 | C (1-14y) | prosp | conse | Confirmed ELISA,PCR, Virus | Yes | 7 |
| **Balmaseda/2005i/Nicaragua/Managua &Leon[8]** | 1999-2002 | cross | 10 | 24 | I (0–11m) | prosp | conse | Confirmed ELISA,PCR, Virus | Yes | 7 |
| **Basu/2007/India/Kolkata [9]** | 2005 | cross | 9 | 7 | I,C (0-12y) | prosp | conse | Confirmed ELISA (MAC) | Yes | 7 |
| **Basuki/2003/Indonesia/Surabaya [10]** | 2000-2001 | cross | 30 | 7 | C (2-13y) | prosp | conse | Non-confirmed and confirmed cases (ELISA) | Yes | 6 |
| **Bethell/1998/Vietnam/Dongnai [11]** | 1993 | cross | 71 | 184 | C (6.6,·0–7.0; 6. 5, 0–8.0) | prosp | conse | Confirmed ELISA | Yes | 8 |
| **Bethell/2001/Vietnam/HCM [12]** | 1994-1995 | case | 19 | 16 | C (5-15y) | prosp | conse | Confirmed ELISA | Yes | 8 |
| **Bhattacharjee/1993/India/Calcutta [13]** | 1990 | cross | 11 | 40 | C,A (ND) | ND | conse | Non-confirmed and confirmed cases (Virus culture, HI, MAC-ELISA, CF) | Yes | 6 |
| **Bokisch/1973/Thailand/Bangkok [14]** | 1971 | case | 12 | 5 | I,C (ND) | prosp | ND | confirmed Virus culture, HI | Nimmannitya^i^ | 5 |
| **Boonpucknavig/1976/Thailand/Bangkok [15]** | ND | ND | 20 | 42 | C (ND) | prosp | ND | Confirmed HI | Nimmannitya | 4 |
| **Boonpucknavig/1978/Thailand/Bangkok [16]** | ND | ND | 26 | 65 | C (1-12y) | prosp | ND | Confirmed HI | Nimmannitya | 5 |
| **Butthep/2006/Thailand/Bangkok [17]** | ND | cross | 14 | 64 | C (4-16y) | prosp | conse | Confirmed ELISA | Yes | 7 |
| **Capeding/1997/Philippines/Manila [18]** | 1986-1994 | cross | 22 | 142 | I,C,A (8m-52y) | retro | conse | Confirmed HI, Virus | Yes | 5 |
| **Capeding/1997/Philippines/Manila [19]** | 1992-1993 | cross | 5 | 17 | C,A (ND) | prosp | ND | Confirmed HI, Virus | Yes | 6 |
| **Chacko/2008/India/Chennai [20]** | 2005 | cross | 34 | 39^a^ | I,C (1m-18y) | prosp | conse | Confirmed ELISA | Yes | 8 |
| **Chairulfatah/1995/Indonesia/Bandung [21]** | 1991-1993 | cross | 24 | 104 | C (<15y) | prosp | conse | Confirmed HI | Yes | 7 |
| **Chairulfatah/2001/Indonesia/Bandung [22]** | 1994-1995 | cross | 81 | 569 | C,A (ND) | retro | conse | Non-confirmed | Yes | 6 |
| **Chairulfatah/2003/Indonesia/Bandung [23]** | 1994-1995 | cross | 102 | 1198 | C,A (ND) | retro | conse | Non-confirmed | Yes | 6 |
| **Chaturvedi/1999/India/New Delhi [24]** | ND | case | 42 | 164 | I,C,A (8m-57y) | prosp | conse | Confirmed ELISA, virus | Yes | 6 |
| **Chaturvedi/1999/India/New Delhi,Lucknow [25]** | 1996 | cross | 35 | 47 | I,C,A (8m-55y) | prosp | conse | Confirmed ELISA, virus | Yes | 7 |
| **Chaturvedi/2001/India/Lucknow&NewDelhi[26]** | 1996 | case | 26 | 60 | ND | prosp | ND | Confirmed ELISA, Virus | Yes | 4 |
| **Chau/2008/Vietnam/HCM [27]** | 2004-2006 | cross | 6 | 63 | I (<18m) | prosp | conse | Confirmed PCR+ELISA | Yes | 7 |
| **Chau/2010/Vietnam/HCM [28]** | 2004-2007 | cross | 19 | 141 | I (<18m) | prosp | conse | Confirmed ELISA | Yes | 7 |
| **Chaudhary/2006/India/Lucknow [29]** | 2003 | cross | 10 | 80 | I,C,A (9m-72y) | retro | conse | Confirmed ELISA | Yes | 6 |
| **Chhina/2008/India/Punjab [30]** | 2006 | cross | 11 | 29 | A (15-80y) | prosp | conse | Confirmed ELISA | Yes | 8 |
| **Chongsrisawat/2009/Thailand/Bangkok [31]** | 1997-2004 | cross | 12 | 4^b^ | C (<15y) | retro | conse | Confirmed ELISA | Yes | 6 |
| **Chuansumrit/2010/Thailand/Bangkok [32]** | ND | cross | 10 | 63 | C (5-15y) | prosp | conse | Confirmed ELISA | Yes | 7 |
| **Chungue/1994/French Polynesia/Tahiti [33]** | ND | ND | 48 | 40^a^ | I,C (0-12y) | prosp | ND | Confirmed HI, ELISA, Virus | Yes | 5 |
| **Chunhakan/2009/Thailand/Bangkok [34]** | ND | cross | 19 | 79 | C (2-19y) | prosp | conse | Confirmed ELISA+Virus | Yes | 8 |
| **Churdboonchart/1983/Thailand/Bangkok [35]** | ND | ND | 39 | 15 | C (2-14y) | prosp | ND | Confirmed HI | Yes | 5 |
| **Cohen/1966/Thailand/Bangkok [36]** | 1964 | cross | 23 | 100 | I,C (5m-17y) | prosp | conse | Confirmed HI, CF | Shock vs non-shock | 7 |
| **Convers/2001/Colombia/Bucaramanga [37]** | 1994-1998 | cross | 281 | 197 | I,C,A (0·3-97y) | ND | conse | Confirmed ELISA | Yes | 6 |
| **Corwin/2001/Indonesia/Palembang [38]** | 1998 | cross | 12 | 58 | C,A (1-78y) | retro | conse | Confirmed ELISA, PCR, Virus | Yes | 6 |
| **Devignot/2010/Cambodia/Kampong Cham [39]** | 2007 | case | 19 | 13 | C (1-15y) | prosp | ND | Confirmed ELISA, PCR, Virus | Yes | 6 |
| **Dewi/2006/Indonesia/Jakarta [40]** | 2003-2004 | cross | 59 | 42 | I,C (5m-15y) | retro | conse | Confirmed ELISA | Yes | 6 |
| **Edelman/1975/Thailand/Bangkok [41]** | 1972 | ND | 11 | 7 | C (ND) | prosp | ND | Confirmed HI | Nimmannitya | 5 |
| **Eram/1979/ Indonesia/Yogyakarta [42]** | 1976 | cross | 35 | 15 | I,C (0-15+y) | prosp | conse | Confirmed Virus culture | Nimmannitya | 7 |
| **Funahara/1985/Indonesia/Jakarta [43]** | 1985 | ND | 3 | 7 | I,C (8m-12y) | prosp | ND | Confirmed serological | Yes | 5 |
| **Glaziou/1992/French Polynesia/Tahiti_C [44]** | 1989-1990 | ND | 34 | 84^a^ | C (1-15y) | ND | ND | Confirmed Virus iso, ELISA | Yes | 3 |
| **Glaziou/1992/French Polynesia/Tahiti_I[44]** | 1989-1990 | ND | 27 | 33^a^ | I (1-12m) | ND | ND | Confirmed Virus iso, ELISA | Yes | 3 |
| **Gonza´lez/2005/Havana/Cuba [45]** | 2001-2002 | cross | 18 | 58 | A (16-64y) | retro | conse | Confirmed ELISA | Yes | 6 |
| **Gorp/2002/Indonesia/Semarang [46]** | 1996 | cross | 50 | 20 | C (6·5± 2·8y) | prosp | conse | Confirmed ELISA | Yes | 8 |
| **Gubler/1979/ Indonesia/Yogyakarta [47]** | 1976 | cross | 19 | 8 | I,C (0-15+y) | prosp | conse | Confirmed Virus culture | Nimmannitya | 7 |
| **Gupta/2000/India/New Delhi [48]** | 1998 | cross | 3 | 7 | C (ND) | prosp | conse | Confirmed HI | Yes | 7 |
| **Guzman/1984/Cuba/La Habana [49]** | 1981 | ND | 21 | 82 | I,C,A (0->25y) | ND | ND | Confirmed HI | Yes | 4 |
| **Ha/2003/Vietnam/HCM [50]** | 2001 | ND | 16 | 24 | C,A (<4-71y) | retro | ND | Confirmed ELISA | Yes | 2 |
| **Halstead/1967/Thailand/Bangkok [51]** | 1962-1964 | cross | 196 | 329 | C (1-16y) | ND | ND | ND | Nimmannitya | 5 |
| **Halstead/1970/Thailand/Bangkok [52]** | 1962-1965 | ND | 190 | 262 | I. C (0-14y) | prosp | ND | Confirmed serology | Nimmannitya | 5 |
| **Hanafusa/2008a/Thailand/Rayong [53]** | 2004-2005 | case | 18 | 76 | A(25·47±10·46y) | retro | ND | Non-confirmed | Yes | 5 |
| **Hanafusa/2008c/Thailand/Rayong [53]** | 2004-2005 | case | 19 | 49 | C (9·31±3·48y) | retro | ND | Non-confirmed | Yes | 5 |
| **Harris/2000/Nicaragua/Leon [54]** | 1998 | cross | 60 | 267 | I,C,A (0·1-84y) | prosp | conse | Confirmed ELISA, PCR | Yes | 8 |
| **Hober/1993/French Polynesia/Tahiti [55]** | 1989-1990 | ND | 14 | 17 | I,C,A (3m-66y) | ND | ND | Confirmed ELISA, Virus | Nimmannitya | 4 |
| **Homchampa/1988/Thailand/Bangkok [56]** | 1986-1987 | ND | 18 | 18 | C (4-12y) | prosp | ND | Confirmed HI | Nimmannitya | 4 |
| **Hongsiriwon/2002/Thailand/Chon Buri [57]** | 1995-1998 | cross | 6 | 13 | I (3-12m) | retro | conse | Confirmed ELISA, HI | Yes | 7 |
| **Honsawek/2007/Thailand/Bangkok [58]** | 2004-2005 | ND | 10 | 14 | C (11·2±2·5; 8·9±1·8) | prosp | ND | Confirmed ELISA | Yes | 5 |
| **Hoti/2006/India/Pondicherry [59]** | 2003 | ND | 8 | 14 | I,C,A (0->45y) | prosp | ND | Confirmed ELISA | Yes | 5 |
| **Hung/2004/Vietnam/HCM [60]** | 1998-2002 | cross | 22 | 85 | I (1-11m) | prosp | conse | Confirmed ELISA | Yes | 7 |
| **Hung/2005/Vietnam/HCM [61]** | 1997-2002 | cross | 63 | 182 | I (<12m) | prosp | conse | Confirmed ELISA, HI | Yes | 7 |
| **Hung/2006/Vietnam/HCM [62]** | 1997-2002 | cross | 63 | 145 | I (1-11m) | prosp | conse | Confirmed ELISA | Yes | 7 |
| **Ibrahima/2007/Malaysia [63]** | 2001-2002 | case | 4 | 82 | A (12-83y) | prosp | ND | Confirmed ELISA | Yes | 5 |
| **Isarangkura/1987/Thailand/Bangkok [64]** | ND | ND | 16 | 20 | I,C (0·5-14y) | prosp | ND | Confirmed Serological | Nimmannitya | 4 |
| **Itha/2005/India/Lucknow [65]** | 2003 | cross | 7 | 15 | C,A (7-65y) | prosp | conse | Non-confirmed and confirmed cases ( ELISA) | Yes | 7 |
| **Jimenez/1984/Cuba/La Habana [66]** | ND | ND | 10 | 12 | C,A (ND) | prosp | ND | ND | Yes | 4 |
| **Juffrie/2000/Indonesia/Yogyakarta [67]** | 1995-1996 | ND | 29 | 42 | C (1-14y) | prosp | ND | Confirmed ELISA | Yes | 5 |
| **Juffrie/2001/Indonesia/Yogyakarta [68]** | 1995-1996 | ND | 29 | 42 | C (1-14y) | prosp | ND | Confirmed ELISA | Yes | 5 |
| **Juffrie/2002/Indonesia/Yogyakarta [69]** | 1995-1996 | ND | 29 | 39 | C (8·01±9·14) | prosp | ND | Confirmed ELISA | Yes | 5 |
| **Junia/2007/Indonesia/Bandung [70]** | 2004-2005 | case | 200 | 400 | C (7·1±3·2; 7·3±3·5) | ND | ND | Non-confirmed included | Yes | 5 |
| **Kabilan/2005/India/Chennai [71]** | 2001-2002 | case | 34 | 16 | I,C (0-15y) | prosp | ND | Confirmed ELISA | Yes | 5 |
| **Kabra/1998/India/New Delhi [72]** | 1996 | cross | 22 | 21 | I,C (0·5-12y) | prosp | conse | Confirmed ELISA, IIFA, virus | Yes | 8 |
| **Kabra/1999/India/New Delhi [73]** | 1996 | cross | 113 | 80 | I,C (4m-13y) | prosp | conse | Confirmed ELISA, IIFA, virus | Yes | 7 |
| **Kalayanarooj/1989/Thailand/Bangkok [74]** | ND | ND | 89 | 91 | I,C (0·5-18y) | prosp | ND | ND | Yes | 3 |
| **Kalayanarooj/1999/Thailand/Bangkok& Kampang Pet [75]** | 1994-1997 | cross | 22 | 120 | I,C (6m-15y) | prosp | conse | Confirmed ELISA, virus | Yes | 8 |
| **Kalayanarooj/2000/Thailand/Bangkok [76]** | 1995-1999 | cross | 538 | 1378 | C (ND) | retro | conse | Confirmed PCR, Virus | Yes | 6 |
| **Kalayanarooj/2002/Thailand/Bangkok [77]** | 1995-1999 | cross | 1118 | 2520 | I,C (8·1; 8) | retro | conse | Confirmed ELISA, virus | Yes | 6 |
| **Kalayanarooj/2003/Thailand/Bangkok [78]** | 1995-1999 | cross | 1137 | 2585 | I,C (1m-18y) | retro | conse | Confirmed ELISA, HI, PCR, Virus | Yes | 6 |
| **Kalayanarooj/2005/Thailand/Bangkok [79]** | 1995-1999 | cross | 1123 | 2544 | C (7·9±3·8y) | retro | conse | Confirmed ELISA, HI | Yes | 6 |
| **Kalayanarooj/2007/Thailand/Bangkok [80]** | 1994 | cross | 21 | 130 | C (ND) | prosp | conse | Confirmed Virus ELISA | Yes | 7 |
| **Kamath/2006/India/Chennai [81]** | 2001-2003 | cross | 73 | 36^c^ | I,C (0-15y) | retro | conse | Confirmed ELISA | Yes | 5 |
| **Kan/2004/Indonesia/Manado [82]** | 2000 | cross | 42 | 43 | C (2-12y) | prosp | conse | Confirmed ELISA | Yes | 8 |
| **Kasim/1991/Indonesia/Jakarta [83]** | 1988 | ND | 40 | 45 | C (ND) | prosp | ND | Non-confirmed included (HI) | Nimmannitya | 3 |
| **Khin/1993/Malaysia/Kuala Lumpur [84]** | 1991 | cross | 7 | 21 | A (12-61y) | prosp | conse | Non-confirmed and confirmed cases (Virus culture, HI, ELISA) | Yes | 7 |
| **Khongphatthanayothin/2007/Thailand/Bangkok[85]** | 2002-2005 | cross | 25 | 36 | C (5-15yr) | prosp | conse | Confirmed PCR+ELISA | Yes | 9 |
| **Khongphatthanayothin/2005/Thailand/Bangkok[86]** | 2003-2004 | cross | 11 | 14 | C (5-15y) | prosp | conse | Confirmed PCR, ELISA | Yes | 8 |
| **King/1999/Thailand/Bangkok [87]** | 1990 | cross | 4 | 7 | C (3-15y) | prosp | conse | Confirmed ELISA, Virus | Yes | 7 |
| **Kittigul(A)/2007/Thailand/Petchabun [88]** | 2003-2004 | cross | 7 | 46 | A (15-50y) | prosp | conse | Confirmed ELISA | Yes | 7 |
| **Kittigul(C)/2007/Thailand/Petchabun [88]** | 2003-2004 | cross | 24 | 194 | I,C(0·8–14y) | prosp | conse | Confirmed ELISA | Yes | 7 |
| **Kittigul/1997/Thailand/Khon Kaen [89]** | 1994 | cross | 36 | 51 | C (<15y) | prosp | conse | Confirmed HI | Yes | 7 |
| **Kittigul/2000/Thailand/Anthong [90]** | 1997 | ND | 16 | 46 | C (<15y) | prosp | ND | Confirmed HI | Yes | 5 |
| **Kittigul/2003/Thailand/Anthong [91]** | 1998-1999 | cross | 9 | 54 | I,C (0-15y) | prosp | conse | Confirmed ELISA, HI, Virus | Yes | 7 |
| **Koraka/2001/Indonesia/Yogyakarta&Semarang[92]** | 1995-1996 | ND | 29 | 15 | I,C (7m-14y) | prosp | ND | Confirmed ELISA | Yes | 5 |
| **Koraka/2003/Indonesia/Semarang&Yogyakarta[93]** | 1995-1996 | ND | 67 | 30 | I,C (7m-14y) | prosp | ND | Confirmed Serology | Yes | 5 |
| **Koraka/2004/Indonesia/Semarang&Yogyakarta[94]** | 1995-1996 | ND | 67 | 30 | I,C (7m-14y) | prosp | ND | Confirmed ELISA | Yes | 6 |
| **Koraka/2010/Indonesia/Semarang [95]** | 2001-2003 | cross | 11 | 15 | C (3-14y) | prosp | conse | Confirmed PCR | Yes | 8 |
| **Krishnamurti/2001/Thailand/Bangkok [96]** | 1997-1998 | cross | 9 | 38 | C (2-15y) | prosp | conse | Confirmed ELISA, PCR | Yes | 9 |
| **de Kruif/2008/Indonesia/Semarang [97]** | 2002-2003 | cross | 20 | 36^a^ | C (3-14) | prosp | conse | Confirmed ELISA+Virus | Yes | 7 |
| **Kurane/1991/Thailand/Bangkok [98]** | 1987-1988 | case | 8 | 11 | C (4-14y) | prosp | conse | Confirmed ELISA, HI, Virus | Yes | 8 |
| **Kurane/1993/Thailand/Bangkok [99]** | 1987-1988 | case | 6 | 8 | C (5-14y) | prosp | random | Confirmed HI, Virus | Yes | 6 |
| **Lan/2008/Vietnam/HCM [100]** | 2002-2005 | case | 418 | 211 | I,C (6m-15y) | prosp | conse | Confirmed ELISA | Yes | 6 |
| **Lee/2005/Taiwan/Kaohsiung [101]** | 2002 | cross | 6 | 94 | A (22-88y) | prosp | conse | Confirmed ELISA,PCR | Yes | 8 |
| **Lee/2006/Vietnam/HCM [102]** | ND | case | 10 | 30 | C (8·2±2·9; 10·4±3·2) | ND | ND | ND | Yes | 2 |
| **Lee/2007/Taiwan/Kaohsiung [103]** | 2002 | cross | 3 | 68^d^ | A (18-76y) | prosp | conse | Confirmed serological | Yes | 7 |
| **Lee/2008/Vietnam-Taiwan/HCM&Tainan [104]** | 2002 | case | 5 | 15 | C (5-15y) | prosp | ND | ND | Yes | 5 |
| **Lee/2009/Taiwan/Kaosiung Hsien [105]** | 2002 | cross | 18 | 286 | A (19-88) | retro | conse | Confirmed PCR+ELISA | Yes | 6 |
| **Limkittikul/2005/Thailand/Nongkhai, Nakhon Phanom&Mukdahan [106]** | 2002 | cross | 19 | 25 | C,A (3-30y) | retro | conse | Confirmed PCR | Yes | 6 |
| **Lin/2001/Taiwan/Tainan [107]** | 1998-1999 | case | 3 | 6^a^ | C,A (7-57y) | prosp | ND | Confirmed ELISA, PCR | Yes | 5 |
| **Liu/2002/Taiwan/Tainan [108]** | 1998 | cross | 5 | 3 | A (13-67y) | prosp | conse | Confirmed ELISA,PCR, Virus | Yes | 7 |
| **Loke/2010/Nicaragua/Managua [109]** | 2003-2004 | case | 8 | 6 | I,C (10m-14y) | prosp | conse | Confirmed PCR+ELISA+Virus | Yes | 6 |
| **Long/2009/Vietnam/HCM [110]** | 2005 | case | 9 | 9^a^ | C (9-14y) | prosp | ND | Confirmed PCR+ELISA | Yes | 5 |
| **Lumpaopong/2010/Thailand/Bangkok [111]** | 2004-2007 | cross | 9 | 68 | I,C (0-18y) | retro | conse | Confirmed Serology | Yes | 7 |
| **Mairuhu/2005a/Indonesia/Semarang [112]** | 1996; 2001-2003 | case | 25 | 19^a^ | C (2-14y) | retro | conse | Confirmed ELISA | Yes | 5 |
| **Mairuhu/2005b/Indonesia/Semarang [113]** | 1996-97; 2001-03 | case | 99 | 75 | C (3-14y) | prosp | conse | Confirmed ELISA, PCR | Yes | 6 |
| **Malavige/2006/Seri Lanka/Colombo [114]** | 2004 | cross | 29 | 57 | I,C (1m-12y) | prosp | conse | Confirmed ELISA | Yes | 7 |
| **Manaloto/1987/Philippines/Manila [115]** | 1983-1984 | cross | 14 | 63 | C (1-18y) | ND | conse | Confirmed HI, Virus | Yes | 6 |
| **Mekmullica/2005/Thailand/Bangkok [116]** | 1999-2000 | cross | 6 | 43^e^ | I,C (0-15y) | prosp | conse | Confirmed Serology | Yes | 8 |
| **Mitrakul/1987/Thailand/Bangkok [117]** | ND | ND | 33 | 95 | C (ND) | prosp | ND | ND | Yes | 2 |
| **Mohan/2000/India/New Delhi [118]** | 1996 | cross | 8 | 16 | I,C (2m-12y) | prosp | conse | Confirmed HI | Yes | 8 |
| **Mukerjee/1997/Thailand/Bangkok [119]** | 1993-1995 | case | 27 | 100 | C (ND) | retro | Random | Confirmed ELISA | Yes | 4 |
| **Murgue/1999/French Polynesia/Tahiti [120]** | 1989-1997 | cross | 33 | 31 | I,C (1m-18y) | retro | conse | Confirmed ELISA, PCR, virus | Yes | 6 |
| **Mustafa/2001/India/Lucknow [121]** | ND | case | 26 | 34 | ND | prosp | ND | Confirmed ELISA, Virus | Yes | 4 |
| **Myo-Khin/1995/Myanmar/Yangon [122]** | 1989 | case | 20 | 21 | C (7·1 ±1·9; 6 55 ±2·35) | prosp | conse | Confirmed Virus | Yes | 6 |
| **Narayanan/2003/India/Chennai [123]** | 2001 | cross | 13 | 46^a^ | C(7m-12y) | prosp | conse | Confirmed ELISA | Shock vs non-shock | 8 |
| **Nelson/1965/Thailand/Bangkok [124]** | 1960-1964 | ND | 15 | 55 | I, C (ND) | retro | ND | Confirmed HI | Shock vs non-shock | 4 |
| **Nguyen/1997/Vietnam/HCM [125]** | 1995 | ND | 31 | 14 | C (ND) | ND | ND | Confirmed ELISA (MAC), HI, Virus | Yes | 3 |
| **Nimmannitya/1987/Thailand/Bangkok [126]** | ND | ND | 95 | 47 | C (ND) | prosp | NDacute | ND | Nimmannitya | 2 |
| **Nishioka/1974/Thailand/Bangkok [127]** | ND | case | 10 | 4 | C (4-11y) | prosp | ND | Confirmed HI, CF | Shock vs non-shock | 5 |
| **Ooi/2008/Malaysia/Kuala Lumpur [128]** | 2004 | cross | 9 | 427 | A (12-72y) | retro | conse | Non-confirmed included (HI) | Yes | 6 |
| **Pacsa/2000/India/Lucknow& New Delhi [129]** | 1996 | ND | 26 | 34 | ND | prosp | ND | Confirmed ELISA, Virus | Yes | 4 |
| **Pancharoen/2001/Thailand/Bangkok [130]** | 1988-1995 | Case | 80 | 136 | I,C (0-14y) | retro | ND | Confirmed ELISA, HI | Yes | 4 |
| **Pancharoen/2002/Thailand/Bangkok [131]** | ND | cross | 57 | 27 | I,C (0-15y) | prosp | conse | Confirmed HI | Yes | 8 |
| **Pham/2007/Vietnam/HCM [132]** | 2005 | case | 40 | 40 | C (1-15y) | prosp | Random | Confirmed ELISA | Yes | 7 |
| **Pichainarong/2006/Thailand/Bangkok [133]** | 2002-2003 | case | 105 | 105 | I,C (0-14y) | prosp | random | ND | Yes | 6 |
| **Pongpanich/1973/Thailand/Bangkok [134]** | 1969-1971 | cross | 16 | 17 | C (3·5-12y) | prosp | conse | Confirmed HI | Nimmannitya | 7 |
| **Pongtanakul /2005/Thailand/Bangkok [135]** | 1977-2001 | cross | 8 | 12^f^ | C (2-16y) | retro | conse | Confirmed ELISA | Yes | 6 |
| **Potts/2010/Thailand/Bangkok&KamphaengPhet[136]** | 1994-1997;1999- 2000; 2004-07 | cross | 37 | 171 | I,C (6m-15y) | prosp | conse | Confirmed ELISA, HI, PCR, Virus | Yes | 7 |
| **Preeyasombat/1990/Thailand/Bangkok [137]** | ND | ND | 32 | 28 | C (2y-14y) | prosp | ND | Confirmed Serological | Yes | 6 |
| **Preeyasombat/1999/Thailand/Bangkok [138]** | ND | ND | 33 | 54 | C (2y-14y) | prosp | ND | Confirmed Serological | Yes | 5 |
| **Pushpa/1998/India/Chennai [139]** | 1989-1990 | ND | 20 | 29 | I,C (>6m) | ND | ND | Confirmed ELISA | Yes | 4 |
| **Puspanjono/2007/Indonesia/Jakarta [140]** | 2006 | cross | 30 | 30 | I,C (0-18y) | retro | conse | ND | Yes | 5 |
| **Raghupathy/1998/India/Lucknow&New Delhi [141]** | 1996 | ND | 34 | 39 | C,A (8m-55y) | prosp | ND | Confirmed ELISA, virus | Yes | 5 |
| **Rajendra/2008/India/Pondiacherry [142]** | 2003 | ND | 25 | 32 | A (15-66y) | prosp | ND | Confirmed ELISA | Yes | 5 |
| **Ratageri/2005/India/Hubli [143]** | 2003-2004 | cross | 5 | 14 | I,C (ND) | retro | conse | Confirmed MAC ELISA | Yes | 6 |
| **Ray/1999/India/New Delhi [144]** | 1996 | case | 10 | 42 | I,C (1m-12y) | prosp | random | ND | Yes | 5 |
| **Rivera/2008/Honduras/Tegucigalpa [145]** | 2004-2005 | cross | 15 | 130 | I,C (0-15y) | prosp | conse | Confirmed ELISA | Yes | 7 |
| **Rongrungruang/2001/Thailand/Bangkok [146]** | 1990-1997 | ND | 25 | 159 | A (12-57y) | retro | ND | Non-confirmed and some by HI | Yes | 5 |
| **Ruangjirachuporn/1979/Thailand/Bangkok [147]** | ND | ND | 26 | 30 | C (2-14y) | prosp | ND | Confirmed HI | Nimmannitya | 4 |
| **Salgado/2009/Colombia/Huila [148]** | 2005 | cross | 79 | 19 | C (<13) | prosp | conse | Confirmed ELISA | Yes | 7 |
| **Samsi/1990/Indonesia/Jakarta [149]** | 1987-1988 | cross | 23 | 128 | I,C (0·5-15y) | prosp | conse | Confirmed Virus | Yes | 5 |
| **Sangkawibha/1984/Thailand/Rayong [150]** | 1980 | cross | 22 | 34 | C (1-15y) | prosp | random | confirmed Virus culture, HI | Yes | 8 |
| **Sarasombath/1988/Thailand/Bangkok [151]** | ND | ND | 19 | 18 | I,C (8m-12 yr) | prosp | ND | Confirmed HI | Nimmannitya | 4 |
| **Sarkar/1972/India/Calcutta [152]** | ND | ND | 3 | 5 | C,A (7y-45y) | ND | ND | Confirmed HI, complement fixing (CF) | Shock vs non-shock | 2 |
| **Sathupan/2010/Thailand/Bangkok [153]** | 2003-2005 | ND | 7 | 9 | C (5-15yr) | prosp | ND | Confirmed Serology | Yes | 5 |
| **Scott/1976/Thailand/Bangkok [154]** | 1974 | cross | 48 | 46 | I,C (ND) | prosp | conse | Confirmed Virus culture, HI | Nimmannitya | 7 |
| **Setiawan/1998a/Indonesia/Jakarta [155]** | 1990-1994 | ND | 75 | 73 | I,C (5m-14y) | prosp | ND | Confirmed ELISA, HI, Virus | Yes | 5 |
| **Setiawan/1998b/Indonesia/Jakarta [156]** | ND | ND | 12 | 8 | I,C (5m-14y) | prosp | ND | Confirmed ELISA, HI, Virus | Yes | 5 |
| **Shah/2004/India/Mumbai [157]** | 2003 | cross | 20 | 18 | I,C (4m-12y) | prosp | conse | Confirmed ELISA | Yes | 8 |
| **Shah/2005/India/Mumbai [158]** | 2004 | cross | 14 | 10 | I,C (0-11y) | prosp | conse | Confirmed ELISA | Yes | 7 |
| **Songco/1987/Philippines/Manila [159]** | 1983-1984 | cross | 51 | 84 | I,C (0-18y) | ND | conse | Confirmed HI, Virus | Yes | 6 |
| **Soundravally/2008a/India/Pondicherry [160]** | 2003 | cross | 32 | 75 | A (15-66y) | prosp | conse | Confirmed PCR+ELISA | Yes | 7 |
| **Soundravally/2008b/India/Pondicherry [161]** | 2003 | ND | 21 | 32 | A (26-53y) | prosp | ND | Confirmed ELISA | Yes | 5 |
| **Soundravally/2008c/India/Pondicherry [162]** | 2003 | ND | 32 | 75 | A (15-66y) | prosp | ND | Confirmed PCR+ELISA | Yes | 5 |
| **Srichaikul/1977/Thailand/Bangkok [163]** | ND | ND | 17 | 12 | C (4-13y) | prosp | ND | Confirmed serology | Nimmannitya | 5 |
| **Srichaikul/1989/Thailand/Bangkok [164]** | ND | ND | 18 | 17 | C (5-12y) | prosp | ND | Confirmed Serological | Yes | 4 |
| **Srikiatkhachorn/2007/Thailand/Bangkok [165]** | 2004-2005 | cross | 5 | 16 | C (<15y) | prosp | conse | Confirmed ELISA, PCR | Yes | 8 |
| **Srivastava/1990/India/Delhi [166]** | 1988 | cross | 17 | 7 | C (3-11y) | prosp | conse | Confirmed HI, CF | Yes | 7 |
| **Sumarmo/1986/ Indonesia/Jakarta [167]** | 1975-1983 | cross | 75 | 67 | I,C,A (8m-53y) | prosp | conse | Confirmed Virus culture, HI, ELISA | Nimmannitya | 7 |
| **Suminta/1986/ Indonesia/Yogyakarta [168]** | 1980-1981 | cross | 6 | 19 | C (2-13y) | prosp | conse | ND | Nimmannitya | 5 |
| **Supachokchaiwattana/2007/Thailand/Bangkok [169]** | 2002-2005 | case | 9 | 23 | C (5- 15y) | prosp | ND | Confirmed PCR+ELISA | Yes | 6 |
| **Suvarna/2009/India/Mumbai [170]** | 2006 | cross | 13 | 19 | I,C (1m-18y) | prosp | conse | Confirmed ELISA | Yes | 8 |
| **Suvatte/1973/Thailand/Bangkok [171]** | 1971 | ND | 32 | 117 | C (ND) | prosp | ND | Confirmed serology | Nimmannitya | 4 |
| **Tantracheewathorn/2007/Thailand/Bangkok [172]** | 2003-2005 | case | 55 | 110 | I,C (0·5-14·9 y) | retro | ND | Confirmed ELISA | Yes | 5 |
| **Thakare/1996/India/multisite [173]** | 1990-1995 | ND | 12 | 20 | C,A (6m-60y) | ND | ND | Confirmed HI, ELISA, Virus | Yes | 4 |
| **Thanh/1995/Vietnam/Ho Chi Minh [174]** | 1983 | ND | 1138 | 3200 | C,A (ND) | ND |  | ND | Yes | 2 |
| **Thein/1993/Myanmar/Yangon [175]** | ND | ND | 14 | 19 | C (1-12y) | ND | ND | Confirmed HI | Yes | 3 |
| **Thein/1997/ Myanma/Yangon [176]** | 1984-1988 | cross | 93 | 52 | C (1-10y) | prosp | conse | Confirmed Serology | Yes | 7 |
| **Thomas/2007/India/Punjab [177]** | ND | cross | 4 | 83 | C,A (10-60y) | prosp | conse | Non-confirmed included | Yes | 7 |
| **Thomas/2009/ French-Martinique [178]** | 2007-2008 | cross | 7 | 33 | A (15-88y) | prosp | conse | Confirmed PCR+ELISA | Yes | 7 |
| **Thomas/2010/French-Martinique [179]** | 2005-2008 | cross | 10 | 39 | A (14-91y) | prosp | conse | Confirmed PCR+ELISA | Yes | 7 |
| **Trairatvorakul/2005/Thailand/Bangkok [180]** | 2003-2004 | cross | 37 | 36 | C (ND) | prosp | conse | Confirmed ELISA | Yes | 7 |
| **Trung/2010/Vietnam/HCM [181]** | 2006-2008 | cross | 112^e^ | 532^e^ | A (15-35y) | prosp | conse | Confirmed PCR+ELISA-NS1 | Shock vs non-shock | 8 |
| **Tuchinda/1977/Thailand/Bangkok [182]** | 1971 | ND | 6 | 6 | C (5-11y) | prosp | ND | Confirmed serology | Nimmannitya | 4 |
| **Tupasi/1987/Philippines/Manila [183]** | 1985-1986 | cross | 9 | 63 | I,C,A (8m-53y) | prosp | conse | Confirmed Virus culture, HI, ELISA | Yes | 7 |
| **Uehara/2006/Brazil/ Mato Grosso [184]** | 2002 | cross | 13 | 28 | A (18-88y) | ND | conse | Confirmed ELISA | Yes | 6 |
| **Usawattanakul/1986/Thailand/Bangkok [185]** | 1984 | ND | 42 | 15 | C (3-15y) | prosp | ND | Confirmed HI | Nimmannitya | 5 |
| **Valero/2008/Venezuela/Maracaibo [186]** | ND | ND | 4 | 14 | C,A(6-48y) | prosp | ND | Confirmed ELISA | Yes | 4 |
| **Varavithya/1973/Thailand/Bangkok [187]** | 1970-1971 | cross | 16 | 15 | C (ND) | prosp | conse | Confirmed HI | Nimmannitya | 7 |
| **Vaughn/2000/Thailand/Bangkok&KamphaengPhet[188]** | 1994-1996 | cross | 19 | 67 | C (1-14y) | prosp | conse | Confirmed ELISA, PCR | Yes | 8 |
| **Venzon/1972/Philippines/Manila [189]** | 1966 | cross | 15 | 147 | C,A (0-40y) | ND | conse | Confirmed HI, complement fixed, virus | Nimmannitya | 5 |
| **Wali/1998/India/New Delhi [190]** | 1996 | cross | 8 | 9 | A (14-58y) | prosp | conse | ND | Yes | 7 |
| **Wallace/1980/Malaysia/Kuala Lumpur [191]** | 1973 | ND | 25 | 70 | I,C,A (ND) | retro | ND | Confirmed Virus culture, HI | Nimmannitya | 4 |
| **WHO/1973/Thailand/Bangkok [192]** | 1971 | cross | 36 | 19 | I,C (ND) | prosp | conse | Confirmed Virus culture, HI | Nimmannitya | 7 |
| **Wichmann/2004/Thailand/Chonburi [193]** | 2001 | cross | 40 | 179 | I,C,A (4m-66y) | retro | conse | Confirmed ELISA, HI | Yes | 6 |
| **Widagdo/2008/Indonesia/Jarkarta [194]** | 2005 | cross | 4 | 41 | I,C (75±35m) | prosp | conse | ND | Yes | 7 |
| **Wills/2009/Vietanm/HCM [195]** | 2002-2004 | cross | 33 | 334 | C (2-15) | prosp | conse | Confirmed PCR+ELISA | Shock vs non-shock | 7 |
| **Winter/1968/Thailand/Koh Samui [196]** | 1966 | cross | 14 | 20 | C (1-15y) | prosp | conse | Confirmed HI, complement fixed, virus | Nimmannitya | 7 |
| **Witayathawornwong/2004/Thailand/Petchabun [197]** | 1999-2002 | cross | 362 | 1403 | I,C (2m-15y) | prosp | conse | Non-confirmed and confirmed cases ( ELISA) | Yes | 7 |
| **Wiwanitkit/2004/Thailand/Surin [198]** | 2001 | cross | 4 | 19 | C (9·50 ± 4·12; 8·35 ± 3·60) | prosp | conse | Confirmed Serology | Yes | 7 |

ND: not description; ^a^ Include DF patients; ^b^Limit to liver failure with prothrombin time>2 times; ^c^Limit to intensive patients; ^d^Limit to patient with abdominal pain; ^e^Comparision of shock vs. non-shock; ^f^Limit to thalasemia patient

^g^Cross: cross;Case: case-control; ^h^prosp: prospective; retro: retrospective; ^k^conse: consecutive; IIFA: indirect immunofluorescence assay

^i^WHO 1997 criteria [199]:

DF: Acute fever with two or more of Fever with two or more signs of headache, retro-orbital pain, mayalgia, arthralgia, rash, hemorrhages, and leukopenia accompanied with confirmed dengue virus infection tests.

DHF Grade I: DF signs plus a positive tourniquet test, thrombocytopenia (platelet count< 100,000 cells/mm^3^), and evidence of plasma leakage

DHF Grade II: Above clinical symptoms and laboratory signs plus spontaneous bleeding

DSS Grade III: Above clinical symptoms and laboratory signs plus circulatory failure signs of rapid, weak pulse with low pulsepressure (<20mmHg) or hypotension

DSS Grade IV: Profound shock with undetectable blood pressure and pulse

We defined Grade I and II as DHF, Grade III and IV as DSS.

^i^Nimmannitya criteria [200]:

Grade I: Fever with non specific symptoms; the only hemorrhagic manifestation is a positive tourniquet test

Grade II: Fever and skin hemorrhage or other bleeding such as epistaxis or gumbleeding

Grade III: Circulatory failure signs of rapid, weak pulse with low pulsepressure (<20mmHg) or hypotension

Grade IV: Undetectable blood pressure and pulse

We assigned Grade I and II as DHF, while Grade III and IV as DSS.

**References**

1. Agarwal R, Chaturvedi UC, Misra A, Mukerjee R, Kapoor S, et al. (1998) Production of cytotoxic factor by peripheral blood mononuclear cells (PBMC) in patients with dengue haemorrhagic fever. Clin Exp Immunol 112: 477-481.

2. Agarwal R, Elbishbishi EA, Chaturvedi UC, Nagar R, Mustafa AS (1999) Profile of transforming growth factor-beta 1 in patients with dengue haemorrhagic fever. Int J Exp Pathol 80: 143-149.

3. Aggarwal A, Chandra J, Aneja S, Patwari AK, Dutta AK (1998) An epidemic of dengue hemorrhagic fever and dengue shock syndrome in children in Delhi. Indian Pediatr 35: 727-732.

4. Ahmed FU, Mahmood CB, Sharma JD, Hoque SM, Zaman R, et al. (2001) Dengue and dengue haemorrhagic fever in children during the 2000 outbreak in Chittagong, Bangladesh. Dengue Bulletin 25: 33-39.

5. Arguelles JM, Hernandez M, Mazart I (1987) [Nutritional evaluation of children and adolescents with a diagnosis of dengue]. Bol Oficina Sanit Panam 103: 245-251.

6. Avirutnan P, Punyadee N, Noisakran S, Komoltri C, Thiemmeca S, et al. (2006) Vascular leakage in severe dengue virus infections: a potential role for the nonstructural viral protein NS1 and complement. J Infect Dis 193: 1078-1088.

7. Azad A, Mohammad H, Alam M, Saha A, Ahmed T (2006) Clinical Presentation of Dengue in 150 Admitted Cases in Dhaka Medical College Hospital. Journal of Medicine 7: 3-9.

8. Balmaseda A, Hammond SN, Perez MA, Cuadra R, Solano S, et al. (2005) Short report: assessment of the World Health Organization scheme for classification of dengue severity in Nicaragua. Am J Trop Med Hyg 73: 1059-1062.

9. Basu M, Dasgupta MK, Kundu TK, Sengupta B, De GK, et al. (2007) Profile of pediatric dengue cases from a tertiary care hospital in Kolkata. Indian J Public Health 51: 234-236.

10. Basuki PS (2003) A glance at the von Willebrand factor in dengue virus infection. Southeast Asian J Trop Med Public Health 34: 559-563.

11. Bethell DB, Flobbe K, Cao XT, Day NP, Pham TP, et al. (1998) Pathophysiologic and prognostic role of cytokines in dengue hemorrhagic fever. J Infect Dis 177: 778-782.

12. Bethell DB, Gamble J, Pham PL, Nguyen MD, Tran TH, et al. (2001) Noninvasive measurement of microvascular leakage in patients with dengue hemorrhagic fever. Clin Infect Dis 32: 243-253.

13. Bhattacharjee N, Mukherjee KK, Chakravarti SK, Mukherjee MK, De PN, et al. (1993) Dengue haemorrhagic fever (DHF) outbreak in Calcutta--1990. J Commun Dis 25: 10-14.

14. Bokisch VA, Top FH, Jr., Russell PK, Dixon FJ, Muller-Eberhard HJ (1973) The potential pathogenic role of complement in dengue hemorrhagic shock syndrome. N Engl J Med 289: 996-1000.

15. Boonpucknavig S, Bhamarapravati N, Nimmannitya S, Phalavadhtana A, Siripont J (1976) Immunofluorescent staining of the surfaces of lymphocytes in suspension from patients with dengue hemorrhagic fever. Am J Pathol 85: 37-48.

16. Boonpucknavig S, Bunnag C, Vuttiviroj O, Nimmannitya S (1978) The defined antigen substrate sphere system with direct immunohistoperoxidase for detection of soluble dengue antigen in sera of patients with dengue hemorrhagic fever. Am J Clin Pathol 70: 343-347.

17. Butthep P, Chunhakan S, Tangnararatchakit K, Yoksan S, Pattanapanyasat K, et al. (2006) Elevated soluble thrombomodulin in the febrile stage related to patients at risk for dengue shock syndrome. Pediatr Infect Dis J 25: 894-897.

18. Capeding MR, Paladin FJ (1997) Hospital-based Retrospective Assessment of Dengue Infection among Filipinos. Dengue Bulletin 21.

19. Capeding MR, Paladin FJ, Miranda EG, Navarro XR (1997) Dengue surveillance in Metro Manila. Southeast Asian J Trop Med Public Health 28: 530-534.

20. Chacko B, Subramanian G (2008) Clinical, laboratory and radiological parameters in children with dengue fever and predictive factors for dengue shock syndrome. J Trop Pediatr 54: 137-140.

21. Chairulfatah A, Setiabudi D, Ridad A, Colebunders R (1995) Clinical manifestations of dengue haemorrhagic fever in children in Bandung, Indonesia. Ann Soc Belg Med Trop 75: 291-295.

22. Chairulfatah A, Setiabudi D, Agoes R, van Sprundel M, Colebunders R (2001) Hospital based clinical surveillance for dengue haemorrhagic fever in Bandung, Indonesia 1994-1995. Acta Trop 80: 111-115.

23. Chairulfatah A, Setiabudi D, Agoes R, Colebunders R (2003) Thrombocytopenia and platelet transfusions in dengue haemorrhagic fever and dengue shock syndrome. Dengue Bulletin 27: 138-143.

24. Chaturvedi UC, Agarwal R, Misra A, Mukerjee R, Kapoor S, et al. (1999) Cytotoxic factor in dengue haemorrhagic fever. Med Princ Pract 8: 26-31

25. Chaturvedi UC, Raghupathy R, Pacsa AS, Elbishbishi EA, Agarwal R, et al. (1999) Shift from a Th1-type response to Th2-type in dengue haemorrhagic fever. Current science (Bangalore) 76: 63-69.

26. Chaturvedi UC, Elbishbishi EA, Agarwal R, Mustafa AS (2001) Cytotoxic factor-autoantibodies: possible role in the pathogenesis of dengue haemorrhagic fever. FEMS Immunol Med Microbiol 30: 181-186.

27. Chau TN, Quyen NT, Thuy TT, Tuan NM, Hoang DM, et al. (2008) Dengue in Vietnamese infants--results of infection-enhancement assays correlate with age-related disease epidemiology, and cellular immune responses correlate with disease severity. J Infect Dis 198: 516-524.

28. Chau TN, Anders KL, Lien le B, Hung NT, Hieu LT, et al. (2010) Clinical and virological features of Dengue in Vietnamese infants. PLoS Negl Trop Dis 4: e657.

29. Chaudhary R, Khetan D, Sinha S, Sinha P, Sonker A, et al. (2006) Transfusion support to Dengue patients in a hospital based blood transfusion service in north India. Transfus Apher Sci 35: 239-244.

30. Chhina RS, Goyal O, Chhina DK, Goyal P, Kumar R, et al. (2008) Liver function tests in patients with dengue viral infection. Dengue Bulletins 32: 110-117.

31. Chongsrisawat V, Hutagalung Y, Poovorawan Y (2009) Liver function test results and outcomes in children with acute liver failure due to dengue infection. Southeast Asian J Trop Med Public Health 40: 47-53.

32. Chuansumrit A, Puripokai C, Butthep P, Wongtiraporn W, Sasanakul W, et al. (2010) Laboratory predictors of dengue shock syndrome during the febrile stage. Southeast Asian J Trop Med Public Health 41: 326-332.

33. Chungue E, Poli L, Roche C, Gestas P, Glaziou P, et al. (1994) Correlation between detection of plasminogen cross-reactive antibodies and hemorrhage in dengue virus infection. J Infect Dis 170: 1304-1307.

34. Chunhakan S, Butthep P, Yoksan S, Tangnararatchakit K, Chuansumrit A (2009) Early diagnosis of dengue virus infection by detection of dengue viral antigen in peripheral blood mononuclear cell. Pediatr Infect Dis J 28: 1085-1088.

35. Churdboonchart V, Bhamarapravati N, Futrakul P (1983) Crossed immunoelectrophoresis for the detection of split products of the third complement in dengue hemorrhagic fever. I. Observations in patients' plasma. Am J Trop Med Hyg 32: 569-576.

36. Cohen SN, Halstead SB (1966) Shock associated with dengue infection. I. Clinical and physiologic manifestations of dengue hemorrhagic fever in Thailand, 1964. J Pediatr 68: 448-456.

37. Convers SM, Villar LA, Harker A, Martinez RA, Mendez CX, et al. (2001) Association between clinical gastrointestinal with severe severity in dengue. Infectio 5: 21-30.

38. Corwin AL, Larasati RP, Bangs MJ, Wuryadi S, Arjoso S, et al. (2001) Epidemic dengue transmission in southern Sumatra, Indonesia. Trans R Soc Trop Med Hyg 95: 257-265.

39. Devignot S, Sapet C, Duong V, Bergon A, Rihet P, et al. (2010) Genome-wide expression profiling deciphers host responses altered during dengue shock syndrome and reveals the role of innate immunity in severe dengue. PLoS One 5: e11671.

40. Dewi R, Tumbelaka AR, Sjarif DR (2006) Clinical features of dengue hemorrhagic fever and risk factors of shock event. Paediatrica Indonesiana 46: 144-148.

41. Edelman R, Nimmannitya S, Colman RW, Talamo RC, Top FH, Jr. (1975) Evaluation of the plasma kinin system in dengue hemorrhagic fever. J Lab Clin Med 86: 410-421.

42. Eram S, Setyabudi Y, Sadono TI, Sutrisno DS, Gubler DJ, et al. (1979) Epidemic dengue hemorrhagic fever in rural Indonesia. II. Clinical studies. Am J Trop Med Hyg 28: 711-716.

43. Funahara Y, Sumarmo, Shirahata A, Harun SR, Dharma R, et al. (1985) Suppression of plasma leakage in dengue hemorrhagic fever. ICMR Annals 5 17–37.

44. Glaziou P, Chungue E, Gestas P, Soulignac O, Couter JP, et al. (1992) Dengue fever and dengue shock syndrome in French Polynesia. Southeast Asian J Trop Med Public Health 23: 531-532.

45. Gonzalez D, Castro OE, Kouri G, Perez J, Martinez E, et al. (2005) Classical dengue hemorrhagic fever resulting from two dengue infections spaced 20 years or more apart: Havana, Dengue 3 epidemic, 2001-2002. Int J Infect Dis 9: 280-285.

46. van Gorp EC, Suharti C, Mairuhu AT, Dolmans WM, van Der Ven J, et al. (2002) Changes in the plasma lipid profile as a potential predictor of clinical outcome in dengue hemorrhagic fever. Clin Infect Dis 34: 1150-1153.

47. Gubler DJ, Suharyono W, Lubis I, Eram S, Sulianti Saroso J (1979) Epidemic dengue hemorrhagic fever in rural Indonesia. I. Virological and epidemiological studies. Am J Trop Med Hyg 28: 701-710.

48. Gupta S, Singh SK, Taneja V, Goulatia RK, Bhagat A, et al. (2000) Gall bladder wall edema in serology proven pediatric dengue hemorrhagic fever: a useful diagnostic finding which may help in prognostication. J Trop Pediatr 46: 179-181.

49. Guzman MG, Kouri GP, Bravo J, Soler M, Vazquez S, et al. (1984) Dengue haemorrhagic fever in Cuba. II. Clinical investigations. Trans R Soc Trop Med Hyg 78: 239-241.

50. Ha DQ, Huong VT, Loan HT, Ngu VTT, Dao HTN, et al. (2003) Virological and Serological Surveillance of Dengue Epidemics in 19 Provinces in Southern Viet Nam during 2001. Dengue Bulletin 27: 46-51.

51. Halstead SB, Nimmannitya S, Yamarat C, Russell PK (1967) Hemorrhagic fever in Thailand; recent knowledge regarding etiology. Jpn J Med Sci Biol 20 Suppl: 96-103.

52. Halstead SB, Nimmannitya S, Cohen SN (1970) Observations related to pathogenesis of dengue hemorrhagic fever. IV. Relation of disease severity to antibody response and virus recovered. Yale J Biol Med 42: 311-328.

53. Hanafusa S, Chanyasanha C, Sujirarat D, Khuankhunsathid I, Yaguchi A, et al. (2008) Clinical features and differences between child and adult dengue infections in Rayong Province, southeast Thailand. Southeast Asian J Trop Med Public Health 39: 252-259.

54. Harris E, Videa E, Perez L, Sandoval E, Tellez Y, et al. (2000) Clinical, epidemiologic, and virologic features of dengue in the 1998 epidemic in Nicaragua. Am J Trop Med Hyg 63: 5-11.

55. Hober D, Poli L, Roblin B, Gestas P, Chungue E, et al. (1993) Serum levels of tumor necrosis factor-alpha (TNF-alpha), interleukin-6 (IL-6), and interleukin-1 beta (IL-1 beta) in dengue-infected patients. Am J Trop Med Hyg 48: 324-331.

56. Homchampa P, Sarasombath S, Suvatte V, Vongskul M (1988) Natural killer cells in dengue hemorrhagic fever/dengue shock syndrome. Asian Pac J Allergy Immunol 6: 95-102.

57. Hongsiriwon S (2002) Dengue hemorrhagic fever in infants. Southeast Asian J Trop Med Public Health 33: 49-55.

58. Honsawek S, Kongtawelert P, Pothacharoen P, Khongphatthanayothin A, Chongsrisawat V, et al. (2007) Increased levels of serum hyaluronan in patients with dengue infection. J Infect 54: 225-229.

59. Hoti SL, Soundravally R, Rajendran G, Das LK, Ravi R, et al. (2006) Dengue and dengue haemorrhagic fever outbreak in Pondicherry, South India, during 2003-2004: Emergence of DENV-3. Dengue Bulletin 30: 42-50.

60. Nguyen TH, Lei HY, Nguyen TL, Lin YS, Huang KJ, et al. (2004) Dengue hemorrhagic fever in infants: a study of clinical and cytokine profiles. J Infect Dis 189: 221-232.

61. Nguyen TH, Nguyen TL, Lei HY, Lin YS, Le BL, et al. (2005) Association between sex, nutritional status, severity of dengue hemorrhagic fever, and immune status in infants with dengue hemorrhagic fever. Am J Trop Med Hyg 72: 370-374.

62. Nguyen TH, Nguyen TL, Lei HY, Lin YS, Le BL, et al. (2006) Volume replacement in infants with dengue hemorrhagic fever/dengue shock syndrome. Am J Trop Med Hyg 74: 684-691.

63. Ibrahim F, Abas W, Taib MN, Guan CC, Sulaiman S (2007) A new approach to classify risk in dengue infection using bioelectrical impedance analysis. Dengue Bulletin 31: 58-74.

64. Isarangkura PB, Pongpanich B, Pintadit P, Phanichyakarn P, Valyasevi A (1987) Hemostatic derangement in dengue haemorrhagic fever. Southeast Asian J Trop Med Public Health 18: 331-339.

65. Itha S, Kashyap R, Krishnani N, Saraswat VA, Choudhuri G, et al. (2005) Profile of liver involvement in dengue virus infection. Natl Med J India 18: 127-130.

66. Rivero Jimenez RA, Gomez Arbesu J, Palma Salgado L, Ballester Santovenia JM (1984) [Phagocytic function of polymorphonuclear neutrophil leukocytes in patients with hemorrhagic fever caused by dengue]. Rev Cubana Med Trop 36: 376-384.

67. Juffrie M, van Der Meer GM, Hack CE, Haasnoot K, Sutaryo, et al. (2000) Inflammatory mediators in dengue virus infection in children: interleukin-8 and its relationship to neutrophil degranulation. Infect Immun 68: 702-707.

68. Juffrie M, Meer GM, Hack CE, Haasnoot K, Sutaryo, et al. (2001) Inflammatory mediators in dengue virus infection in children: interleukin-6 and its relation to C-reactive protein and secretory phospholipase A2. Am J Trop Med Hyg 65: 70-75.

69. Juffrie M, Meer GM, Veerman AJP, Thijs LG, Hack CE (2002) Inflammatory mediators in dengue virus infection: Circulating interleukin-12 and interferon-γ. Dengue Bulletin 26: 144-154.

70. Junia J, Garna H, Setiabudi D (2007) Clinical risk factors for dengue shock syndrome in children. Paediatrica Indonesiana 47: 7-11.

71. Kabilan L, Balasubramanian S, Keshava SM, Satyanarayana K (2005) The 2001 dengue epidemic in Chennai. Indian J Pediatr 72: 919-923.

72. Kabra SK, Juneja R, Madhulika, Jain Y, Singhal T, et al. (1998) Myocardial dysfunction in children with dengue haemorrhagic fever. Natl Med J India 11: 59-61.

73. Kabra SK, Jain Y, Pandey RM, Madhulika, Singhal T, et al. (1999) Dengue haemorrhagic fever in children in the 1996 Delhi epidemic. Trans R Soc Trop Med Hyg 93: 294-298.

74. Kalayanarooj S, Nimmannitya S (1989) A study of erythrocyte sedimentation rate in dengue hemorrhagic fever. Southeast Asian J Trop Med Public Health 20: 325-330.

75. Kalayanarooj S, Nimmannitya S, Suntayakorn S, Vaughn DW, Nisalak A, et al. (1999) Can Doctors Make an Accurate Diagnosis of Dengue Infections at an Early Stage? Dengue Bulletin 23: 1-9.

76. Kalayanarooj S, Nimmannitya S (2000) Clinical and Laboratory Presentations of Dengue Patients with Different Serotypes. Dengue Bulletin 24: 53-59.

77. Kalayanarooj SC, V., Nimmannitya S (2002) Dengue Patients at the Children’s Hospital, Bangkok; 1995-1999 Review. Dengue Bulletin 26: 33-43.

78. Kalayanarooj S, Nimmannitya S (2003) Clinical presentations of dengue hemorrhagic fever in infants compared to children. J Med Assoc Thai 86 Suppl 3: S673-680.

79. Kalayanarooj S, Nimmannitya S (2005) Is dengue severity related to nutritional status? Southeast Asian J Trop Med Public Health 36: 378-384.

80. Kalayanarooj S, Gibbons RV, Vaughn D, Green S, Nisalak A, et al. (2007) Blood group AB is associated with increased risk for severe dengue disease in secondary infections. J Infect Dis 195: 1014-1017.

81. Kamath SR, Ranjit S (2006) Clinical features, complications and atypical manifestations of children with severe forms of dengue hemorrhagic fever in South India. Indian J Pediatr 73: 889-895.

82. Kan EF, Rampengan TH (2004) Factors associated with shock in children with dengue hemorrhagic fever. Paediatrica Indonesiana 44: 171-175.

83. Kasim YA, Anky Tri Rini KE, Sumarmo SP (1991) Hyperventilation in children with dengue hemorrhagic fever (DHF). Paediatr Indones 31: 245-252.

84. Chin CK, Kang BH, Liew BK, Cheah PC, Nair R, et al. (1993) Protocol for out-patient management of dengue illness in young adults. J Trop Med Hyg 96: 259-263.

85. Khongphatthanayothin A, Lertsapcharoen P, Supachokchaiwattana P, La-Orkhun V, Khumtonvong A, et al. (2007) Myocardial depression in dengue hemorrhagic fever: prevalence and clinical description. Pediatr Crit Care Med 8: 524-529.

86. Khongphatthanayothin A, Lertsapcharoen P, Supachokchaiwattana P, Satupan P, Thongchaiprasit K, et al. (2005) Hepatosplanchnic circulatory dysfunction in acute hepatic infection: the case of dengue hemorrhagic fever. Shock 24: 407-411.

87. King AD, Nisalak A, Kalayanrooj S, Myint KS, Pattanapanyasat K, et al. (1999) B cells are the principal circulating mononuclear cells infected by dengue virus. Southeast Asian J Trop Med Public Health 30: 718-728.

88. Kittigul L, Pitakarnjanakul P, Sujirarat D, Siripanichgon K (2007) The differences of clinical manifestations and laboratory findings in children and adults with dengue virus infection. J Clin Virol 39: 76-81.

89. Kittigul L, Meethien N, Sujirarat D, Kittigul C, Vasanavat S (1997) Comparison of dengue virus antigens in sera and peripheral blood mononuclear cells from dengue infected patients. Asian Pac J Allergy Immunol 15: 187-191.

90. Kittigul L, Temprom W, Sujirarat D, Kittigul C (2000) Determination of tumor necrosis factor-alpha levels in dengue virus infected patients by sensitive biotin-streptavidin enzyme-linked immunosorbent assay. J Virol Methods 90: 51-57.

91. Kittigul L, Suankeow K, Sujirarat D, Yoksan S (2003) Dengue hemorrhagic fever: knowledge, attitude and practice in Ang Thong Province, Thailand. Southeast Asian J Trop Med Public Health 34: 385-392.

92. Koraka P, Suharti C, Setiati TE, Mairuhu AT, Van Gorp E, et al. (2001) Kinetics of dengue virus-specific serum immunoglobulin classes and subclasses correlate with clinical outcome of infection. J Clin Microbiol 39: 4332-4338.

93. Koraka P, Murgue B, Deparis X, Setiati TE, Suharti C, et al. (2003) Elevated levels of total and dengue virus-specific immunoglobulin E in patients with varying disease severity. J Med Virol 70: 91-98.

94. Koraka P, Murgue B, Deparis X, Van Gorp EC, Setiati TE, et al. (2004) Elevation of soluble VCAM-1 plasma levels in children with acute dengue virus infection of varying severity. J Med Virol 72: 445-450.

95. Koraka P, Lim YP, Shin MD, Setiati TE, Mairuhu AT, et al. (2010) Plasma levels of inter-alpha inhibitor proteins in children with acute Dengue virus infection. PLoS One 5: e9967.

96. Krishnamurti C, Kalayanarooj S, Cutting MA, Peat RA, Rothwell SW, et al. (2001) Mechanisms of hemorrhage in dengue without circulatory collapse. Am J Trop Med Hyg 65: 840-847.

97. de Kruif MD, Setiati TE, Mairuhu AT, Koraka P, Aberson HA, et al. (2008) Differential gene expression changes in children with severe dengue virus infections. PLoS Negl Trop Dis 2: e215.

98. Kurane I, Innis BL, Nimmannitya S, Nisalak A, Meager A, et al. (1991) Activation of T lymphocytes in dengue virus infections. High levels of soluble interleukin 2 receptor, soluble CD4, soluble CD8, interleukin 2, and interferon-gamma in sera of children with dengue. J Clin Invest 88: 1473-1480.

99. Kurane I, Innis BL, Nimmannitya S, Nisalak A, Meager A, et al. (1993) High levels of interferon alpha in the sera of children with dengue virus infection. Am J Trop Med Hyg 48: 222-229.

100. Lan NT, Kikuchi M, Huong VT, Ha do Q, Thuy TT, et al. (2008) Protective and Enhancing HLA Alleles, HLA-DRB1*0901 and HLA-A*24, for Severe Forms of Dengue Virus Infection, Dengue Hemorrhagic Fever and Dengue Shock Syndrome. PLoS Negl Trop Dis 2: e304.

101. Lee IK, Liu JW, Yang KD (2005) Clinical characteristics and risk factors for concurrent bacteremia in adults with dengue hemorrhagic fever. Am J Trop Med Hyg 72: 221-226.

102. Lee YR, Liu MT, Lei HY, Liu CC, Wu JM, et al. (2006) MCP-1, a highly expressed chemokine in dengue haemorrhagic fever/dengue shock syndrome patients, may cause permeability change, possibly through reduced tight junctions of vascular endothelium cells. J Gen Virol 87: 3623-3630.

103. Lee IK, Khor BS, Kee KM, Yang KD, Liu JW (2007) Hyperlipasemia/pancreatitis in adults with dengue hemorrhagic fever. Pancreas 35: 381-382.

104. Lee YR, Hung NT, Liu CC, Huang KJ, Lei HY, et al. (2008) Correlation of IFN-Inducible Protein 10 Levels in Sera with Disease Severity and

Clinical Outcome of the Dengue Patients. American Journal of Infectious Diseases 4: 18-21.

105. Lee IK, Liu JW, Yang KD (2009) Clinical characteristics, risk factors, and outcomes in adults experiencing dengue hemorrhagic fever complicated with acute renal failure. Am J Trop Med Hyg 80: 651-655.

106. Limkittikul K, Yingsakmongkon S, Jittmittraphap A, Chuananon S, Kongphrai Y, et al. (2005) Clinical differences among PCR-proven dengue serotype infections. Southeast Asian J Trop Med Public Health 36: 1432-1438.

107. Lin CF, Lei HY, Liu CC, Liu HS, Yeh TM, et al. (2001) Generation of IgM anti-platelet autoantibody in dengue patients. J Med Virol 63: 143-149.

108. Liu CC, Huang KJ, Lin YS, Yeh TM, Liu HS, et al. (2002) Transient CD4/CD8 ratio inversion and aberrant immune activation during dengue virus infection. J Med Virol 68: 241-252.

109. Loke P, Hammond SN, Leung JM, Kim CC, Batra S, et al. (2010) Gene expression patterns of dengue virus-infected children from nicaragua reveal a distinct signature of increased metabolism. PLoS Negl Trop Dis 4: e710.

110. Long HT, Hibberd ML, Hien TT, Dung NM, Van Ngoc T, et al. (2009) Patterns of gene transcript abundance in the blood of children with severe or uncomplicated dengue highlight differences in disease evolution and host response to dengue virus infection. J Infect Dis 199: 537-546.

111. Lumpaopong A, Kaewplang P, Watanaveeradej V, Thirakhupt P, Chamnanvanakij S, et al. (2010) Electrolyte disturbances and abnormal urine analysis in children with dengue infection. Southeast Asian J Trop Med Public Health 41: 72-76.

112. Mairuhu AT, Peri G, Setiati TE, Hack CE, Koraka P, et al. (2005) Elevated plasma levels of the long pentraxin, pentraxin 3, in severe dengue virus infections. J Med Virol 76: 547-552.

113. Mairuhu AT, Setiati TE, Koraka P, Hack CE, Leyte A, et al. (2005) Increased PAI-1 plasma levels and risk of death from dengue: no association with the 4G/5G promoter polymorphism. Thromb J 3: 17.

114. Malavige GN, Ranatunga PK, Velathanthiri VG, Fernando S, Karunatilaka DH, et al. (2006) Patterns of disease in Sri Lankan dengue patients. Arch Dis Child 91: 396-400.

115. Manaloto CR, Songco RS, Leus CD, Hayes CG (1987) Observations on Hospitalized Dengue Patients in Manila. Phil J Microbiol Infect Dis 16: 37-41.

116. Mekmullica J, Suwanphatra A, Thienpaitoon H, Chansongsakul T, Cherdkiatkul T, et al. (2005) Serum and urine sodium levels in dengue patients. Southeast Asian J Trop Med Public Health 36: 197-199.

117. Mitrakul C (1987) Bleeding problem in dengue haemorrhagic fever: platelets and coagulation changes. Southeast Asian J Trop Med Public Health 18: 407-412.

118. Mohan B, Patwari AK, Anand VK (2000) Hepatic dysfunction in childhood dengue infection. J Trop Pediatr 46: 40-43.

119. Mukerjee R, Chaturvedi UC, Vaughn DW, Kalayauarooj S, Nimmannitya S (1997) Purification and pathogenicity of the cytotoxic factor from the cases of dengue haemorrhagic fever. Curr Sci 72 494–501.

120. Murgue B, Deparis X, Chungue E, Cassar O, Roche C (1999) Dengue: an evaluation of dengue severity in French Polynesia based on an analysis of 403 laboratory-confirmed cases. Trop Med Int Health 4: 765-773.

121. Mustafa AS, Elbishbishi EA, Agarwal R, Chaturvedi UC (2001) Elevated levels of interleukin-13 and IL-18 in patients with dengue hemorrhagic fever. FEMS Immunol Med Microbiol 30: 229-233.

122. Myo K, Soe T, Thein Thein M, Than Nu S, Tin Tin S, et al. (1995) Serum cortisol levels in children with dengue haemorrhagic fever. J Trop Pediatr 41: 295-297.

123. Narayanan M, Aravind MA, Ambikapathy P, Prema R, Jeyapaul MP (2003) Dengue fever – Clinical and laboratory parameters associated with complications. Dengue Bulletin 27: 108-115.

124. Nelson ER, Chulajata R (1965) Danger Signs in Thai Hemorrhagic Fever (Dengue). J Pediatr 67: 463-470.

125. Nguyen TL, Nguyen TH, Tieu NT (1997) The impact of dengue haemorrhagic fever on liver function. Res Virol 148: 273-277.

126. Nimmannitya S (1987) Clinical spectrum and management of dengue haemorrhagic fever. Southeast Asian J Trop Med Public Health 18: 392-397.

127. Nishioka K (1974) Serum complement level in dengue hemorrhagic fever. Allerg Immunol (Leipz) 20-21: 385-392.

128. Ooi ET, Ganesananthan S, Anil R, Kwok FY, Sinniah M (2008) Gastrointestinal manifestations of dengue infection in adults. Med J Malaysia 63: 401-405.

129. Pacsa AS, Agarwal R, Elbishbishi EA, Chaturvedi UC, Nagar R, et al. (2000) Role of interleukin-12 in patients with dengue hemorrhagic fever. FEMS Immunol Med Microbiol 28: 151-155.

130. Pancharoen C, Mekmullica J, Thisyakorn U (2001) Primary dengue infection: what are the clinical distinctions from secondary infection? Southeast Asian J Trop Med Public Health 32: 476-480.

131. Pancharoen C, Rungsarannont A, Thisyakorn U (2002) Hepatic dysfunction in dengue patients with various severity. J Med Assoc Thai 85 Suppl 1: S298-301.

132. Pham TB, Nguyen TH, Vu TQ, Nguyen TL, Malvy D (2007) [Predictive factors of dengue shock syndrome at the children Hospital No. 1, Ho-chi-Minh City, Vietnam]. Bull Soc Pathol Exot 100: 43-47.

133. Pichainarong N, Mongkalangoon N, Kalayanarooj S, Chaveepojnkamjorn W (2006) Relationship between body size and severity of dengue hemorrhagic fever among children aged 0-14 years. Southeast Asian J Trop Med Public Health 37: 283-288.

134. Pongpanich B, Bhanchet P, Phanichyakarn P, Valyasevi A (1973) Studies on dengue hemorrhagic fever. Clinical study: an evaluation of steroids as a treatment. J Med Assoc Thai 56: 6-14.

135. Pongtanakul B, Narkbunnam N, Veerakul G, Sanpakit K, Viprakasit V, et al. (2005) Dengue hemorrhagic fever in patients with thalassemia. J Med Assoc Thai 88 Suppl 8: S80-85.

136. Potts JA, Gibbons RV, Rothman AL, Srikiatkhachorn A, Thomas SJ, et al. (2010) Prediction of dengue disease severity among pediatric Thai patients using early clinical laboratory indicators. PLoS Negl Trop Dis 4: e769.

137. Preeyasombat C, Bunnag P, Sirinavin S, Mahachoklertwattana P, Sriphrapradang A (1990) Plasma prostacyclin (PGI2) in dengue hemorrhagic fever. Southeast Asian J Trop Med Public Health 21: 383-387.

138. Preeyasombat C, Treepongkaruna S, Sriphrapradang A, Choubtum L (1999) The role of prostacyclin (PGI2) and thromboxane A2 (TXA2) in pathogenesis of dengue hemorrhagic fever (DHF). J Med Assoc Thai 82 Suppl 1: S16-21.

139. Pushpa V, Venkatadesikalu M, Mohan S, Cherian T, John TJ, et al. (1998) An epidemic of dengue haemorrhagic fever/dengue shock syndrome in tropical India. Ann Trop Paediatr 18: 289-293.

140. Puspanjono MT, Latief A, Tumbelaka AR, Sastroasmoro S, Gunardi H (2007) Comparison of serial blood lactate level between dengue shock syndrome and dengue hemorrhagic fever (evaluation of prognostic value). Paediatrica Indonesiana 47: 150-155.

141. Raghupathy R, Chaturvedi UC, Al-Sayer H, Elbishbishi EA, Agarwal R, et al. (1998) Elevated levels of IL-8 in dengue hemorrhagic fever. J Med Virol 56: 280-285.

142. Rajendiran S, Lakshamanappa HS, Zachariah B, Nambiar S (2008) Desialylation of plasma proteins in severe dengue infection: possible role of oxidative stress. Am J Trop Med Hyg 79: 372-377.

143. Ratageri VH, Shepur TA, Wari PK, Chavan SC, Mujahid IB, et al. (2005) Clinical profile and outcome of Dengue fever cases. Indian J Pediatr 72: 705-706.

144. Ray G, Kumar V, Kapoor AK, Dutta AK, Batra S (1999) Status of antioxidants and other biochemical abnormalities in children with dengue fever. J Trop Pediatr 45: 4-7.

145. De Rivera IL, Parham L, Murillo W, Moncada W, Vazquez S (2008) Humoral immune response of dengue hemorrhagic fever cases in children from Tegucigalpa, Honduras. Am J Trop Med Hyg 79: 262-266.

146. Rongrungruang Y, Leelarasamee A (2001) Characteristics and outcomes of adult patients with symptomatic dengue virus infections. J Infect Dis Antimicrol Agents 18: 19-23.

147. Ruangjirachuporn W, Boonpucknavig S, Nimmanitya S (1979) Circulating immune complexes in serum from patients with dengue haemorrhagic fever. Clin Exp Immunol 36: 46-53.

148. Salgado DM, Panqueba CA, Castro D, M RV, Rodriguez JA (2009) [Myocarditis in children affected by dengue hemorrhagic fever in a teaching hospital in Colombia]. Rev Salud Publica (Bogota) 11: 591-600.

149. Samsi TK, Wulur H, Sugianto D, Bartz CR, Tan R, et al. (1990) Some clinical and epidemiological observations on virologically confirmed dengue hemorrhagic fever. Paediatr Indones 30: 293-303.

150. Sangkawibha N, Rojanasuphot S, Ahandrik S, Viriyapongse S, Jatanasen S, et al. (1984) Risk factors in dengue shock syndrome: a prospective epidemiologic study in Rayong, Thailand. I. The 1980 outbreak. Am J Epidemiol 120: 653-669.

151. Sarasombath S, Suvatte V, Homchampa P (1988) Kinetics of lymphocyte subpopulations in dengue hemorrhagic fever/dengue shock syndrome. Southeast Asian J Trop Med Public Health 19: 649-656.

152. Sarkas JK, Chakravarty SK, Sarkar RK (1972) Sporadic cases of haemorrhage and-or shock during dengue epidemics. Trans R Soc Trop Med Hyg 66: 875-877.

153. Sathupan P, Khongphattanayothin A, Srisai J, Srikaew K, Poovorawan Y (2007) The role of vascular endothelial growth factor leading to vascular leakage in children with dengue virus infection. Ann Trop Paediatr 27: 179-184.

154. Scott RM, Nimmannitya S, Bancroft WH, Mansuwan P (1976) Shock syndrome in primary dengue infections. Am J Trop Med Hyg 25: 866-874.

155. Setiawan MW, Samsi TK, Wulur H, Sugianto D, Pool TN (1998) Dengue haemorrhagic fever: ultrasound as an aid to predict the severity of the disease. Pediatr Radiol 28: 1-4.

156. Setiawan MW, Samsi TK, Wulur H, Sugianto D, Pool TN (1998) Epigastric pain and sonographic assessment of the pancreas in dengue hemorrhagic fever. J Clin Ultrasound 26: 257-259.

157. Shah I, Deshpande GC, Tardeja PN (2004) Outbreak of dengue in Mumbai and predictive markers for dengue shock syndrome. J Trop Pediatr 50: 301-305.

158. Shah I, Katira B (2005) Clinical and Laboratory Abnormalities due to Dengue in Hospitalized Children in Mumbai in 2004. Dengue Bulletin 29: 90–96.

159. Songco RS, Hayes CG, Leus CD, Manaloto CO (1987) Dengue fever/dengue haemorrhagic fever in Filipino children: clinical experience during the 1983-1984 epidemic. Southeast Asian J Trop Med Public Health 18: 284-290.

160. Soundravally R, Hoti SL (2008) Polymorphisms of the TAP 1 and 2 gene may influence clinical outcome of primary dengue viral infection. Scand J Immunol 67: 618-625.

161. Soundravally R, Sankar P, Bobby Z, Hoti SL (2008) Oxidative stress in severe dengue viral infection: association of thrombocytopenia with lipid peroxidation. Platelets 19: 447-454.

162. Soundravally R, Hoti SL (2008) Significance of transporter associated with antigen processing 2 (TAP2) gene polymorphisms in susceptibility to dengue viral infection. J Clin Immunol 28: 256-262.

163. Srichaikul T, Nimmanitaya S, Artchararit N, Siriasawakul T, Sungpeuk P (1977) Fibrinogen metabolism and disseminated intravascular coagulation in dengue hemorrhagic fever. Am J Trop Med Hyg 26: 525-532.

164. Srichaikul T, Nimmannitya S, Sripaisarn T, Kamolsilpa M, Pulgate C (1989) Platelet function during the acute phase of dengue hemorrhagic fever. Southeast Asian J Trop Med Public Health 20: 19-25.

165. Srikiatkhachorn A, Krautrachue A, Ratanaprakarn W, Wongtapradit L, Nithipanya N, et al. (2007) Natural history of plasma leakage in dengue hemorrhagic fever: a serial ultrasonographic study. Pediatr Infect Dis J 26: 283-290; discussion 291-282.

166. Srivastava VK, Suri S, Bhasin A, Srivastava L, Bharadwaj M (1990) An epidemic of dengue haemorrhagic fever and dengue shock syndrome in Delhi: a clinical study. Ann Trop Paediatr 10: 329-334.

167. Sumarmo, Wuryadi S, Gubler DJ (1986) Clinical observations on hospitalized patients with virologically confirmed dengue hemorrhagic fever in Jakarta, Indonesia 1975-1983. Paediatr Indones 26: 137-151.

168. Suminta, Soenarto Y, Widiarto, Sutaryo, Ismangoen (1986) Plasma protein in dengue hemorrhagic fever/dengue shock syndrome. Paediatr Indones 26: 56-61.

169. Supachokchaiwattana P, La-Orkhun V, Arj-ong S, Sirichonkolthong B, Lertsapcharoen P, et al. (2007) Reversible impairment of global cardiac function during toxic stage of dengue hemorrhagic fever and dengue shock syndrome. Thai heart J 20: 180-187.

170. Suvarna JC, Rane PP (2009) Serum lipid profile: a predictor of clinical outcome in dengue infection. Trop Med Int Health 14: 576-585.

171. Suvatte V, Pongpipat D, Tuchinda S, Ratanawongs A, Tuchinda P, et al. (1973) Studies on serum complement C3 and fibrin degradation products in Thai hemorrhagic fever. J Med Assoc Thai 56: 24-32.

172. Tantracheewathorn T, Tantracheewathorn S (2007) Risk factors of dengue shock syndrome in children. J Med Assoc Thai 90: 272-277.

173. Thakare J, Walhekar B, Banerjee K (1996) Hemorrhagic manifestations and encephalopathy in cases of dengue in India. Southeast Asian J Trop Med Public Health 27: 471-475.

174. Nguyen DT, Pham NG (1994) Epidemiology and clinical features of dengue haemorrhagic fever in Ho Chi Minh City and the Center for Tropical Diseases; Viet Nam. Tropical Medicine 36: 177-186.

175. Thein S, Aaskov J, Myint TT, Shwe TN, Saw TT, et al. (1993) Changes in levels of anti-dengue virus IgG subclasses in patients with disease of varying severity. J Med Virol 40: 102-106.

176. Thein S, Aung MM, Shwe TN, Aye M, Zaw A, et al. (1997) Risk factors in dengue shock syndrome. Am J Trop Med Hyg 56: 566-572.

177. Thomas EA, John M, Bhatia A (2007) Cutaneous manifestations of dengue viral infection in Punjab (north India). Int J Dermatol 46: 715-719.

178. Thomas L, Kaidomar S, Kerob-Bauchet B, Moravie V, Brouste Y, et al. (2009) Prospective observational study of low thresholds for platelet transfusion in adult dengue patients. Transfusion 49: 1400-1411.

179. Thomas L, Brouste Y, Najioullah F, Hochedez P, Hatchuel Y, et al. Prospective and descriptive study of adult dengue cases in an emergency department, in Martinique. Med Mal Infect 40: 480-489.

180. Trairatvorakul P, Chongsrisawat V, Ngamvasinont D, Asawarachun D, Nantasook J, et al. (2005) Serum nitric oxide in children with dengue infection. Asian Pac J Allergy Immunol 23: 115-119.

181. Trung DT, Thao le TT, Hien TT, Hung NT, Vinh NN, et al. (2010) Liver involvement associated with dengue infection in adults in Vietnam. Am J Trop Med Hyg 83: 774-780.

182. Tuchinda M, Dhorranintra B, Tuchinda P (1977) Histamine content in 24-hour urine in patients with dengue haemorrhagic fever. Southeast Asian J Trop Med Public Health 8: 80-83.

183. Tupasi T, Montalban C, Zeta R, Moriles R, Tortes C, et al. (1987) Virological and clinical studies in dengue infection. Phil J Microbiol Infect Dis 16: 1 -4.

184. Uehara PM, da Cunha RV, Pereira GR, de Oliveira PA (2006) [Liver involvement in patients with dengue hemorrhagic fever: a rare phenomenon?]. Rev Soc Bras Med Trop 39: 544-547.

185. Usawattanakul W, Nimmannitya S, Sarabenjawong K, Tharavanij S (1986) Endotoxin and dengue haemorrhagic fever. Southeast Asian J Trop Med Public Health 17: 8-12.

186. Valero N, Larreal Y, Espina LM, Reyes I, Maldonado M, et al. (2008) Elevated levels of interleukin-2 receptor and intercellular adhesion molecule 1 in sera from a venezuelan cohort of patients with dengue. Arch Virol 153: 199-203.

187. Varavithya W, Manu P, Kittikool J, Phongbetchara P, Kashemsant C (1973) Studies on dengue hemorrhagic fever. II. Electrolyte study. J Med Assoc Thai 56: 15-23.

188. Vaughn DW, Green S, Kalayanarooj S, Innis BL, Nimmannitya S, et al. (2000) Dengue viremia titer, antibody response pattern, and virus serotype correlate with disease severity. J Infect Dis 181: 2-9.

189. Venzon EL, Rudnick A, Marchette NJ, Fable AE, Dukellis E (1966) The greater Manila dengue hemorrhagic fever epidemic of 1966. J Phil Med Assoc 48: 297-313.

190. Wali JP, Biswas A, Chandra S, Malhotra A, Aggarwal P, et al. (1998) Cardiac involvement in Dengue Haemorrhagic Fever. Int J Cardiol 64: 31-36.

191. Wallace HG, Lim TW, Rudnick A, Knudsen AB, Cheong WH, et al. (1980) Dengue hemorrhagic fever in Malaysia: the 1973 epidemic. Southeast Asian J Trop Med Public Health 11: 1-13.

192. (1973) Pathogenetic mechanisms in dengue haemorrhagic fever: report of an international collaborative study. Bull World Health Organ 48: 117-133.

193. Wichmann O, Hongsiriwon S, Bowonwatanuwong C, Chotivanich K, Sukthana Y, et al. (2004) Risk factors and clinical features associated with severe dengue infection in adults and children during the 2001 epidemic in Chonburi, Thailand. Trop Med Int Health 9: 1022-1029.

194. Widagdo (2008) Blood zinc levels and clinical severity of dengue hemorrhagic fever in children. Southeast Asian J Trop Med Public Health 39: 610-616.

195. Wills B, Tran VN, Nguyen TH, Truong TT, Tran TN, et al. (2009) Hemostatic changes in Vietnamese children with mild dengue correlate with the severity of vascular leakage rather than bleeding. Am J Trop Med Hyg 81: 638-644.

196. Winter PE, Yuill TM, Udomsakdi S, Gould D, Nantapanich S, et al. (1968) An insular outbreak of dengue hemorrhagic fever. I. Epidemiologic observations. Am J Trop Med Hyg 17: 590-599.

197. Witayathawornwong P (2004) Dengue Haemorrhagic Fever with Encephalopathy/Fatality at Petchabun Hospital A three-year Prospective Study (1999-2002). Dengue Bulletin 28: 77-86.

198. Wiwanitkit V, Manusvanich P (2004) Can hematocrit and platelet determination on admission predict shock in hospitalized children with dengue hemorrhagic fever? A clinical observation from a small outbreak. Clin Appl Thromb Hemost 10: 65-67.

199. (1997) WHO. Dengue haemorrhagic fever: Diagnosis, treatment, prevention and control.; Organization WH, editor. Geneva: .

200. Nimmannitya S, Halstead SB, Cohen SN, Margiotta MR (1969) Dengue and chikungunya virus infection in man in Thailand, 1962-1964. I. Observations on hospitalized patients with hemorrhagic fever. Am J Trop Med Hyg 18: 954-971.
