# Supplementary material for: Factors Associated with Dengue Shock Syndrome: A Systematic Review and Meta-Analysis
Source: PLoS Negl Trop Dis. 2013 Sep 26;7(9):e2412. doi: 10.1371/journal.pntd.0002412 (PMC3784477; doi:10.1371/journal.pntd.0002412)
Supplement: Table S3 — Factors that was only investigated in one study or several studies that cannot be extracted. The association of the factor with DSS was derived from the original study. (DOC) [file pntd.0002412.s005.doc]

**Table S3. Factors that was only investigated in one study or several studies that cannot be extracted.**

| **Factors** | **Association with DSS** |
| --- | --- |
| History of Dengue [1] | NS |
| History of hypertension [2] | NS |
| History of diabetes [2] | NS |
| History of sickle cell anemia [2] | NS |
| History of asthma [2] | NS |
| History of peptic ulcer [2] | NS |
| Day of defervescence [3] | NS |
| Oral candidiasis [4] | NS |
| Vaccination against Japanese encephalitis B [5] | NS |
| Body mass index (BMI) [6] | NS |
| Cyanosis [7] | Positive association |
| Restless | Positive association |
| Retro - orbital pain [8] | NS |
| Anorexia [8] | NS |
| Abdominal distention [9] | NS |
| Constipation [5] | NS |
| Lymph nodes [10] | NS |
| ST elevation in ECG [11] | Positive association |
| T inversion in ECG [11] | Positive association |
| Global hypokinesia [11] | NS |
| Retrovesicular fluid [12] | Positive association |
| Pericardial effusion [13] | NS |
| Dilated portal vein [14] | Positive association |
| Pancreatic enlargement [13] | Positive association |
| Hypoxemia [15] | NS |
| Blood pH | NS |
| Blood chloride [16] | NS |
| Blood calcium [16] | NS |
| Blood magnesium [16] | NS |
| Blood zinc [6] | NS |
| Isovolumetric venous pressure [17] | NS |
| Coefficient of microvascular permeability [17] | NS |
| Osmolarity [16] | NS |
| Colloid osmotic pressure (Oncotic pressure) [17] | Negative association |
| Bioelectrical impedance analysis (reactance value) [18] | Negative association |
| Oliguria [19] | NS |
| Urine gravity [20] | NS |
| Urine sodium [21] | NS |
| Ketonuria [20] | NS |
| Urine cast or hyalic [7] | NS |
| Urine histamine [22] | Positive association |
| Bleeding time [23] | NS |
| Anti-thrombin III [24] | Negative association |
| Von Willebrand factor [25] | Positive association |
| Ristocetin cofactor activity [25] | NS |
| Plasminogen [26] | NS |
| Plasma plasminogen activator inhibitor type I [27] | NS |
| alpha 2-antiplasmin [26] | NS |
| Prothrombin fragment F1.2 [26] | Negative association |
| Protein C [28] | NS |
| Protein S [28] | NS |
| Inter - α Inhibitor Protein [24] | Negative association |
| Gamma-glutamyltransferase [29] | NS |
| Blood transferrin [30] | NS |
| Blood cortisol [31] | NS |
| Platelet aggregation [32] | NS |
| Lymphocyte count [33,34] | NS |
| Lymphocyte% [6] | NS |
| Neutrophil count [33] | Negative association |
| Neutrophils (%) [6] | NS |
| Phagocytic function of peripheral neutrophil [35] | NS |
| Circulating endothelial cells [36] | Positive association |
| CD4/CD8 ratio [37] | NS |
| CD4+CD69+ T cells [38] | Positive association |
| CD8+CD69+ T cells [38] | Positive association |
| HNK-1 + (NK, natural killer cells) [39] | Negative association |
| CD16+CD56+CD69+NK cells [38] | NS |
| CD16+CD56+ NK cells [40] | NS |
| Blood immune complex level [41] | NS |
| Complement C1 [42] | NS |
| Complement C1s [30] | NS |
| Complement C1q [30] | Negative association |
| Complement C2 [42] | NS |
| Complement C2 [42] | NS |
| Complement C3IA [42] | NS |
| Catabolic rate of C3 [42] | Positive association |
| Complement C3 proactivator [30] | NS |
| Complement split C3 product [43] | Positive association |
| Total complement activity (CH50) [42] | NS |
| Bradykinin [44] | NS |
| Kallikrein inhibitors [44] | NS |
| Prekallikrein [44] | Negative association |
| Anti-plasminogen [45] | NS |
| Endotoxin [46] | NS |
| Decreased factor XII [44] | NS |
| Globulin [47] | Negative association |
| Total IgE [48] | Positive association |
| Dengue specific IgE [48] | NS |
| Transforming growth factors - beta1 (TGF- beta1) [49] | NS |
| IFN-α [50] | NS |
| IL-1β [38] | NS |
| IL-12 (total) [49] | Negative association |
| IL-12p40 [51] | Negative association |
| IL-12p70 [38] | Positive association |
| Interleukin-13 [52] | Positive association |
| Interleukin-18 [52] | Positive association |
| Blood soluble TNF receptor p55 [53] | Positive association |
| Blood soluble TNF receptor p75 [53] | Positive association |
| Blood soluble ICAM-1 [53,54] | NS |
| Blood soluble VCAM-1 [55] | Positive association |
| Vascular endothelial growth factors (VEGF) [56] | NS |
| Soluble thrombomodulin [36] | Positive association |
| Monokine induced by interferon gama [38] | NS |
| RANTES [38] | NS |
| Transforming growth factor-beta 1 (TGF- beta1) [57] | Positive association |
| Pentraxin 3 [58] | Positive association |
| Soluble CD4 [59] | NS |
| Soluble CD8 [59] | NS |
| Shift from Th2 to Th1 in immune response [60] | NS |
| Thromboxane B2 [61] | Negative association |
| Blood group O [62] | NS |
| A [62] | NS |
| B [62] | NS |
| AB [62] | Positive association |
| Very-low-density lipoprotein (VLDL) [63] | NS |
| Autoantibody against human cytotoxic factor [64] | Negative association |
| Production of human cytotoxic factor by PBMC [64] | NS |
| Plasma secretory phospholipase A2 [65] | Positive association |
| Blood elastase [66] | Positive association |
| Blood lactoferrin [66] | Positive association |
| Blood CRP [53,65] | NS |
| Creatine phosphokinase [67] | Positive association |
| Nitric oxide [68] | Positive association |
| Superoxide dismutase [67] | NS |
| Glutathione peroxidase [67] | NS |
| Total plasma antioxidant [69] | NS |
| Malondialdehyde [69] | Positive association (only at day 7) |
| Sialic acid content of serum protein [70] | Negative association |
| Protein carbonyls [70] | Positive association |
| Protein-bound sulphydryl groups [70] | Negative association |
| Blood soluble P-selectin [26] | NS |
| Blood hyaluronan [71] | NS |
| Dengue nonstructural protein NS1 [72] | NS |
| Positive dengue antigen in acute sera [73] | NS |

The association of the factor with DSS was derived from the original study.

NS: non-significant association

**References**

1. Pichainarong N, Mongkalangoon N, Kalayanarooj S, Chaveepojnkamjorn W (2006) Relationship between body size and severity of dengue hemorrhagic fever among children aged 0-14 years. Southeast Asian J Trop Med Public Health 37: 283-288.

2. Gonzalez D, Castro OE, Kouri G, Perez J, Martinez E, et al. (2005) Classical dengue hemorrhagic fever resulting from two dengue infections spaced 20 years or more apart: Havana, Dengue 3 epidemic, 2001-2002. Int J Infect Dis 9: 280-285.

3. Wills B, Tran VN, Nguyen TH, Truong TT, Tran TN, et al. (2009) Hemostatic changes in Vietnamese children with mild dengue correlate with the severity of vascular leakage rather than bleeding. Am J Trop Med Hyg 81: 638-644.

4. Malavige GN, Ranatunga PK, Velathanthiri VG, Fernando S, Karunatilaka DH, et al. (2006) Patterns of disease in Sri Lankan dengue patients. Arch Dis Child 91: 396-400.

5. Pham TB, Nguyen TH, Vu TQ, Nguyen TL, Malvy D (2007) [Predictive factors of dengue shock syndrome at the children Hospital No. 1, Ho-chi-Minh City, Vietnam]. Bull Soc Pathol Exot 100: 43-47.

6. Widagdo (2008) Blood zinc levels and clinical severity of dengue hemorrhagic fever in children. Southeast Asian J Trop Med Public Health 39: 610-616.

7. Nelson ER, Chulajata R (1965) Danger Signs in Thai Hemorrhagic Fever (Dengue). J Pediatr 67: 463-470.

8. Azad A, Mohammad H, Alam M, Saha A, Ahmed T (2006) Clinical Presentation of Dengue in 150 Admitted Cases in Dhaka Medical College Hospital. Journal of Medicine 7: 3-9.

9. Ahmed FU, Mahmood CB, Sharma JD, Hoque SM, Zaman R, et al. (2001) Dengue and dengue haemorrhagic fever in children during the 2000 outbreak in Chittagong, Bangladesh. Dengue Bulletin 25: 33-39.

10. Narayanan M, Aravind MA, Ambikapathy P, Prema R, Jeyapaul MP (2003) Dengue fever – Clinical and laboratory parameters associated with complications. Dengue Bulletin 27: 108-115.

11. Wali JP, Biswas A, Chandra S, Malhotra A, Aggarwal P, et al. (1998) Cardiac involvement in Dengue Haemorrhagic Fever. Int J Cardiol 64: 31-36.

12. Srikiatkhachorn A, Krautrachue A, Ratanaprakarn W, Wongtapradit L, Nithipanya N, et al. (2007) Natural history of plasma leakage in dengue hemorrhagic fever: a serial ultrasonographic study. Pediatr Infect Dis J 26: 283-290; discussion 291-282.

13. Setiawan MW, Samsi TK, Wulur H, Sugianto D, Pool TN (1998) Dengue haemorrhagic fever: ultrasound as an aid to predict the severity of the disease. Pediatr Radiol 28: 1-4.

14. Khongphatthanayothin A, Lertsapcharoen P, Supachokchaiwattana P, Satupan P, Thongchaiprasit K, et al. (2005) Hepatosplanchnic circulatory dysfunction in acute hepatic infection: the case of dengue hemorrhagic fever. Shock 24: 407-411.

15. Kasim YA, Anky Tri Rini KE, Sumarmo SP (1991) Hyperventilation in children with dengue hemorrhagic fever (DHF). Paediatr Indones 31: 245-252.

16. Varavithya W, Manu P, Kittikool J, Phongbetchara P, Kashemsant C (1973) Studies on dengue hemorrhagic fever. II. Electrolyte study. J Med Assoc Thai 56: 15-23.

17. Bethell DB, Gamble J, Pham PL, Nguyen MD, Tran TH, et al. (2001) Noninvasive measurement of microvascular leakage in patients with dengue hemorrhagic fever. Clin Infect Dis 32: 243-253.

18. Ibrahim F, Abas W, Taib MN, Guan CC, Sulaiman S (2007) A new approach to classify risk in dengue infection using bioelectrical impedance analysis. Dengue Bulletin 31: 58-74.

19. Chacko B, Subramanian G (2008) Clinical, laboratory and radiological parameters in children with dengue fever and predictive factors for dengue shock syndrome. J Trop Pediatr 54: 137-140.

20. Lumpaopong A, Kaewplang P, Watanaveeradej V, Thirakhupt P, Chamnanvanakij S, et al. (2010) Electrolyte disturbances and abnormal urine analysis in children with dengue infection. Southeast Asian J Trop Med Public Health 41: 72-76.

21. Mekmullica J, Suwanphatra A, Thienpaitoon H, Chansongsakul T, Cherdkiatkul T, et al. (2005) Serum and urine sodium levels in dengue patients. Southeast Asian J Trop Med Public Health 36: 197-199.

22. Tuchinda M, Dhorranintra B, Tuchinda P (1977) Histamine content in 24-hour urine in patients with dengue haemorrhagic fever. Southeast Asian J Trop Med Public Health 8: 80-83.

23. Halstead SB, Nimmannitya S, Cohen SN (1970) Observations related to pathogenesis of dengue hemorrhagic fever. IV. Relation of disease severity to antibody response and virus recovered. Yale J Biol Med 42: 311-328.

24. Koraka P, Lim YP, Shin MD, Setiati TE, Mairuhu AT, et al. (2010) Plasma levels of inter-alpha inhibitor proteins in children with acute Dengue virus infection. PLoS One 5: e9967.

25. Chuansumrit A, Puripokai C, Butthep P, Wongtiraporn W, Sasanakul W, et al. (2010) Laboratory predictors of dengue shock syndrome during the febrile stage. Southeast Asian J Trop Med Public Health 41: 326-332.

26. Krishnamurti C, Kalayanarooj S, Cutting MA, Peat RA, Rothwell SW, et al. (2001) Mechanisms of hemorrhage in dengue without circulatory collapse. Am J Trop Med Hyg 65: 840-847.

27. Mairuhu AT, Setiati TE, Koraka P, Hack CE, Leyte A, et al. (2005) Increased PAI-1 plasma levels and risk of death from dengue: no association with the 4G/5G promoter polymorphism. Thromb J 3: 17.

28. Nguyen TH, Lei HY, Nguyen TL, Lin YS, Huang KJ, et al. (2004) Dengue hemorrhagic fever in infants: a study of clinical and cytokine profiles. J Infect Dis 189: 221-232.

29. Uehara PM, da Cunha RV, Pereira GR, de Oliveira PA (2006) [Liver involvement in patients with dengue hemorrhagic fever: a rare phenomenon?]. Rev Soc Bras Med Trop 39: 544-547.

30. (1973) Pathogenetic mechanisms in dengue haemorrhagic fever: report of an international collaborative study. Bull World Health Organ 48: 117-133.

31. Myo K, Soe T, Thein Thein M, Than Nu S, Tin Tin S, et al. (1995) Serum cortisol levels in children with dengue haemorrhagic fever. J Trop Pediatr 41: 295-297.

32. Srichaikul T, Nimmannitya S, Sripaisarn T, Kamolsilpa M, Pulgate C (1989) Platelet function during the acute phase of dengue hemorrhagic fever. Southeast Asian J Trop Med Public Health 20: 19-25.

33. Devignot S, Sapet C, Duong V, Bergon A, Rihet P, et al. (2010) Genome-wide expression profiling deciphers host responses altered during dengue shock syndrome and reveals the role of innate immunity in severe dengue. PLoS One 5: e11671.

34. Sarasombath S, Suvatte V, Homchampa P (1988) Kinetics of lymphocyte subpopulations in dengue hemorrhagic fever/dengue shock syndrome. Southeast Asian J Trop Med Public Health 19: 649-656.

35. Rivero Jimenez RA, Gomez Arbesu J, Palma Salgado L, Ballester Santovenia JM (1984) [Phagocytic function of polymorphonuclear neutrophil leukocytes in patients with hemorrhagic fever caused by dengue]. Rev Cubana Med Trop 36: 376-384.

36. Butthep P, Chunhakan S, Tangnararatchakit K, Yoksan S, Pattanapanyasat K, et al. (2006) Elevated soluble thrombomodulin in the febrile stage related to patients at risk for dengue shock syndrome. Pediatr Infect Dis J 25: 894-897.

37. Liu CC, Huang KJ, Lin YS, Yeh TM, Liu HS, et al. (2002) Transient CD4/CD8 ratio inversion and aberrant immune activation during dengue virus infection. J Med Virol 68: 241-252.

38. Chau TN, Quyen NT, Thuy TT, Tuan NM, Hoang DM, et al. (2008) Dengue in Vietnamese infants--results of infection-enhancement assays correlate with age-related disease epidemiology, and cellular immune responses correlate with disease severity. J Infect Dis 198: 516-524.

39. Homchampa P, Sarasombath S, Suvatte V, Vongskul M (1988) Natural killer cells in dengue hemorrhagic fever/dengue shock syndrome. Asian Pac J Allergy Immunol 6: 95-102.

40. Long HT, Hibberd ML, Hien TT, Dung NM, Van Ngoc T, et al. (2009) Patterns of gene transcript abundance in the blood of children with severe or uncomplicated dengue highlight differences in disease evolution and host response to dengue virus infection. J Infect Dis 199: 537-546.

41. Ruangjirachuporn W, Boonpucknavig S, Nimmanitya S (1979) Circulating immune complexes in serum from patients with dengue haemorrhagic fever. Clin Exp Immunol 36: 46-53.

42. Nishioka K (1974) Serum complement level in dengue hemorrhagic fever. Allerg Immunol (Leipz) 20-21: 385-392.

43. Churdboonchart V, Bhamarapravati N, Futrakul P (1983) Crossed immunoelectrophoresis for the detection of split products of the third complement in dengue hemorrhagic fever. I. Observations in patients' plasma. Am J Trop Med Hyg 32: 569-576.

44. Edelman R, Nimmannitya S, Colman RW, Talamo RC, Top FH, Jr. (1975) Evaluation of the plasma kinin system in dengue hemorrhagic fever. J Lab Clin Med 86: 410-421.

45. Chungue E, Poli L, Roche C, Gestas P, Glaziou P, et al. (1994) Correlation between detection of plasminogen cross-reactive antibodies and hemorrhage in dengue virus infection. J Infect Dis 170: 1304-1307.

46. Usawattanakul W, Nimmannitya S, Sarabenjawong K, Tharavanij S (1986) Endotoxin and dengue haemorrhagic fever. Southeast Asian J Trop Med Public Health 17: 8-12.

47. Pancharoen C, Rungsarannont A, Thisyakorn U (2002) Hepatic dysfunction in dengue patients with various severity. J Med Assoc Thai 85 Suppl 1: S298-301.

48. Koraka P, Murgue B, Deparis X, Setiati TE, Suharti C, et al. (2003) Elevated levels of total and dengue virus-specific immunoglobulin E in patients with varying disease severity. J Med Virol 70: 91-98.

49. Pacsa AS, Agarwal R, Elbishbishi EA, Chaturvedi UC, Nagar R, et al. (2000) Role of interleukin-12 in patients with dengue hemorrhagic fever. FEMS Immunol Med Microbiol 28: 151-155.

50. Kurane I, Innis BL, Nimmannitya S, Nisalak A, Meager A, et al. (1993) High levels of interferon alpha in the sera of children with dengue virus infection. Am J Trop Med Hyg 48: 222-229.

51. Juffrie M, Meer GM, Veerman AJP, Thijs LG, Hack CE (2002) Inflammatory mediators in dengue virus infection: Circulating interleukin-12 and interferon-γ. Dengue Bulletin 26: 144-154.

52. Mustafa AS, Elbishbishi EA, Agarwal R, Chaturvedi UC (2001) Elevated levels of interleukin-13 and IL-18 in patients with dengue hemorrhagic fever. FEMS Immunol Med Microbiol 30: 229-233.

53. Bethell DB, Flobbe K, Cao XT, Day NP, Pham TP, et al. (1998) Pathophysiologic and prognostic role of cytokines in dengue hemorrhagic fever. J Infect Dis 177: 778-782.

54. Valero N, Larreal Y, Espina LM, Reyes I, Maldonado M, et al. (2008) Elevated levels of interleukin-2 receptor and intercellular adhesion molecule 1 in sera from a venezuelan cohort of patients with dengue. Arch Virol 153: 199-203.

55. Koraka P, Murgue B, Deparis X, Van Gorp EC, Setiati TE, et al. (2004) Elevation of soluble VCAM-1 plasma levels in children with acute dengue virus infection of varying severity. J Med Virol 72: 445-450.

56. Sathupan P, Khongphattanayothin A, Srisai J, Srikaew K, Poovorawan Y (2007) The role of vascular endothelial growth factor leading to vascular leakage in children with dengue virus infection. Ann Trop Paediatr 27: 179-184.

57. Agarwal R, Elbishbishi EA, Chaturvedi UC, Nagar R, Mustafa AS (1999) Profile of transforming growth factor-beta 1 in patients with dengue haemorrhagic fever. Int J Exp Pathol 80: 143-149.

58. Mairuhu AT, Peri G, Setiati TE, Hack CE, Koraka P, et al. (2005) Elevated plasma levels of the long pentraxin, pentraxin 3, in severe dengue virus infections. J Med Virol 76: 547-552.

59. Kurane I, Innis BL, Nimmannitya S, Nisalak A, Meager A, et al. (1991) Activation of T lymphocytes in dengue virus infections. High levels of soluble interleukin 2 receptor, soluble CD4, soluble CD8, interleukin 2, and interferon-gamma in sera of children with dengue. J Clin Invest 88: 1473-1480.

60. Chaturvedi UC, Raghupathy R, Pacsa AS, Elbishbishi EA, Agarwal R, et al. (1999) Shift from a Th1-type response to Th2-type in dengue haemorrhagic fever. Current science (Bangalore) 76: 63-69.

61. Preeyasombat C, Treepongkaruna S, Sriphrapradang A, Choubtum L (1999) The role of prostacyclin (PGI2) and thromboxane A2 (TXA2) in pathogenesis of dengue hemorrhagic fever (DHF). J Med Assoc Thai 82 Suppl 1: S16-21.

62. Kalayanarooj S, Gibbons RV, Vaughn D, Green S, Nisalak A, et al. (2007) Blood group AB is associated with increased risk for severe dengue disease in secondary infections. J Infect Dis 195: 1014-1017.

63. Suvarna JC, Rane PP (2009) Serum lipid profile: a predictor of clinical outcome in dengue infection. Trop Med Int Health 14: 576-585.

64. Chaturvedi UC, Elbishbishi EA, Agarwal R, Mustafa AS (2001) Cytotoxic factor-autoantibodies: possible role in the pathogenesis of dengue haemorrhagic fever. FEMS Immunol Med Microbiol 30: 181-186.

65. Juffrie M, Meer GM, Hack CE, Haasnoot K, Sutaryo, et al. (2001) Inflammatory mediators in dengue virus infection in children: interleukin-6 and its relation to C-reactive protein and secretory phospholipase A2. Am J Trop Med Hyg 65: 70-75.

66. Juffrie M, van Der Meer GM, Hack CE, Haasnoot K, Sutaryo, et al. (2000) Inflammatory mediators in dengue virus infection in children: interleukin-8 and its relationship to neutrophil degranulation. Infect Immun 68: 702-707.

67. Ray G, Kumar V, Kapoor AK, Dutta AK, Batra S (1999) Status of antioxidants and other biochemical abnormalities in children with dengue fever. J Trop Pediatr 45: 4-7.

68. Trairatvorakul P, Chongsrisawat V, Ngamvasinont D, Asawarachun D, Nantasook J, et al. (2005) Serum nitric oxide in children with dengue infection. Asian Pac J Allergy Immunol 23: 115-119.

69. Soundravally R, Sankar P, Bobby Z, Hoti SL (2008) Oxidative stress in severe dengue viral infection: association of thrombocytopenia with lipid peroxidation. Platelets 19: 447-454.

70. Rajendiran S, Lakshamanappa HS, Zachariah B, Nambiar S (2008) Desialylation of plasma proteins in severe dengue infection: possible role of oxidative stress. Am J Trop Med Hyg 79: 372-377.

71. Honsawek S, Kongtawelert P, Pothacharoen P, Khongphatthanayothin A, Chongsrisawat V, et al. (2007) Increased levels of serum hyaluronan in patients with dengue infection. J Infect 54: 225-229.

72. Chau TN, Anders KL, Lien le B, Hung NT, Hieu LT, et al. (2010) Clinical and virological features of Dengue in Vietnamese infants. PLoS Negl Trop Dis 4: e657.

73. Kittigul L, Meethien N, Sujirarat D, Kittigul C, Vasanavat S (1997) Comparison of dengue virus antigens in sera and peripheral blood mononuclear cells from dengue infected patients. Asian Pac J Allergy Immunol 15: 187-191.
