# Supplementary material for: Factors Associated with Dengue Shock Syndrome: A Systematic Review and Meta-Analysis
Source: PLoS Negl Trop Dis. 2013 Sep 26;7(9):e2412. doi: 10.1371/journal.pntd.0002412 (PMC3784477; doi:10.1371/journal.pntd.0002412)
Supplement: Table S4 — Meta-analysis of DSS with factors that were investigated in at least two studies. Pooled odds ratios (OR) with corresponding 95% confidence intervals (95%CI) of the published results were calculated where more than one study had investigated the factor. (DOC) [file pntd.0002412.s006.doc]

**Table S4. Meta-analysis of DSS with factors that were investigated in at least two studies.** Pooled odds ratios (OR) with corresponding 95% confidence intervals (95%CI) of the published results were calculated where more than one study had investigated the factor.

| **Variable** | **No. of study** | **Total sample size**  **(DSS/DHF)** | **Heterogeneity** | | **Model** | **Association with DSS** | | | **Egger’s 2-tailed bi**as **p-value** | **Included studies (References list shown in Table S2)** |
| --- | --- | --- | --- | --- | --- | --- | --- | --- | --- | --- |
|  |  |  | p-value | *I^2^* |  | p-value | Effect size (95% CI) | p-value after removing 1 study |  |  |
| **Prevalence of DSS among DHF/DSS** | 80 | 3992/11220 | <0·001 | 95 | Random |  | Even rate  0·285(95% CI: 0·247 - 0·326) |  | 0.88 | [3,4,6,7,8,8,8,9,10,11,13,18,21,23,25,27,28,30,34,36,37,38,40,42,45,46,48,51,54,57,61,73,78,80,82,84,87,88,88,89,91,95,105,106,108,111,114,120,128,134,140,143,145,148,149,150,154,157,158,159,160,165,166,167,168,170,176,177,179,180,183,184,189,192,193,194,195,196,197,198] |
| **Demography** |  |  |  |  |  |  |  |  |  |  |
| Gender (female)^c,#^ | 37 | 1957/4258 | 0·063 | 28 | Random | <0·001 | OR:1·37(1·17-1·60) | <0·001 | 0.63 | [3,5,7,11,29,34,37,40,43,52,54,61,63,68,70,73,82,87,92,95,112,123,128,135,136,152,159,162,168,172,175,179,180,181,188,191,194] |
| Age (year)^†^ | 37 | 2927/6400 | <0·001 | 90 | Random | <0·001 | OR:0·50(0·36-0·70)  OR:0·27(0·17-0·42)^a^ | <0·001 | 0·009 | [4,7,8,11,34,37,43,52,54,62,70,73,77,84,87,92,95,112,123,135,150,152,157,158,162,168,172,175,179,180,181,194,195,196,198] |
| Malnutrition^#^ | 9 | 1689/3449 | 0·37 | 8 | Fixed | 0·05 | OR:1·19(1·00-1·41)  OR:1·37(1·18-1·59)^a^ | 0·863 | 0·03 | [10,40,61,70,73,79,82,172,194] |
| Obesity/overweigh^#^ | 8 | 1513/3187 | 0·05 | 51 | Random | 0·15 | OR:1·31(0·91-1·88) |  | 0·32 | [10,40,70,79,82,133,172,194] |
| Normal nutrition^#^ | 9 | 1616/3398 | 0·26 | 21 | Fixed | 0·03 | OR:0·87(0·77-0·99) | 0·20 | 0·43 | [10,40,61,70,79,82,132,172,194] |
| **Ethnic** |  |  |  |  |  |  |  |  |  |  |
| Black skin^#^ | 2 | 39/140 | 0·87 | 0 | Fixed | 0·34 | OR:0·50(0·12-2·06) |  |  | [45,49] |
| Mixed skin/Mulatto^#^ | 2 | 39/140 | 0·14 | 54 | Fixed | 0·44 | OR:0·66(0·23-1·90) |  |  | [45,49] |
| White skin^#^ | 2 | 39/140 | 0·21 | 36 | Fixed | 0·10 | OR:2·14(0·87-5·25) |  |  | [45,49] |
| Chinese^#^ | 2 | 34/497 | 0·10 | 64 | Random | 0·73 | OR:1·47(0·17-12·9) |  |  | [128,191] |
| Malay^#^ | 2 | 34/497 | 0·43 | 0 | Fixed | 0·10 | OR:0·37(0·11-1·23) |  |  | [128,191] |
| Indian^#^ | 2 | 34/497 | 0·67 | 0 | Fixed | 0·70 | OR:1·35(0·28-6·44) |  |  | [128,191] |
| **Non-specific clinical signs** |  |  |  |  |  |  |  |  |  |  |
| Date of admission from the onset of fever^†^ | 9 | 166/781 | 0·076 | 44 | Random | 0·58 | OR:1·15(0·71-1·86) |  | 0·53 | [4,28,62,102,109,110,179,194,195] |
| Duration of fever* | 20 | 2397/5903 | <0·001 | 87 | Random | 0·52 | OR:1·13(0·80-1·57) |  | 0·44 | [4,7,11,23,37,60,62,70,75,77,82,85,92,95,104,112,157,158,172,193] |
| High temperature^#^ | 2 | 64/169 | <0·001 | 95 | Random | 0·64 | OR:1·35(0·39-4·66) |  |  | [7,40] |
| Headache^#^ | 12 | 330/733 | 0·94 | 0 | Fixed | 0·63 | OR:0·92(0·65-1·30) |  | 0·45 | [4,7,12,29,43,53,53,73,120,123,160,172] |
| Cough^#^ | 7 | 249/362 | 0·27 | 21 | Fixed | 0·19 | OR:1·47(0·79-2·64) |  | 0·67 | [4,20,43,53,53,73,160] |
| Arthralgia^#^ | 4 | 66/254 | 0·036 | 65 | Random | 0·82 | OR:1·22(0·24-6·30) |  |  | [4,7,12,162] |
| Myalgia^#^ | 7 | 1399/3330 | 0·26 | 22 | Fixed | 0·19 | OR:0·70(0·40-1·20) |  | 0·43 | [7,12,53,53,59,77,123] |
| Conjunctival manifestation^#^ | 2 | 17/125 | 0·13 | 57 | Fixed | 0·94 | OR:0·94(0·18- 4·93) |  |  | [123,177] |
| Rash^#^ | 10 | 1443/3455 | 0·57 | 0 | Fixed | 0·089 | OR:0·80(0·62- 1·04)  OR:0·76(0·59- 0·98)^a^ |  | 0·06 | [4,7,12,20,29,77,123,157,158,177] |
| Co-infection^#^ | 4 | 49/179 | 0·03 | 67 | Random | 0·44 | OR:2·12(0·32- 14·1) |  |  | [57,101,120,194] |
| **Neurological signs** |  |  |  |  |  |  |  |  |  |  |
| Any signs^#^ | 15 | 859/1891 | <0·001 | 82 | Random | 0·003 | OR:4·66(1·70-12·8) | <0·01 | 0·42 | [4,20,30,43,53,53,59,62,73,81,120,132,146,157,166,173,197] |
| Convulsion^#^ | 5 | 107/184 | 0·42 | 0 | Fixed | 0·04 | OR:2·48(1·05-5·85) |  | 0·78 | [4,20,59,124,132] |
| Decrease of consciousness^#^ | 6 | 108/228 | 0·055 | 54 | Random | 0·001 | OR:7·35(2·38-22·7) | <0·01 | 0·33 | [4,43,53,53,132,157] |
| Drowsiness^#^ | 3 | 72/224 | 0·16 | 45 | Fixed | <0·001 | OR:5·15(2·12-12·5) |  |  | [20,123,146] |
| Lethargy^#^ | 2 | 59/56 | 0·97 | 0 | Fixed | <0·001 | OR:7·59(0·28-6·44) |  |  | [12,132] |
| **Respiratory failure*** | 4 | 107/252 | 0·30 | 18 | Fixed | <0·001 | OR:3·50(2·08- 5·88) | <0·05 |  | [4,12,62,124] |
| **Increased blood urea or creatinine*** | 6 | 90/459 | 0·001 | 75 | Random | 0·008 | OR:6·26(1·62- 24·1) | <0·05 | 0·76 | [36,105,123,152,166,187] |
| **Cardiac factors** |  |  |  |  |  |  |  |  |  |  |
| Bradycardia^#^ | 3 | 55/84 | 0·44 | 0 | Fixed | 0·81 | OR:1·16(0·35-3·87) |  |  | [20,123,190] |
| Cardiac dysfunction* | 4 | 64/89 | 0·047 | 62 | Random | 0·08 | OR:3·16(0·87- 11·4) |  |  | [72,85,169,190] |
| **Digestive factors** |  |  |  |  |  |  |  |  |  |  |
| Vomiting/Nausea^#^ | 14 | 839/1391 | 0·42 | 3 | Fixed | 0·001 | OR:1·43(1·15-1·78) | <0·01 | 0·82 | [4,7,12,37,43,70,73,82,123,132,146,157,158,172] |
| Abdominal pain^#^ | 17 | 2340/4986 | 0·014 | 48 | Random | <0·001 | OR:2·26(1·76-2·89) | <0·001 | 0·17 | [7,12,20,29,37,40,43,53,53,70,73,77,82,123,132,172,181] |
| Abdominal tendernessa^#^ | 3 | 1182/3375 | 0·001 | 87 | Random | 0·057 | OR:3·45(0·96-12·4) |  |  | [12,146,174] |
| Diarrhea^#^ | 7 | 151/390 | 0·12 | 40 | Fixed | 0·57 | OR:1·17(0·69-1·98)  OR: 0·93(0·56-1·54)^a^ |  | 0·06 | [4,7,12,132,157,172,194] |
| Hyperlipase or hyperamylase^#^ | 2 | 15/76 | 0·31 | 4 | Fixed | 0·13 | OR:3·57(0·69-18·5) |  |  | [103,156] |
| **Bleeding signs** |  |  |  |  |  |  |  |  |  |  |
| Tourniquet positive^#^ | 16 | 1698/3863 | <0·001 | 69 | Random | 0·058 | OR:0·63(0·39- 1·02) |  | 0·55 | [4,7,13,39,52,64,73,75,77,97,123,152,160,172,192,193] |
| Skin hemorrhages (max)^b,#^ | 16 | 530/1702 | 0·004 | 69 | Random | 0·124 | OR:1·48(0·90- 2·43) |  | 0·21 | [4,7,11,12,60,73,96,97,120,135,152,160,177,181,192,195] |
| Skin hemorrhages (min)^#^ | 16 | 530/1702 | 0·35 | 9 | Fixed | 0·66 | OR:1·06(0·82- 1·37) |  | 0·85 | [4,7,11,12,60,73,96,97,120,135,152,160,177,181,192,195] |
| Petechiae^#^ | 11 | 284/560 | 0·34 | 11 | Fixed | 0·45 | OR:1·18(0·78- 1·78) |  | 0·77 | [7,12,60,73,96,120,135,152,160,177,192] |
| Ecchymose/purpura^#^ | 8 | 283/663 | <0·001 | 75 | Random | 0·139 | OR:1·47(0·88- 2·50) |  | 0·72 | [7,11,12,28,73,120,152,177] |
| Gum bleeding^#^ | 9 | 139/415 | 0·114 | 38 | Fixed | 0·84 | OR:1·07(0·56- 2·07) |  | 0·84 | [4,7,43,53,53,96,132,152,172] |
| Nose bleeding^#^ | 13 | 377/681 | 0·52 | 0 | Fixed | 0·90 | OR:1·02(0·71-1·47)  OR: 1·21(0·86-1·70)^a^ |  | 0·07 | [4,7,11,12,43,53,53,73,132,135,152,172,192] |
| Gastrointestinal bleeding^#^ | 18 | 786/1317 | 0·52 | 0 | Fixed | <0·001 | OR:1·84(1·42- 2·39) | <0·001 | 0·58 | [4,7,11,12,37,43,62,73,96,97,120,124,132,135,146,152,172,192] |
| Hematuria^#^ | 5 | 140/312 | 0·132 | 44 | Fixed | 0·46 | OR:0·63(0·18- 2·17) |  | 0·36 | [4,7,73,111,152] |
| Hemoptysis^#^ | 3 | 48/172 | 0·64 | 0 | Fixed | 0·65 | OR:1·50(0·26- 8·77) |  |  | [7,132,152] |
| DIC^#^ | 2 | 29/32 | 0·95 | 0 | Fixed | 0·006 | OR:4·69(1·55- 14·2) |  |  | [163,173] |
| **Plasma leakage** |  |  |  |  |  |  |  |  |  |  |
| Hemoconcentration* | 38 | 2847/5214 | <0·001 | 71 | Random | <0·001 | OR:2·61(2·02- 3·37) | <0·001 | 0·54 | [4,9,11,12,17,20,29,36,39,40,41,43,62,70,73,77,82,88,88,97,100,109,112,123,124,132,135,142,143,157,158,164,172,180,192,193,194,198] |
| Pleural effusion* | 18 | 1757/3860 | <0·001 | 77 | Random | <0·001 | OR:10·4(5·47- 19·6)  OR:17·8(8·46- 37·5)^a^ | <0·001 | 0·07 | [4,7,20,29,64,73,77,96,97,124,126,152,155,165,172,173,188,194] |
| Ascites^#^ | 12 | 373/763 | <0·001 | 76 | Random | <0·001 | OR:5·92(5·42- 14·5) | <0·001 | 0·99 | [4,7,20,29,73,97,146,152,155,157,172,173] |
| Splenomegaly^#^ | 7 | 299/453 | 0·034 | 56 | Random | 0·40 | OR:1·52(0·58- 3·96) |  | 0·14 | [20,172,7,158,4,73,155] |
| Gallbladder swelling* | 4 | 110/113 | <0·001 | 83 | Random | 0·013 | OR:14·9(1·76- 128) |  |  | [20,48,155,165] |
| Hypoalbuminemia* | 13 | 1662/3461 | <0·001 | 81 | Random | <0·001 | OR:4·34(2·51- 7·52) | <0·001 | 0·34 | [11,20,30,58,65,77,95,126,131,134,144,180,184] |
| Hypoproteinemia* | 8 | 178/276 | 0·021 | 58 | Random | 0·009 | OR:2·45(1·25- 4·81) | <0·05 | 0·35 | [36,97,126,134,144,163,168,184] |
| **Blood electrolyte** |  |  |  |  |  |  |  |  |  |  |
| Hyponatremia (min)* | 6 | 140/368 | 0·012 | 66 | Random | 0·017 | OR:2·81(1·20- 6·58) | <0·071 | 0·63 | [20,36,111,116,172,187] |
| Hyponatremia (max)* | 6 | 140/368 | 0·060 | 53 | Random | <0·001 | OR:3·78(1·87- 7·65) | <0·002 | 0·30 | [20,36,83,111,187] |
| Hypokalemia* | 3 | 56/115 | 0·033 | 71 | Random | 0·96 | OR:0·96(0·23- 4·01) |  |  | [20,111,187] |
| Venous blood lactate^†^ | 2 | 49/46 | 0·39 | 0 | Fixed | <0·001 | OR:9·11(4·11- 20·2) |  |  | [12,140] |
| Hyperventilation (low PaCO2)* | 2 | 56/60 | 0·052 | 73 | Random | 0·39 | OR:2·84(0·27- 30·4) |  |  | [83,187] |
| Acidosis (min)* | 5 | 118/269 | 0·041 | 60 | Random | 0·044 | OR:2·84(1·03- 7·82) | <0·051 | 0·72 | [20,36,83,111,187] |
| Acidosis (max)* | 5 | 118/269 | 0·033 | 62 | Random | 0·030 | OR:2·72(1·10- 6·73) | <0·05 | 0·95 | [20,36,83,111,187] |
| **Urinalysis** |  |  |  |  |  |  |  |  |  |  |
| Proteinuria/albuminuria^#^ | 5 | 63/270 | 0·76 | 0 | Fixed | 0·49 | OR:1·29(0·63- 2·65) |  | 0·51 | [36,111,123,124,152] |
| **Hepatic manifestations** |  |  |  |  |  |  |  |  |  |  |
| Hepatomegaly* | 28 | 4130/8906 | <0·001 | 84 | Random | <0·001 | OR:3·10(2·18- 4·41) | <0·001 | 0·19 | [4,7,11,12,13,20,21,28,37,39,40,43,70,73,77,82,100,104,123,132,155,158,160,172,173,174,181,194] |
| Jaundice^#^ | 4 | 252/655 | 0·38 | 87 | Fixed | 0·148 | OR:2·37(0·74-7·63) |  |  | [4,73,166,181] |
| ALT* | 26 | 2772/6281 | <0·001 | 82 | Random | <0·001 | OR:2·15(1·47- 3·15) | <0·001 | 0·21 | [20,30,31,36,58,60,65,69,75,77,104,118,123,125,126,128,131,134,144,157,158,166,172,180,184] 186] |
| AST* | 26 | 2772/6281 | <0·001 | 89 | Random | <0·001 | OR:2·08(1·39- 3·12) | <0·005 | 0·13 | [20,30,31,36,58,60,65,69,75,77,104,118,123,125,126,128,131,134,144,157,158,166,172,180,184] 186] |
| Alkaline phosphatase (ALP)* | 5 | 93/119 | 0·35 | 9 | Fixed | 0·71 | OR:1·13(0·61- 2·08)  OR:0·85(0·49- 1·48)^a^ |  | 0·04 | [30,118,123,131,184] |
| Total bilirubin* | 4 | 85/75 | <0·001 | 85 | Random | 0·78 | OR:1·39(0·14- 13·8) |  | 0·88 | [30,31,65,131] |
| Direct bilirubin^†^ | 2 | 69/31 | <0.001 | 92 | Random | 0.50 | OR:0.21(0.002- 20.7) |  |  | [31,131] |
| **Blood cells** |  |  |  |  |  |  |  |  |  |  |
| Thrombocytopenia  (Low platelet count)* | 47 | 2801/7172 | <0.001 | 79 | Random | <0.001 | OR:2.64(1.95- 3.59) | <0.001 | 0.15 | [4,11,12,20,23,29,39,40,41,43,52,62,64,70,73,77,82,84,85,88,88,97,104,110,112,120,123,124,132,144,152,157,158,162,163,164,167,172,107,173,179,181,186,192,193,194,195] |
| Erythrocyte sedimentation rate* | 3 | 129/180 | <0.001 | 85 | Random | 0.4 | OR:0.87(0.24- 3.24) |  |  | [59,74,162] |
| WBC^†^ | 15 | 1627/3716 | <0.001 | 85 | Random | 0.78 | OR:1.08(0.62- 1.89) |  | 0.13 | [11,12,41,43,62,75,77,95,104,157,158,172, 186,194,198] |
| Leukopenia  (WBC<4-5,000)^#^ | 9 | 1539/3525 | <0.001 | 80 | Random | 0.64 | OR:0.84(0.41- 1.74) |  | 0.23 | [4,20,40,43,75,77,146,158,172] |
| Leukocytosis (WBC>12,000)^#^ | 2 | 18/62 | 0.64 | 0 | Fixed | <0.001 | OR:11.4(3.39- 38.1) |  |  | [43,124] |
| CD3+ T cells^†^ | 2 | 27/26 | 0.003 | 88 | Random | 0.87 | OR:1.23(0.05- 31.4) |  |  | [110,151] |
| CD3+CD4+ Tcells^†^ | 2 | 27/26 | 0.196 | 40 | Fixed | 0.32 | OR:0.32(0.12- 0.89) |  |  | [110,151] |
| CD3+CD8+ Tcells^†^ | 2 | 27/26 | 0.007 | 86 | Random | 0.56 | OR:2.44(0.12- 48) |  |  | [110,151] |
| CD19+ B cells (B1)^†^ | 2 | 28/26 | 0.48 | 0 | Fixed | 0.21 | OR:1.87(0.71- 4.98) |  |  | [110,151] |
| **Coagulators** |  |  |  |  |  |  |  |  |  |  |
| Prothrombin time (min)* | 15 | 1661/3713 | <0.001 | 68 | Random | <0.001 | OR:2.83(1.84- 4.37) | <0.001 | 0.96 | [20,29,30,31,52,60,64,77,120,131,157,158,172,181,195] |
| APTT (min)* | 13 | 1557/3678 | <0.001 | 93 | Random | <0.001 | OR:6.81(2.83- 16.4)  OR:5.18(2.19- 12.2)^a^ | <0.001 | 0.017 | [29,60,64,77,95,96,157,158,163,164,172,181,186,195] |
| Thrombin time* | 3 | 1351/3039 | <0.001 | 88 | Random | 0.044 | OR:5.17(1.05- 26) | <0.001 |  | [64,77,164] |
| Fibrinogen level* | 9 | 185/456 | <0.001 | 83 | Random | <0.001 | OR:0.13(0.05- 0.35) | 0.001 | 0.53 | [52,60,64,96,163,164,181,192,195] |
| Fibrinogen degraded product (FDP)* | 4 | 70/283 | 0.008 | 75 | Random | 0.12 | OR:2.56(0.78- 8.40) |  |  | [64,163,171,195] |
| Decreased factor V* | 2 | 24/47 | 0·15 | 52 | Fixed | 0·42 | OR:2·86(0·23- 36) |  |  | [36,117] |
| Decreased factor VII* | 2 | 20/43 | 0·075 | 69 | Random | 0·24 | OR:6·46(0·28- 147) |  |  | [36,117] |
| Decreased factor VIII* | 2 | 23/23 | 0·89 | 0 | Fixed | 0·046 | OR:5·70(1·03- 31·6) |  |  | [64,117] |
| Decreased factor IX* | 2 | 20/43 | 0·118 | 59 | Fixed | 0·061 | OR:7·69(0·91- 65) |  |  | [36,117] |
| Decreased factor X* | 2 | 20/43 | 0·29 | 12 | Fixed | 0·039 | OR:9·76(1·12- 85) |  |  | [36,117] |
| **Viral factors** |  |  |  |  |  |  |  |  |  |  |
| DENV-1^#^ | 17 | 962/2111 | 0·004 | 54 | Random | 0·18 | OR:0·74(0·48- 1·15) |  | 0·70 | [28,38,47,50,51,76,86,87,100,106,110,133,149,150,167,176,183] |
| DENV-2^#^ | 20 | 1008/2240 | <0·001 | 62 | Random | 0·019 | OR:1·66(1·09- 2·55) | 0·064 | 0·91 | [28,38,50,51,54,76,86,87,100,106,110,120,133,149,150,167,176,179,183,196] |
| DENV-3^#^ | 19 | 1013/2202 | 0·038 | 40 | Random | 0·77 | OR:0·94(0·64- 1·38) |  | 0·39 | [28,38,47,50,51,54,76,86,100,106,110,120,133,149,150,167,176,183,196] |
| DENV-4^#^ | 14 | 922/2023 | 0·51 | 0 | Fixed | 0·15 | OR:0·72(0·46- 1·12) |  | 0·88 | [28,38,47,50,76,86,100,106,110,133,149,150,167,176] |
| Viremia* | 3 | 54/215 | 0·008 | 79 | Random | 0·63 | OR:0·69(0·16- 3·09) |  |  | [16,28,110] |
| Dengue antigen on leukocytes* | 4 | 68/156 | 0·93 | 0 | Fixed | 0·81 | OR:0·92(0·46- 1·83) |  |  | [15,34,87,89] |
| Primary infection^#^ | 37 | 1251/2696 | 0·67 | 0 | Fixed | <0·001 | OR:0·47(0·37- 0·60) | <0·001 | 0·76 | [6,15,18,18,34,42,44,44,49,51,54,60,80,87,89,91,93,97,106,108,115,120,130,133,135,139,145,150,154,162,176,183,188,189,191,192,196] |
| Secondary infection^#^ | 40 | 1731/2989 | <0·001 | 57 | Random | 0·001 | OR:1·75(1·26- 2·42) | <0·005 | 0·84 | [15,18,34,42,44,49,51,52,54,60,80,87,89,91,93,100,106,108,115,120,130,132,133,135,139,145,150,154,162,172,176,179,183,188,189,191,192,196] |
| **Biomarkers** |  |  |  |  |  |  |  |  |  |  |
| Complement C3* | 5 | 90/202 | 0·008 | 71 | Random | 0·006 | OR:0·24(0·09- 0·67)  OR:0·15(0·05- 0·49)^a^ | 0·1 | 0·08 | [35,64,127,171,195] |
| Complement C4^†^ | 2 | 46/23 | 0·12 | 59 | Fixed | 0·03 | OR:0·36(0·14- 0·91) |  |  | [127,192] |
| Complement C5^†^ | 2 | 46/23 | 0·83 | 0 | Fixed | <0·001 | OR:0·16(0·06- 0·42) |  |  | [127,192] |
| Complement C6^†^ | 2 | 46/23 | 0·012 | 84 | Random | 0·30 | OR:0·20(0·01- 4·14) |  |  | [127,192] |
| Complement C8^†^ | 2 | 46/23 | 0·26 | 22 | Fixed | 0·30 | OR:0·62(0·25- 1·55) |  |  | [127,192] |
| Complement C9^†^ | 2 | 46/23 | 0·36 | 0 | Fixed | 0·011 | OR:0·30(0·12- 0·76) |  |  | [127,192] |
| Dengue specific IgM^†^ | 3 | 100/60 | 0·051 | 66 | Random | 0·42 | OR:1·03(0·33- 3·19) |  |  | [92,157,158] |
| Dengue specific IgA* | 2 | 82/143 | 0·24 | 27 | Fixed | 0·46 | OR:0·75(0·36- 1·60) |  |  | [92,145] |
| Prostacyclin (PGI2)^†^ | 2 | 65/82 | 0·24 | 28 | Fixed | <0·001 | OR:6389(1984- 20579) |  |  | [137,138] |
| Total cholesterol^†^ | 3 | 73/81 | 049 | 0 | Fixed | <0·001 | OR:0·21(0·11- 0·42) | <0·05 |  | [46,144,170] |
| Triglyceride^†^ | 3 | 73/81 | 0·004 | 82 | Random | 0·99 | OR:0·99(0·20- 4·85) |  |  | [46,144,170] |
| HDL^†^ | 2 | 63/39 | <0·001 | 95 | Random | 0·20 | OR:0·06(0·001- 4·17) |  |  | [46,170] |
| LDL^†^ | 2 | 63/39 | 0·123 | 58 | Fixed | 0·012 | OR:0·37(0·17- 0·80) |  |  | [46,170] |
| Human cytotoxic factor in serum^#^ | 2 | 69/264 | 0·036 | 77 | Random | 0·46 | OR:3·10(0·15- 64) |  |  | [24,119] |
| TNF-α^†^ | 7 | 59/176 | 0·56 | 0 | Fixed | 0·019 | OR:1·50(1·07- 2·11) | <0·05 | 0·13 | [27,55,60,90,104] |
| IFN-γ (max)* | 5 | 95/145 | 0·39 | 3 | Fixed | 0·29 | OR:0·75(0·43- 1·28) |  | 0·59 | [25,60,69,98,104] |
| IFN-γ (min)* | 5 | 95/145 | 0·40 | 1 | Fixed | 0·037 | OR:0·57(0·34- 0·97) |  | 0·96 | [25,60,69,98,104] |
| IL-2* | 3 | 33/31 | 0·049 | 67 | Random | 0·88 | OR:1·11(0·29- 4·21) |  |  | [25,60,98] |
| IL-4* | 2 | 54/90 | 0·081 | 67 | Random | 0·57 | OR:1·39(0·43- 4·49) |  |  | [25,60] |
| IL-6 (min)* | 7 | 178/402 | <0·001 | 88 | Random | 0·007 | OR:5·43(1·59- 18·5) | <0·05 | 0·14 | [11,25,27,55,60,68,104] |
| IL-8^†^ | 3 | 69/143 | <0·001 | 98 | Random | 0·084 | OR:114(0·53- 24303) |  |  | [27,67,141] |
| IL-10^†^ | 4 | 64/143 | 0·066 | 58 | Random | 0·325 | OR:1·65(0·61- 4·50) |  |  | [25,27,60,104] |
| IL-12* | 2 | 55/63 | 0.1 | 63 | Random | 0.077 | OR:0·12(0·01- 1·26) |  |  | [69,129] |
| IP-10 (interferon-inducible protein-10)^†^ | 2 | 11/78 | 0·23 | 32 | Fixed | 0·71 | OR:1·26(0·39- 4·07) |  |  | [27,104] |
| Soluble IL-2 receptor^†^ | 2 | 10/15 | 0·023 | 81 | Random | 0·205 | OR:12·7(0·25- 654) |  |  | [98,186] |
| MCP-1 (monocyte chemotactic protein)^†^ | 2 | 16/93 | 0·025 | 80 | Random | 0·21 | OR:4·31(0·44- 42) |  |  | [27,102] |

*Factor was presented as both dichotomous (frequency of higher values) and continuous (higher value) variables.

^#^Factor was presented as a dichotomous (frequency of higher values) variable.

^†^Factor was presented as a continuous variable.

^a^OR: adjusted odds ratio calculated after the addition of potential missing studies using the trim and fill method of Duvall and Tweedie.

^b^min and max: when there were more than one data or type of data for particular factor, the data with lowest and highest were pooled separately to get minimal and maximal odd ratios, respectively.

^c^Factors, which were fully analyzed and interpreted in the full text, were highlighted in blue color when more than ten studies for particular factor.

Acidosis was defined as any sign of low blood pH or low bicarbonate level.

Acute renal failure was defined as any sign of increased blood urea or creatinine.

Cardiac dysfunction was defined as any sign of low value of left ventricular ejection fraction or cardiac index, which were performed using ultrasound.

Co-infection was defined as any sign of otitis, bronchitis, pneumonia, septicemia, meningitis, and found as a non associated factor in DSS.

Neurological signs were defined as any sign of restlessness, irritability, dizziness, drowsiness, stupor, coma and convulsion.

Gallbladder swelling was defined as a thicker wall of the gallbladder and presented as both dichotomous (frequency of higher values) and continuous (higher value) variables.

Hemoconcentration was defined as an increase of hematocrit and presented as both dichotomous (frequency of higher values) and continuous (higher value) variables.

Neurological signs (any signs) were defined as patients had any signs of convulsion, decreased consciousness, drowsiness, and lethargy.

Respiratory failure was defined as any sign of short of breath, change of respiratory rate, and respiratory difficulty, which were and presented as both dichotomous (frequency of higher values) and continuous (higher value) variables.

Skin hemorrhages was defined as any sign of petechiae, purpura and echymose.

A particular dengue serotype infection was defined as a dichotomous variable versus infection with another DENV serotype (e.g., DENV-2 vs. non-DENV-2). Only studies investigated all four strains were included for the analysis.
