# Supplementary material for: Factors Associated with Dengue Shock Syndrome: A Systematic Review and Meta-Analysis
Source: PLoS Negl Trop Dis. 2013 Sep 26;7(9):e2412. doi: 10.1371/journal.pntd.0002412 (PMC3784477; doi:10.1371/journal.pntd.0002412)
Supplement: Table S5 — Sensitivity and sub-analysis of co-variables on the effect size and heterogeneity. (DOC) [file pntd.0002412.s007.doc]

**Table S5. Sensitivity and sub-analysis of co-variables on the effect size and heterogeneity.**

| **Variable** | Quality of study | Publication year | Recruite year | Sample size | Estimated data | Area/  country | Cases/  control | Include  DF in  DHF | Prospective/ retrospective | Consecutive/ random | Confirmed diagnosis | Criteria of DSS | Definition of factors^d^ | Population subgroup |
| --- | --- | --- | --- | --- | --- | --- | --- | --- | --- | --- | --- | --- | --- | --- |
| **Gender (female)** | No^a^ | No | No | No |  | No  Homo^b^ | No  Hetero^c^ | No  Hetero | No  Homo | No  Hetero | No  Hetero | No  Hetero |  | No  Hetero |
| **Age** | No | No | No | No | No  Hetero | No  Hetero | No  Hetero | No  Hetero | No  Hetero | No  Hetero | No  Hetero | No  Hetero |  | No  Hetero |
| **Nutritional status** | No | No | No | No | No | No  Homo | No  Homo | No  Homo | No  Homo | No  Homo | No  Homo | No  Homo | No  Homo | No  Homo |
| **Duration of fever** | No | No | No | No | No | No  Hetero | No  Hetero | No  Hetero | No  Hetero | No  Hetero | No  Hetero | No  Hetero |  | No  Hetero |
| **Neurological signs** | No | No | No | No |  | No  Homo | No  Homo | No  Hetero | No  Hetero | Yes  Homo | No  Hetero | No  Hetero |  | No  Hetero |
| **Abdominal pain** | Yes | Yes | No | No |  | No  Hetero | No  Hetero | No  Hetero | No  Hetero | No  Hetero | No  Hetero | No  Homo |  | No  Hetero |
| **Hemoconcentration** | No | No | No | No | No | No  Homo | No  Hetero | No  Hetero | No  Hetero | No  Hetero | No  Hetero | No  Hetero | No  Hetero | No  Hetero |
| **Pleural effusion** | No | No | No | No |  | No  Hetero | No  Hetero | No  Hetero | No  Hetero | No  Hetero | No  Homo | No  Hetero | No  Hetero | No  Hetero |
| **Ascites** | No | No | No | No |  | No  Hetero | No  Hetero | No  Hetero | No  Hetero | No  Hetero | No  Hetero | No  Hetero | No  Hetero | No  Hetero |
| **Hepatomegaly** | No | No | No | No |  | No  Hetero | No  Hetero | No  Hetero | No  Hetero | No  Hetero | No  Hetero | No  Hetero | No  Hetero | No  Hetero |
| **ALT** | No | No | No | No | No | No  Hetero | No  Hetero | No  Hetero | No  Hetero | No  Hetero | No  Hetero | No  Hetero | No  Hetero | No  Hetero |
| **AST** | No | Yes | Yes | No | No | No  Homo | No  Hetero | No  Hetero | No  Hetero | No  Hetero | No  Hetero | No  Hetero | No  Hetero | No  Hetero |
| **Thrombocytopenia** | No | No | No | No | No | No  Hetero | No  Hetero | No  Hetero | No  Hetero | No  Hetero | No  Hetero | No  Hetero | No  Hetero | No  Hetero |
| **Prothrombin time** | No | No | No | No | No | No  Hetero | No  Hetero | No  Hetero | No  Hetero | No  Hetero | No  Hetero | No  Hetero | No  Hetero | No  Homo |
| **APTT** | No | No | No | No | No | No  Hetero | No  Hetero | No  Hetero | No  Hetero | No  Hetero | No  Hetero | No  Hetero | No  Hetero | No  Homo |
| **DENV-1** | No | No | No | No |  | No  Homo | No  Hetero | No  Hetero | No  Homo | No  Hetero | No  Hetero | No  Homo |  | No  Hetero |
| **DENV-2** | No | No | No | No |  | No  Homo | NS  Hetero | No  Hetero | NS  Hetero | NS  Hetero | NS  Hetero | No  Hetero |  | No  Hetero |
| **DENV-3** | No | No | No | No |  | No  Hetero | No  Hetero | No  Hetero | No  Hetero | No  Hetero | No  Hetero | No  Hetero |  | No  Homo |
| **DENV-4** | No | No | No | No |  | No  Hetero | No  Homo | No  Homo | No  Homo | No  Homo | No  Homo | No  Homo |  | No  Homo |
| **Primary infection** | No | No | No | Yes |  | No  Homo | No  Homo | No  Homo | No  Homo | No  Homo | No  Homo | No  Homo |  | No  Homo |
| **Secondary infection** | No | No | No | No |  | No  Hetero | No  Hetero | No  Hetero | No  Hetero | No  Hetero | No  Hetero | No  Hetero |  | No  Hetero |
| **Hypoalbuminemia** | ↓ | ↓ | ↓ | No | No  Homo | No  Homo | No  Homo | No  Hetero | No  Hetero | No  Hetero | No  Homo | No  Hetero | No  Hetero | No  Hetero |

^a^No: no effect of co-variables on the result; Yes: there was a significant effect on the result by indicated co-variable

^b^Homo: homogenous result in the subgroup meta-analysis

^c^Hetero: heterogeneity result in the subgroup meta-analysis

^d^Definition of factors: variations in definition of factors between studies including category/continuous variables, diagnosis, and unit of measurement between studies
